# On-premise medical AI agents for reliable clinical decision-making

**DOI:** 10.1038/s41591-026-04609-x

**Published:** 2026-09-15

**Authors:** Li Zhang, Georg Wölflein, Dyke Ferber, Junhao Liang, Zunamys I. Carrero, Xuewei Wu, Julien Vibert, Jan Clusmann, Lino Möhrmann, Elena E. Möhrmann, Catharina Wichmann, Fabian Wolf, Tim Lenz, Jakob Nikolas Kather

**Affiliations:** 1 Else Kroener Fresenius Center for Digital Health, Faculty of Medicine, TUD Dresden University of Technology, Dresden, Germany; 2 Medical Oncology, National Center for Tumor Diseases (NCT), University Hospital Heidelberg, Heidelberg, Germany; 3 Department of Radiology, The First Affiliated Hospital of Jinan University, Guangzhou, China; 4 Drug Development Department, Gustave Roussy, Villejuif, France; 5 Université Paris-Saclay, Gustave Roussy, INSERM U981, IHU PRISM, Villejuif, France; 6 Department of Medicine I, Faculty of Medicine, TUD Dresden University of Technology, Dresden, Germany; 7 Department of Gynecology and Obstetrics, Faculty of Medicine, TUD Dresden University of Technology, Dresden, Germany; 8 Pathology & Data Analytics, Leeds Institute of Medical Research at St. James’s, University of Leeds, Leeds, UK

**Keywords:** Preclinical research, Diseases

## Abstract

Autonomous clinical artificial intelligence (AI) agents powered by large language models (LLMs), meaning systems that can complete a diagnostic workflow without continuous human input, are increasingly capable of supporting complex reasoning and decision-making. Clinical translation, however, remains limited by two unmet requirements: institutionally governed deployment and reliable decision-time uncertainty estimation. Here we developed and evaluated a fully on-premise clinical agent that couples local operational control with a multi-perspective reliability framework to support selective autonomy. Across two Medical Information Mart for Intensive Care IV (MIMIC-IV)-derived benchmarks, the agent achieved 90.04% accuracy on a seven-disease task and 83.8% accuracy on a four-disease task, approaching a cloud baseline on the primary benchmark. To assess decision-time reliability, we quantified internal-likelihood, language-based and behavioral-stability measures for diagnosis and reasoning. Diagnostic behavioral consistency provided the strongest discrimination of correctness (area under the curve (AUC) = 0.860) and remained informative under stress testing (AUC = 0.875). At a consistency threshold of 0.90, 49.4% of cases were retained at 98.9% diagnostic accuracy. These findings support a practical framework for institutionally governed clinical agents in which decision-time reliability signals identify a lower-risk subset for autonomous handling and defer the remainder for review.

## Abstract (web summary)

An autonomous clinical AI agent enhances decision-making through on-premise deployment and reliability metrics, achieving high diagnostic accuracy and selective autonomy.

## Main

Autonomous AI agents powered by LLMs can now conduct multi-turn clinical dialogues, gather diagnostic evidence and produce diagnoses with accompanying rationales^1–4^. However, clinical translation depends not only on diagnostic capability but also on safe institutional governance and appropriate clinician reliance^5,6^. We, therefore, distinguish two dimensions of trust: operational and decisional. Operational trust concerns governance of data and models, including privacy, auditability, version stability and deployment control, motivating on-premise or similarly governed deployment in healthcare^7–9^. Decisional trust concerns whether an agent’s outputs are reliable enough to support appropriate clinician reliance and, therefore, requires mechanisms for estimating decision-time uncertainty in high-stakes settings^5,10,11^. Regulatory and implementation frameworks reinforce both needs by emphasizing protected handling of health data, governance of AI-enabled software changes across the lifecycle and the practical tradeoffs among control, collaboration, cost and security in healthcare deployment^9,12–15^.

However, operational trust alone is not sufficient for clinical safety. Even a well-governed deployment must support appropriate reliance: clinicians need to know which outputs may be suitable for autonomous handling within a governed workflow and which should be deferred for clinician review^6,16–19^. Consequently, evaluation must move beyond diagnostic accuracy toward a broader assessment of reliability and trustworthiness^6,16–19^. This shift matters because, in healthcare, harm depends not only on average performance but also on whether an agent handles uncertainty appropriately, routing unstable or unreliable outputs toward human oversight rather than presenting them as definitive decisions^5,6,18^. Safety, therefore, depends on reliable decision-time signals indicating when to trust, verify or defer^5^.

These risks are amplified by the technical nature of foundation model agents. LLMs are non-deterministic by default: the same input can yield different outputs under common sampling settings, even when reproducibility controls are used^20^. In agentic systems, small stochastic differences can propagate across multi-step reasoning and tool use, producing divergent intermediate states and unsafe conclusions^21^. Beyond stochastic variation, recent evidence demonstrates that LLMs can amplify biases at scale: sociodemographic labels and patient characteristics can shift clinical recommendations in medically unjustified ways, raising the possibility that case-level uncertainty may aggregate into subgroup-level disparities after deployment^22^. These risks make uncertainty estimation and calibration safety functions rather than optional features^6,11,23^.

Although confidence estimation and calibration in LLMs are increasingly acknowledged^11^, their integration into agent evaluation remains limited. Existing approaches often rely on relatively simple proxies, such as raw model probabilities or heuristic linguistic cues, and have not been systematically evaluated in multi-step clinical agent workflows^11,24,25^. Recent work has addressed related questions through semantic entropy, confidence-weighted self-consistency and uncertainty-aware clinical diagnosis but mostly in single-turn settings such as factual question answering, chain-of-thought reasoning or static clinical note diagnosis, rather than in multi-step agentic workflows, where uncertainty propagates across iterative tool use, intermediate reasoning and interactive information gathering^26–28^. A standardized framework is, therefore, needed to characterize how these diverse confidence signals behave in agentic clinical settings and to identify which observable signals most reliably indicate correctness and whether such signals can support selective autonomy under continued institutional governance^11^.

We studied these two dimensions of trust in an integrated framework: an institutionally governed medical AI agent that can be deployed on-premise and evaluated through inference-time reliability signals. First, we address operational trust by implementing the system in a clinically governable deployment setting and evaluating its end-to-end performance with competitive open-weight LLMs. Second, we address decisional trust by introducing a multi-perspective confidence framework that decomposes reliability into three complementary perspectives: internal likelihood, expressed uncertainty in language and behavioral stability across repeated runs. We then test how these signals behave under baseline, perturbed and external-benchmark conditions and whether they can support selective autonomy through confidence-based triage. Together, these analyses show that interpretable inference-time reliability signals can be coupled to institutionally governed deployment while maintaining competitive performance. They also support selective-autonomy routing by helping distinguish cases suitable for autonomous handling under continued governance from those requiring clinician review.

## Results

### On-premise agent achieves competitive diagnostic performance

**Fig. 1:**
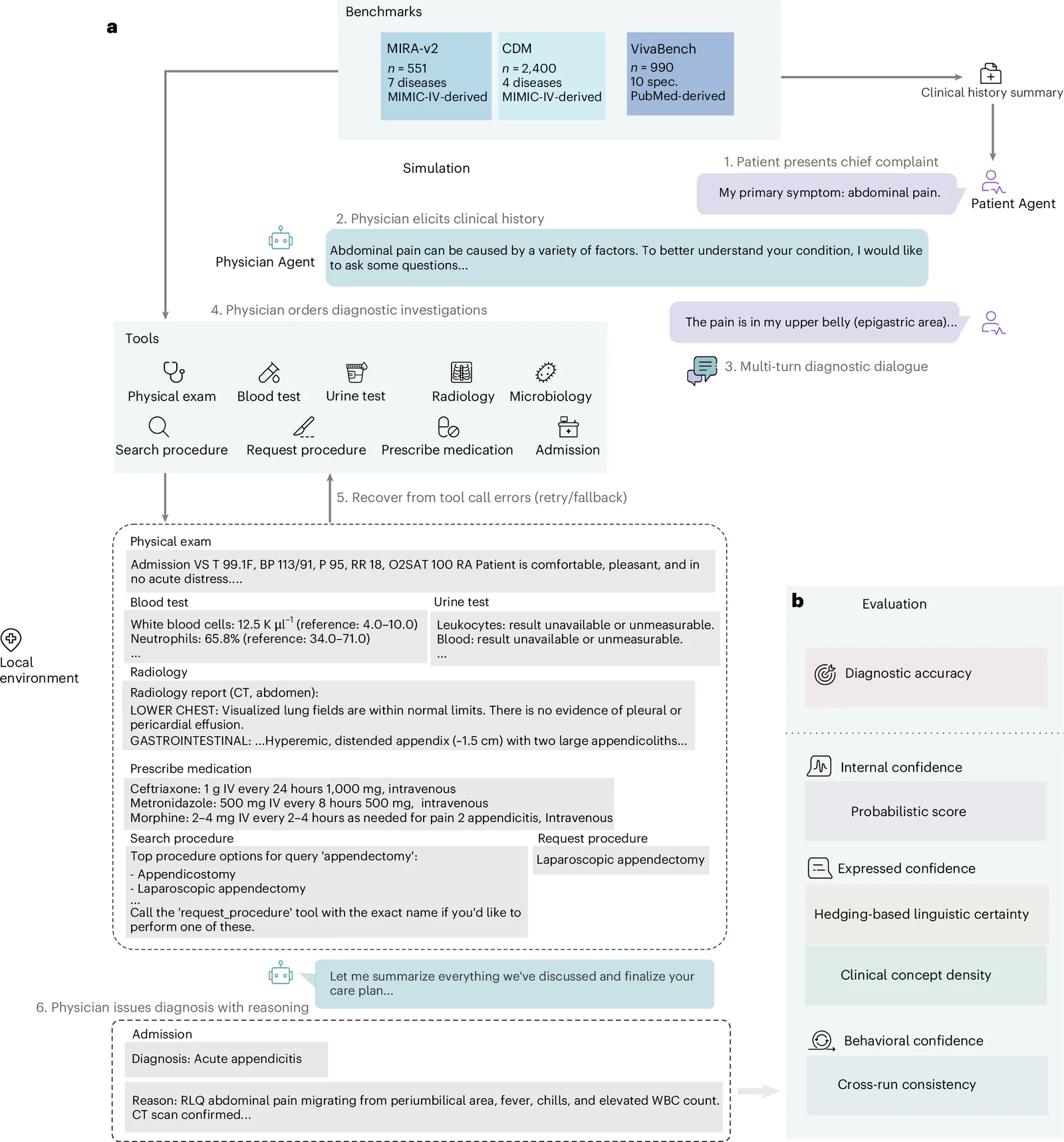
On-premise autonomous clinical agent: simulation architecture and evaluation framework. **a**, Simulation architecture and clinical encounter workflow. The agent conducts multi-turn simulated encounters in a fully local environment (steps 1−6), beginning with the patient’s chief complaint and proceeding through iterative dialogue and structured clinical tool use (physical examination, blood tests, urine tests, radiology, microbiology, medication prescribing and procedure ordering). The encounter concludes with a diagnostic admission decision producing both a diagnosis and a reasoning trace. Encounters are instantiated from three benchmarks: MIRA-v2, CDM and VivaBench. **b**, Evaluation framework. System outputs are assessed along two axes. Diagnostic accuracy is evaluated against reference labels. A multi-perspective confidence analysis decomposes the agent’s apparent certainty into three layers: Internal Confidence (probabilistic score), Expressed Confidence (hedging-based linguistic certainty; clinical concept density) and Behavioral Confidence (cross-run consistency; computed across *N* = 5 independent stochastic runs per case). This dual-stream design yields eight confidence scores in total (four metrics × two streams), enabling separate assessment of whether the agent’s conclusion and its underlying reasoning are independently reliable. Data shown are abbreviated and lightly modified to satisfy privacy requirements. Icons are sourced from Flaticon (https://www.flaticon.com/) under the Flaticon license with attribution. Full source links, license details and modifications are provided in the Third Party Rights Table. spec., specialty groups.

**Extended Data Fig. 1:**
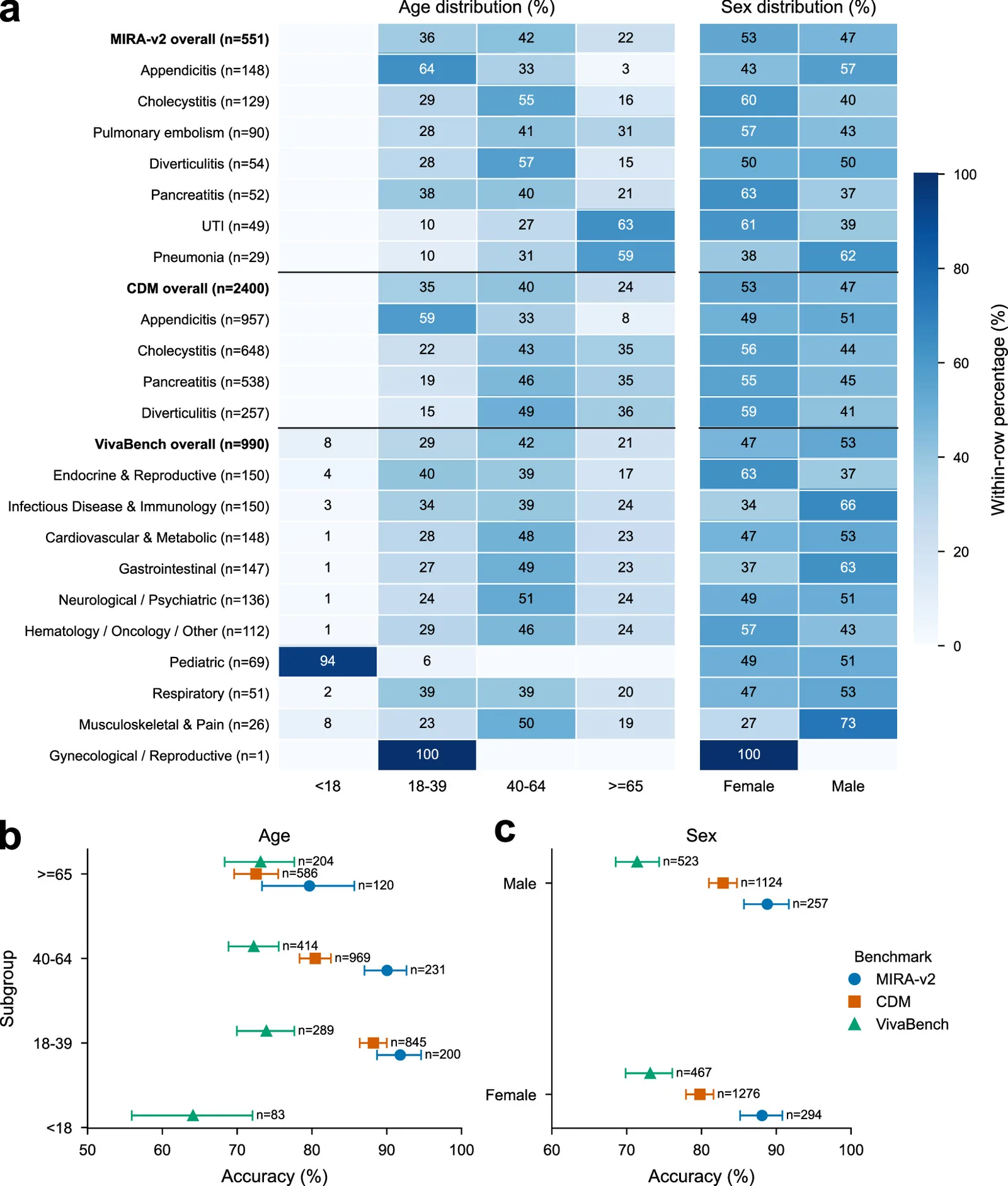
Benchmark demographic composition and subgroup diagnostic accuracy. **a**, Heatmaps showing age-group (left) and sex (right) distributions for each benchmark overall and by disease category or specialty group. Rows indicate benchmark-level or subgroup-level cohorts, with case counts shown in parentheses. Cell values denote within-row percentages. Age and recorded sex were obtained from the source benchmark records and used for descriptive subgroup analyses. Age groups were defined as <18, 18-39, 40-64 and ≥65 years; sex labels were harmonized across datasets. (**b**, **c**) Diagnostic accuracy stratified by age group (**b**) and sex (**c**). Points show mean accuracy across five repeated stochastic model-inference runs, and horizontal bars show case-level bootstrap 95% confidence intervals. Labels report the exact number of unique benchmark cases in each subgroup. In MIRA-v2, accuracy decreased from 91.8% in the 18-39 age group to 79.7% in the ≥65 group. In CDM, accuracy similarly decreased from 88.2% in the 18-39 group to 72.5% in the ≥65 group. In VivaBench, the pediatric subgroup had lower accuracy than the adult subgroups. Sex-stratified performance was comparatively similar in MIRA-v2 and VivaBench, while CDM showed a modest difference between female and male cases. Source data

To establish a baseline for subsequent reliability analyses, we evaluated our fully on-premise dual-agent framework (Fig. 1 and Methods), in which a Physician Agent interacts with a Patient Agent, retrieves benchmark-grounded evidence through tool use and returns a final diagnosis with a reasoning trace. We applied this framework to the two primary MIMIC-IV-derived clinical reasoning benchmarks: MIRA-v2 (ref. ^29^) and Clinical Decision Making (CDM)^30^ (Extended Data Fig. 1a).

**Fig. 2:**
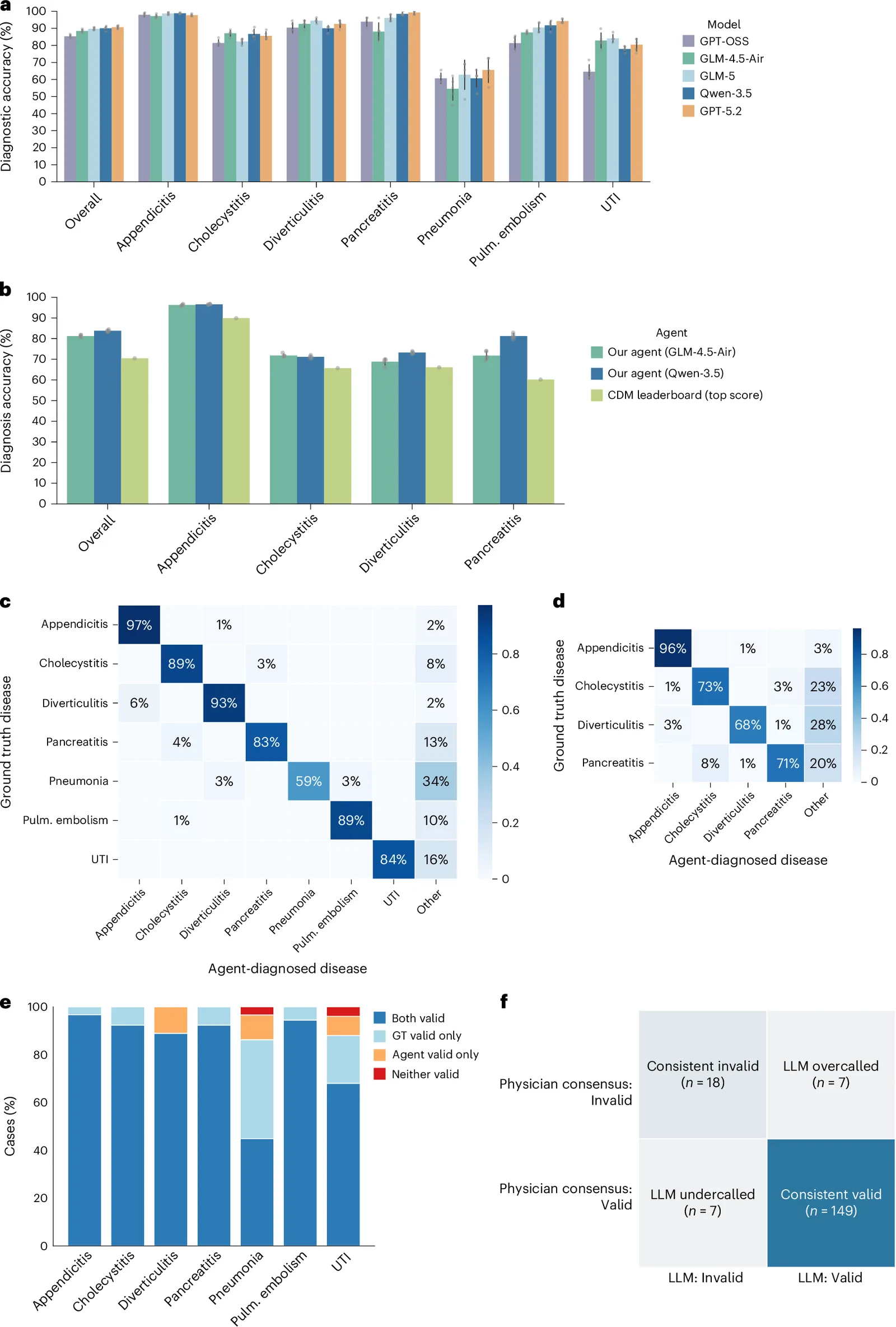
Diagnostic performance of a fully on-premise clinical agent across standardized benchmarks. **a**, Diagnostic accuracy of four on-premise open-weight models (GLM-4.5-Air, GLM-5, Qwen-3.5 and GPT-OSS) and a cloud baseline (GPT-5.2) across seven MIRA-v2 diseases. All systems used the same agent architecture and evaluation pipeline, differing only in the underlying LLM. For each model, accuracy is reported at the best-performing temperature setting; sensitivity to temperature is analyzed in the ‘Implementation sensitivity analyses of behavioral consistency’ section. **b**, Accuracy of the on-premise agents (GLM-4.5-Air and Qwen-3.5) compared to the top reported score on the official CDM leaderboard (Gemma-3) across four disease categories, evaluated using the benchmark’s fuzzy-matching protocol. **c**,**d**, Row-normalized confusion matrices for MIRA-v2 (**c**) and CDM (**d**), with rows denoting ground truth categories and columns denoting agent diagnosis categories. Cell values represent the percentage of cases within each ground truth category assigned to each predicted category. Matrices are shown for the run with accuracy closest to the five-run mean. **e**, Blinded physician adjudication of diagnostic validity (*n* = 181). Bars indicate the proportion of cases judged valid for both the agent diagnosis and the EHR label (‘Both Valid’), valid for the agent diagnosis only (‘Agent Valid Only’), valid for the EHR label only (‘GT Valid Only’) or neither. **f**, Agreement between the automated LLM-based judge and physician consensus on agent diagnosis validity (*n* = 181). Overall raw agreement was 92.3% (167/181; Gwet’s AC1 = 0.898, PABAK = 0.845). For bar plots in **a** and **b**, bars show mean ± s.d. across five independent stochastic inference runs; overlaid points indicate individual runs. The Gemma-3 leaderboard result in **b** is shown as the reported benchmark reference value. GT, ground truth; PABAK, prevalence-adjusted bias-adjusted kappa; Pulm., pulmonary; UTI, urinary tract infection. Source data

On the primary benchmark, MIRA-v2 (seven diagnostic conditions), we evaluated four on-premise open-weight models alongside a cloud baseline (GPT-5.2), using an identical agent architecture and evaluation pipeline that differed only in the underlying LLM (Fig. 2a). Qwen-3.5 achieved the highest on-premise accuracy (90.0%), followed by GLM-5 (89.7%), GLM-4.5-Air (88.4%) and GPT-OSS (85.3%); GPT-5.2 achieved 90.7%. The best on-premise model was, therefore, within 0.7 percentage points of the cloud baseline. Accuracy was lowest for pneumonia across all models; per-disease results for all models are provided in Supplementary Table 1.

We next tested whether this performance extended to a second benchmark. On CDM (four abdominal diagnostic categories; *n* = 2,400), Qwen-3.5 achieved 83.8% accuracy and GLM-4.5-Air achieved 81.2% accuracy under the established evaluation protocol (Fig. 2b and Supplementary Table 1). The highest previously reported open-weight result on this benchmark was 70.5% (Gemma-3)^31^. Accuracy varied little across five independent runs (Fig. 2b).

Across both MIMIC-derived benchmarks, residual errors were concentrated in clinically overlapping categories, including pancreatitis versus cholecystitis and appendicitis versus diverticulitis (Fig. 2c,d). To assess whether some apparent errors reflected benchmark label limitations rather than clinically implausible outputs, we performed blinded physician adjudication on a randomized subset of MIRA-v2 cases (*n* = 181, 33% of the benchmark; Supplementary Table 2). Physician consensus judged 81.8% of reviewed cases clinically valid for both the agent diagnosis and the reference label; in 4.4%, the agent diagnosis was considered clinically valid despite disagreement with the electronic health record (EHR) label (Fig. 2e and Supplementary Table 3). Agreement between the automated LLM-based evaluator and physician consensus was 92.3% overall (167/181; Gwet’s Agreement Coefficient 1 (AC1) = 0.898; Fig. 2f); full inter-rater and per-disease agreement details are provided in the Methods and Supplementary Tables 4 and 5.

**Extended Data Fig. 2:**
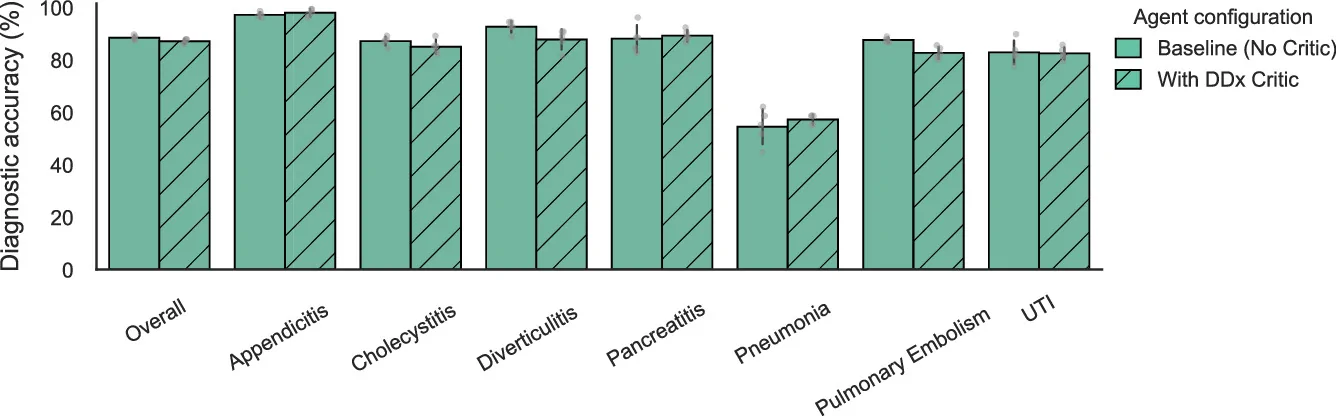
Effect of the DDx Critic Agent on diagnostic accuracy. Mean diagnostic accuracy across five independent stochastic inference runs is shown for the baseline agent and the multi-agent configuration incorporating a DDx Critic. Bars show mean ± s.d.; overlaid points indicate individual runs (n = 5 independent stochastic inference runs per configuration). Overall accuracy was not improved by adding the critic. Source data

We also performed descriptive subgroup analyses stratified by age group and sex across MIRA-v2, CDM and VivaBench (Extended Data Fig. 1) and examined a multi-agent variant incorporating a differential diagnosis (DDx) Critic Agent. The critic did not improve overall diagnostic accuracy relative to the baseline single-agent configuration (87.1% with critic versus 88.4% without; Extended Data Fig. 2), although pneumonia showed a partial exception. Because the critic configuration did not translate into an overall gain, all subsequent confidence and selective-autonomy analyses were performed using the baseline single-agent setup.

### Deconstructing decisional trust with a multi-perspective confidence framework

To evaluate the discriminative performance of the proposed reliability measures, we performed the primary confidence analyses on MIRA-v2 (*n* = 551) under baseline conditions unless stated otherwise. Primary reliability analyses used the GLM-4.5-Air baseline to keep the workflow fixed across experiments. We quantified three classes of reliability-related metrics—internal generation likelihood (ProbScore), expressed language cues (LingCert and ConceptDensity) and behavioral stability across repeated runs (Consistency)—for both the final diagnostic output (Dx) and the accompanying reasoning trace (R).

**Fig. 3:**
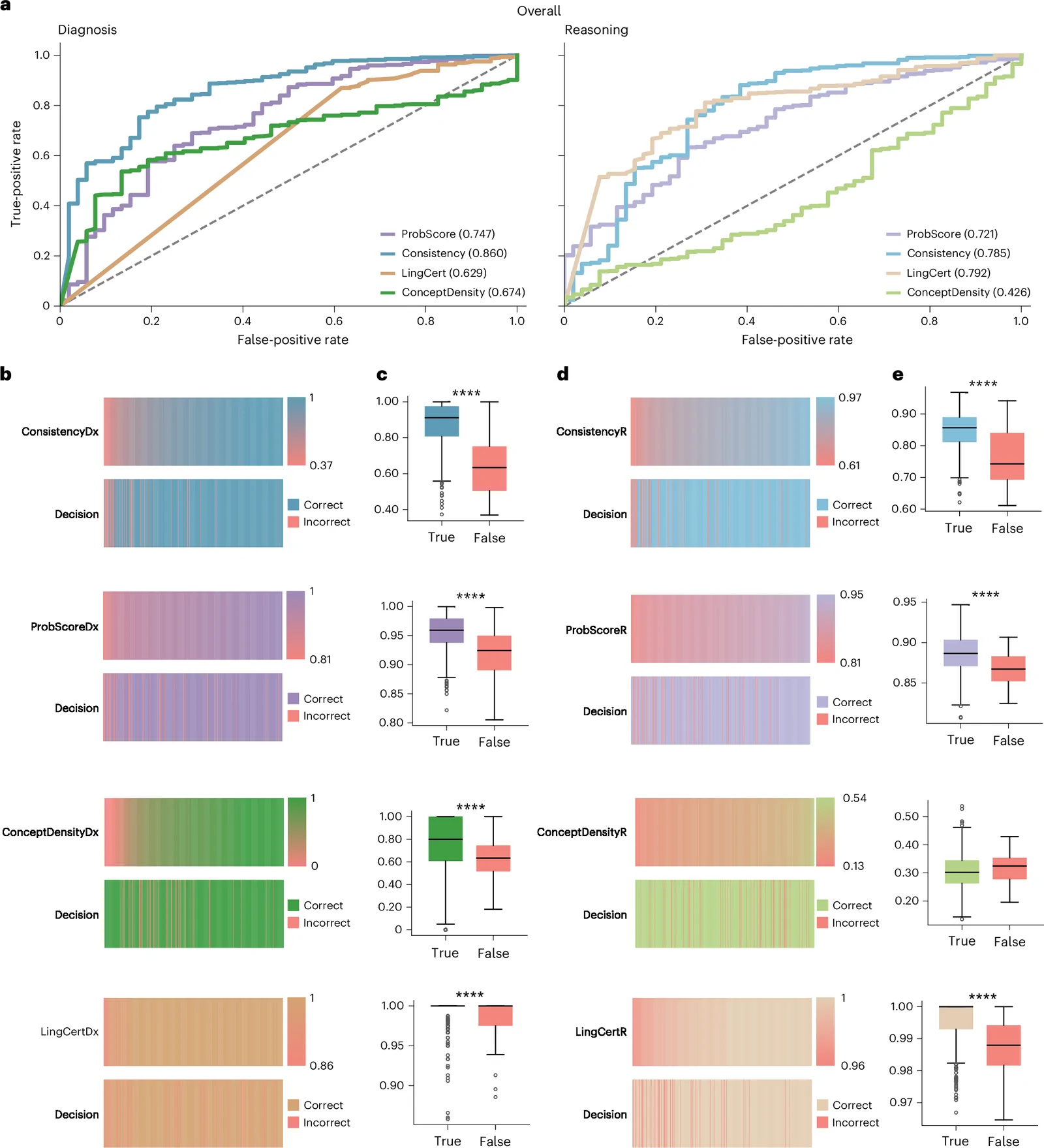
Discriminative power of confidence metrics across diagnostic outcome and reasoning process. **a**, Dual-stream ROC analysis comparing discriminative performance for the diagnostic output (left) and reasoning trace (right). ConsistencyDx showed the highest discrimination for diagnostic correctness (AUC = 0.860). LingCertR showed higher discrimination in the reasoning trace than in the diagnostic output (AUC = 0.792). ConceptDensityR showed an inverse trend with correctness but did not behave as a reliable confidence measure, with higher values associated with incorrect reasoning traces. **b**−**e**, Distributional characteristics of correct and incorrect cases across metrics. Barcode plots (**b**,**d**) show the density of individual cases; box plots (**c**,**e**) summarize group separation. Each observation represents one unique MIRA-v2 benchmark case (*n* = 551; correct, *n* = 499, and incorrect, *n* = 52). Case-level confidence metrics were computed from five repeated stochastic model inferences per case; these computational repeats were not treated as independent observations. Boxes show the median and interquartile range (Q1−Q3); whiskers extend to the most extreme values within 1.5 times the interquartile range; observations beyond the whiskers are shown as open circles. For ConsistencyDx, incorrect cases are concentrated at lower values, whereas some other metrics retain high-valued errors. Two-sided Mann−Whitney *U*-tests with Holm adjustment across the eight prespecified metrics yielded Holm-adjusted *P* values of 2.336 × 10^−8^, 7.459 × 10^−17^, 3.328 × 10^−6^ and 6.469 × 10^−5^ for ProbScoreDx, ConsistencyDx, LingCertDx and ConceptDensityDx, respectively (**c**), and 5.972 × 10^−7^, 8.122 × 10^−11^, 1.887 × 10^−12^ and 0.07955 for ProbScoreR, ConsistencyR, LingCertR and ConceptDensityR, respectively (**e**). Incorrect traces showed a higher median ConceptDensityR than correct traces, but the difference did not reach conventional statistical significance. ****P < 0.0001. Source data

Across metrics, ConsistencyDx provided the strongest discrimination of diagnostic correctness in dual-stream receiver operating characteristic (ROC) analysis (AUC = 0.860) compared to ProbScoreDx (AUC = 0.747) and other metrics (Fig. 3a). The difference between ConsistencyDx and ProbScoreDx was significant by DeLong’s test (difference in AUC = 0.114, *P* = 0.0011; false discovery rate (FDR)-adjusted *P* = 0.0034). Distributional analyses supported this ranking: correct diagnoses clustered near maximal Consistency, whereas errors were enriched at lower values (Fig. 3b,c). Bootstrapped 95% confidence intervals for all AUC estimates are provided in Supplementary Table 6.

**Extended Data Fig. 3:**
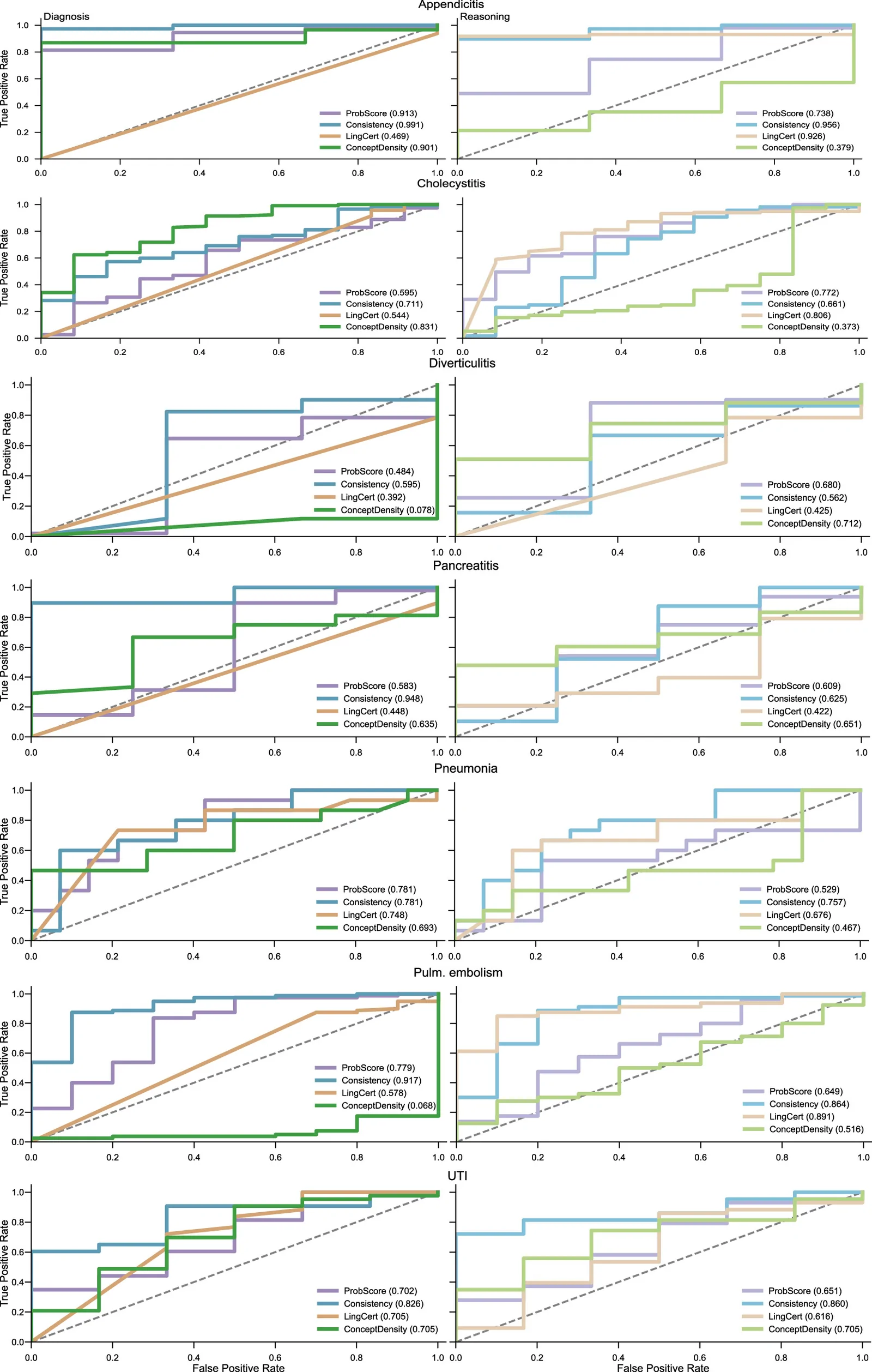
Discriminability of confidence metrics across disease categories. Paired Receiver Operating Characteristic (ROC) curves evaluating the performance of eight confidence metrics across seven distinct clinical diseases. The left column displays metrics derived from the Diagnostic Outcome, while the right column displays metrics derived from the Reasoning Process. In several high-risk or ambiguous categories, LingCertR exceeded internal likelihood as a discriminator (for example, appendicitis, cholecystitis and pulmonary embolism), whereas internal likelihood approached chance or worse for some diagnoses (for example, pancreatitis). Performance varied by clinical condition, indicating that the discriminative value of language-derived and likelihood-based metrics was context dependent. Source data

The remaining metrics showed more limited or stream-specific utility. In the diagnosis stream, ProbScoreDx retained moderate discrimination but exhibited a residual tail of high-valued errors (Fig. 3b). In the reasoning stream, LingCertR slightly exceeded ConsistencyR (AUC = 0.792 versus AUC = 0.785), and incorrect reasoning traces were shifted toward lower linguistic certainty (Fig. 3d,e). ConceptDensityDx retained modest discrimination (AUC = 0.674), whereas ConceptDensityR showed below-chance discrimination (AUC = 0.426) and no significant separation between correct and incorrect cases (Holm-adjusted *P* = 0.08; Fig. 3a,e), indicating that reasoning trace concept density did not behave as a confidence signal. Condition-specific paired ROC curves for all eight metrics are shown in Extended Data Fig. 3.

A supplementary cross-model analysis on Qwen-3.5, GLM-5 and GPT-OSS further showed that this pattern was not unique to GLM-4.5-Air: across all three additional models, ConsistencyDx remained the strongest evaluated diagnostic stream metric (Supplementary Table 34). Together, these results show that behavioral stability was the strongest signal, whereas language-derived metrics contributed complementary information, particularly in the reasoning stream.

### Confidence−state interactions reveal mechanisms of diagnostic reliability

**Fig. 4:**
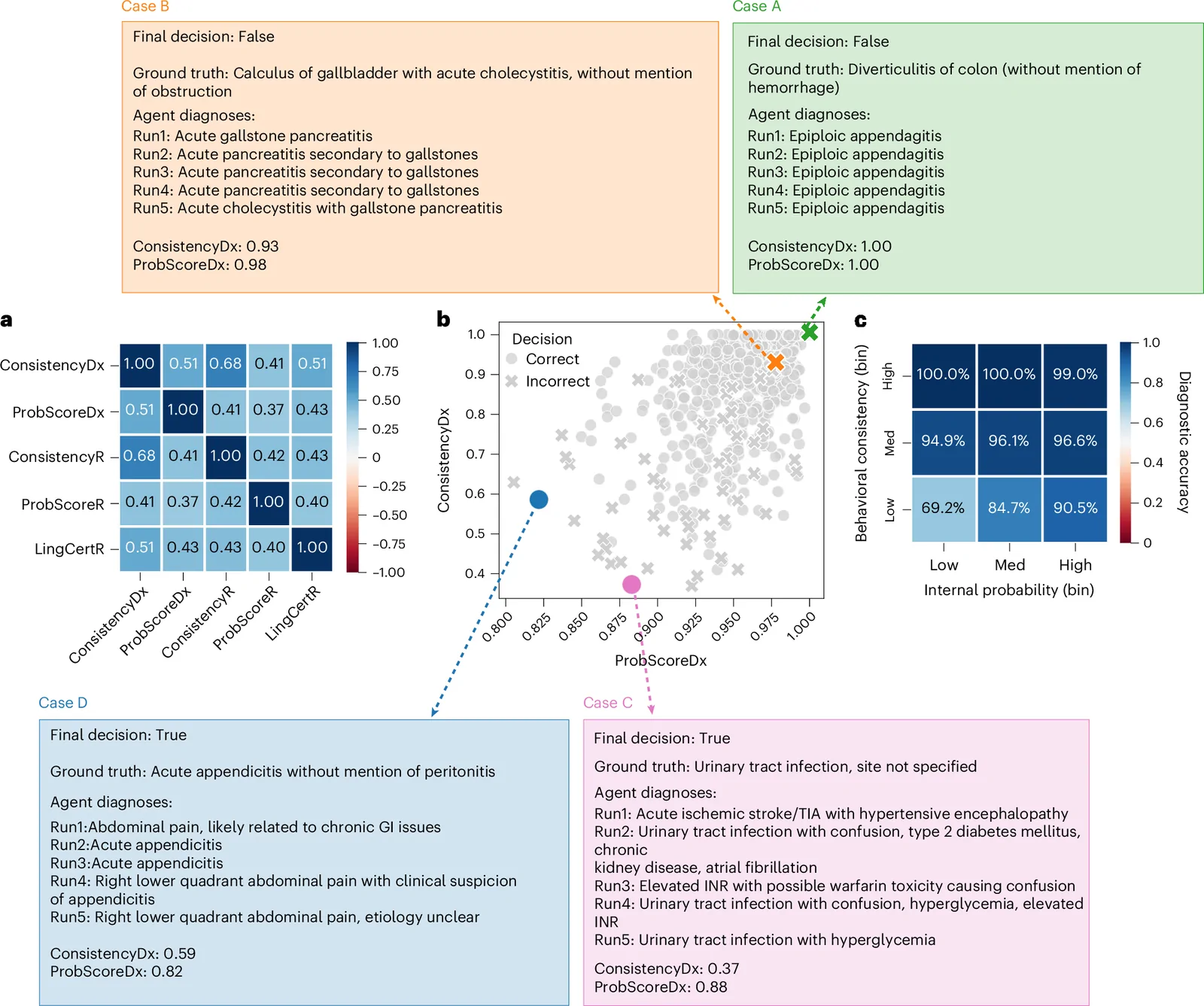
Confidence−state interactions and diagnostic outcomes. **a**, Pearsonʼs correlation matrix for the top-performing reliability-related metrics. Associations were moderate rather than collinear, indicating partial overlap but non-redundancy among diagnostic stability, internal probability and reasoning-level metrics. The strongest correlation was observed between ConsistencyDx and ConsistencyR. **b**, Scatter plot of Internal Probability versus Behavioral Consistency, overlaid with specific case studies. Correct diagnoses (gray dots) cluster in the top-right ‘Safe Zone’; incorrect diagnoses (gray crosses) are dispersed. Colored markers correspond to the illustrative cases in the surrounding panels. **c**, Grid-based accuracy heatmap illustrating the synergistic effect of internal and behavioral metrics. Accuracy heatmap across binned diagnostic internal probability and diagnostic behavioral consistency. Accuracy approached ceiling levels across the high-consistency row (99.0−100.0%), regardless of probability bin, whereas internal probability provided additional stratification primarily when consistency was not high. GI, gastrointestinal; INR, international normalized ratio; TIA, transient ischemic attack. Source data

We next examined how the top-performing reliability-related measures related to one another and to diagnostic outcomes. Correlation analysis showed moderate, non-redundant associations among these metrics (Fig. 4a). In particular, internal probability and diagnostic behavioral consistency were moderately correlated (Pearsonʼs *r* = 0.51), indicating partial overlap without equivalence, whereas the strongest association was observed between ConsistencyDx and ConsistencyR (*r* = 0.68). Variance inflation factors were low for all retained variables (Supplementary Table 7), supporting that these measures captured partially overlapping but non-collinear aspects of model behavior.

We next mapped decisions into a two-dimensional confidence space defined by internal probability and diagnostic behavioral consistency (Fig. 4b,c). Correct diagnoses concentrated in the high-consistency region, whereas discordant states, particularly high internal probability paired with low consistency, contained a disproportionate fraction of errors (Fig. 4b). Quantitatively, accuracy approached ceiling levels across the high-consistency row (99.0−100.0%), regardless of internal probability bin, indicating that behavioral consistency was the dominant determinant of diagnostic accuracy, whereas internal probability provided secondary stratification mainly in less stable states (Fig. 4c).

Case review clarified clinically relevant failure modes (Fig. 4b). Two recurrent categories emerged. First, some outputs were scored as incorrect despite stable, high-confidence behavior, reflecting limitations of administrative labels and single-label evaluation. In case A (ground truth: diverticulitis), the agent consistently diagnosed epiploic appendagitis in line with the radiology report; expert adjudication attributed the discrepancy to the absence of a dedicated International Classification of Diseases, 9th Revision (ICD-9) code for epiploic appendagitis, necessitating a proxy label. In case B (ground truth: acute cholecystitis), the agent prioritized acute pancreatitis in the setting of markedly elevated lipase; pancreatitis was documented as a secondary diagnosis in the full record but was not credited by the single-label protocol. Second, some correct cases showed reduced inference-time confidence signals, consistent with missing evidence or intrinsic clinical ambiguity. In case C (urinary tract infection), urinalysis information was absent, and the agent showed reduced internal probability with diagnoses varying across runs, consistent with reduced reliability under evidentiary scarcity. In case D (early appendicitis with classic symptoms but normal computed tomography), the agent produced the correct diagnosis but with reduced confidence signals (probability and consistency), consistent with calibrated uncertainty in an intrinsically ambiguous presentation.

Together, case review showed that discordant confidence states were enriched for both genuine model uncertainty and benchmark-label limitations, reinforcing that internal likelihood and behavioral stability capture related but non-equivalent aspects of reliability.

### Stress testing decisional trust under induced informational scarcity

**Fig. 5:**
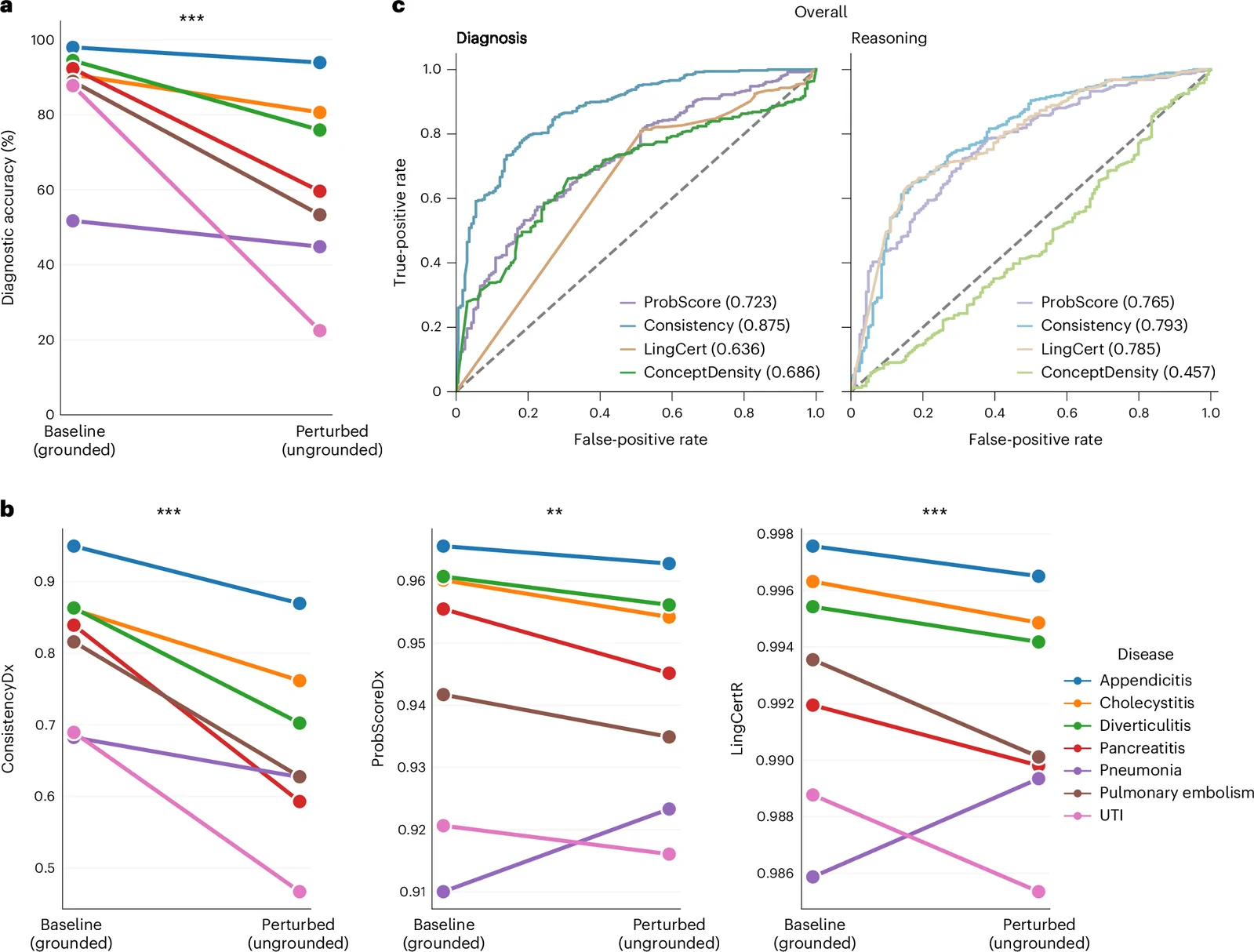
Stress testing decisional trust under induced information scarcity. For **a** and **b**, analyses were performed at the paired case level across the Baseline (grounded) and Perturbed (ungrounded) conditions (*n* = 551 unique benchmark cases). Case-level diagnostic correctness and confidence metrics were computed from repeated stochastic inferences, with five inferences per case except for one Perturbed case with four completed inferences; these were treated as computational repeats rather than independent observations. A single disease legend is shared across **a** and **b**. **a**, Disease-specific diagnostic accuracy under the Perturbed and Baselines conditions; lines connect disease-level mean accuracies. Overall accuracy decreased from 90.6% (499/551) in the Baseline condition to 70.2% (387/551) in the Perturbed condition, a decrease of 20.3 percentage points. A two-sided exact McNemarʼs test across paired cases showed 11 pairs improving and 123 pairs worsening under perturbation (*P* = 4.139 × 10^−25^). No multiple comparison adjustment was applied because this was a single prespecified comparison. **b**, Paired changes in ConsistencyDx, ProbScoreDx and LingCertR between conditions; lines show disease-level means. Two-sided Wilcoxon signed-rank tests with Holm adjustment yielded adjusted *P* = 2.209 × 10^−35^ for ConsistencyDx, 0.007147 for ProbScoreDx and 3.599 × 10^−5^ for LingCertR (*n* = 551 paired cases). ConsistencyDx decreased sharply under perturbation, whereas ProbScoreDx and LingCertR shifted less, and both increased for pneumonia. **c**, Discriminative performance under perturbation (ROC). ConsistencyDx remains strongly discriminative in the Perturbed condition (AUC = 0.875). UTI, urinary tract infection. **P < 0.01, ***P < 0.001. Source data

To assess robustness under reduced evidentiary grounding, we compared the factually grounded baseline with a perturbed setting in which the Patient Agent was ungrounded (initial history withheld) and could generate unreliable testimony (Methods). This perturbation caused a marked decline in diagnostic accuracy across conditions (Fig. 5a).

Confidence signals responded heterogeneously to this performance collapse (Fig. 5b). Consistency decreased sharply in parallel with accuracy, indicating increased decision instability when grounding evidence was removed. By contrast, ProbScore and LingCert showed limited downward shifts and, in some settings, remained high despite lower accuracy. For example, in pneumonia, accuracy declined while both ProbScoreDx and LingCertR increased and remained above 0.90, indicating overconfident outputs under informational scarcity. More broadly, this pattern is inconsistent with a calibrated confidence signal: under degraded evidentiary grounding, some scores remained stable or even increased despite declining diagnostic accuracy, indicating that high absolute score values alone did not reliably track reduced evidentiary support.

Despite this perturbation, discriminative performance (AUC) remained broadly stable relative to baseline (Fig. 5c), indicating that the metrics retained the ability to rank correct versus incorrect decisions. However, their absolute behavior differed. ProbScoreDx and LingCertR retained discrimination while showing limited downward shifts in score, whereas ConsistencyDx preserved discrimination, with a slight increase in AUC (0.875), and shifted downward in absolute value under perturbation. Among the evaluated metrics, ConsistencyDx, therefore, remained the most robust decision-time signal under reduced evidentiary grounding. Bootstrapped 95% confidence intervals for the AUC estimates in Fig. 5c are provided in Supplementary Table 8.

### Enabling selective autonomy

**Fig. 6:**
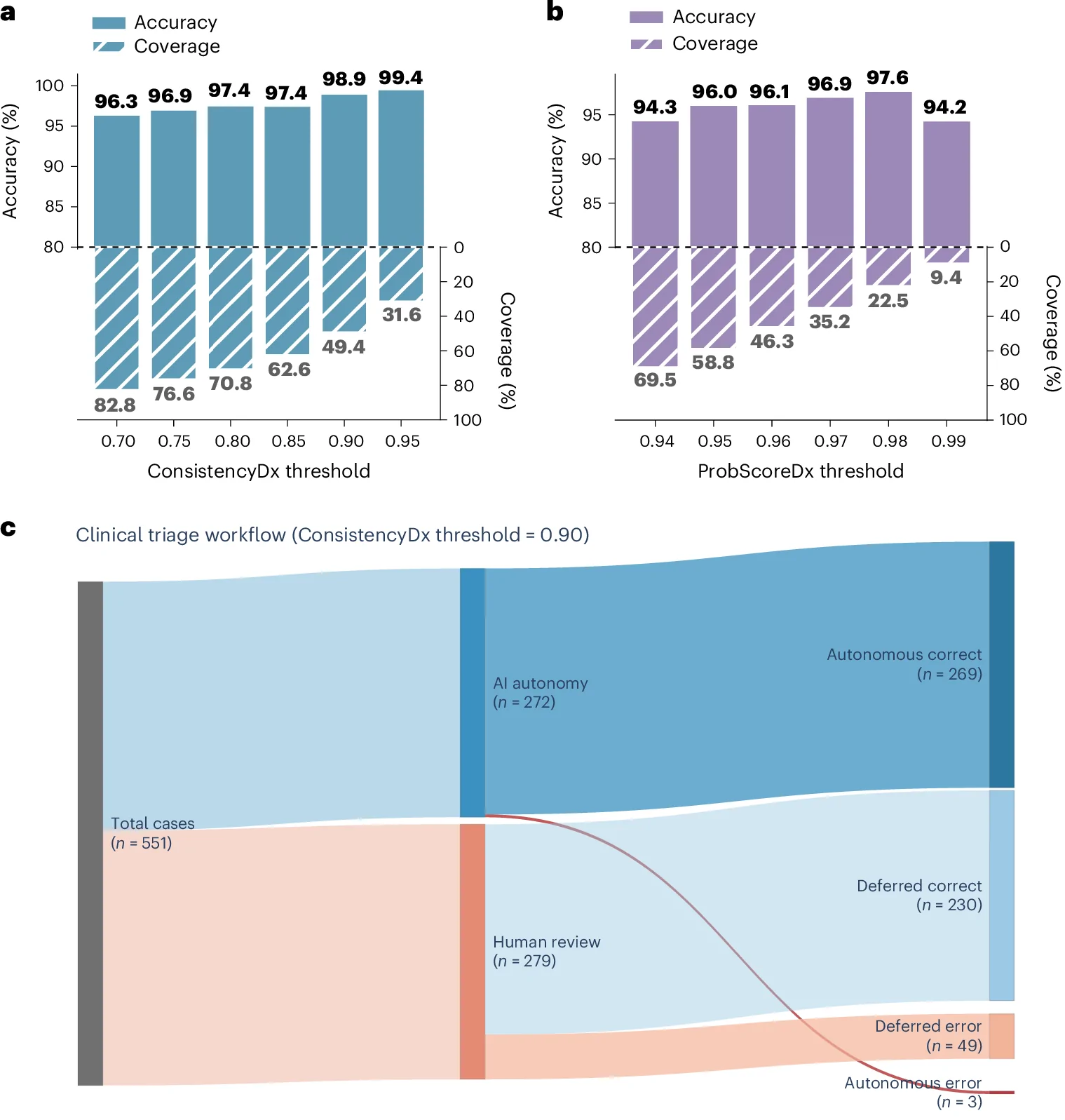
Operationalizing trust through selective autonomy using confidence-based filtering. **a**, Accuracy−coverage tradeoff for gating signal: Behavioral Consistency of the diagnosis (ConsistencyDx). Bars report diagnostic accuracy among cases meeting the threshold (top) and corresponding automation coverage (bottom) across increasing thresholds. ConsistencyDx provides a favorable operating curve: at a threshold of 0.90, accuracy reaches 98.9% while retaining 49.4% of cases. Case-level 95% confidence intervals for coverage and retained-case accuracy are reported in Supplementary Table 9. **b**, Accuracy−coverage tradeoff for internal probability of the diagnosis (ProbScoreDx) as the gating signal. ProbScoreDx did not reach the same retained-case accuracy within the evaluated thresholds; at a threshold of ≥0.98, coverage was 22.5%, and retained-case accuracy was 97.6%. **c**, Simulated triage workflow at a Consistency threshold of 0.90. Flows indicate four outcome categories under confidence-based routing: Autonomous correct (retained for autonomous handling, and the agent’s diagnosis was correct); Autonomous error (retained for autonomous handling, but the agent’s diagnosis was incorrect); Deferred correct (deferred to clinician review because the consistency threshold was not met, despite a correct agent diagnosis); and Deferred error (deferred to clinician review because the consistency threshold was not met, and the agent’s diagnosis was incorrect). Cases meeting the threshold are routed to autonomous handling (*n* = 272; 98.9% accuracy), yielding three autonomous errors. Remaining cases are deferred to human review. The review stream concentrates the majority of errors, supporting the use of Consistency-based gating to defer ambiguous or unstable outputs while reducing clinician workload for high-confidence cases. For visualization, deferred cases are stratified post hoc by reference-label correctness into Deferred error (*n* = 49) and Deferred correct (*n* = 230). Source data

We next asked whether the evaluated gating signals could support a triage protocol in which the agent handled a high-confidence subset autonomously while deferring the remainder to clinician review. We operationalized this using an accuracy−coverage analysis, in which cases were retained only if a confidence score exceeded a threshold; accuracy was computed on retained cases, and coverage was defined as the proportion retained (Fig. 6a,b and Supplementary Table 9).

Across thresholds, ConsistencyDx provided a more favorable operating curve than ProbScoreDx. A ConsistencyDx threshold of 0.90 retained 49.4% of cases (*n* = 272) while achieving 98.9% diagnostic accuracy in the retained subset (Fig. 6a). By contrast, ProbScoreDx did not reach this accuracy level within the evaluated thresholds, illustrating that internal likelihood alone did not provide an equally reliable basis for selective-autonomy routing.

We then simulated a clinical triage workflow at ConsistencyDx ≥ 0.90 (Fig. 6c). Under this policy, the agent handled 272 of 551 cases autonomously with three residual autonomous errors; the deferred stream concentrated the remaining 49 errors for clinician review. Thus, consistency-based gating supported selective autonomy by capturing a large fraction of low-risk decisions while routing unstable outputs to human oversight. A supplementary threshold-sweep analysis showed that residual autonomous-stream errors decreased as review became more conservative but did not fall to zero within the evaluated ConsistencyDx threshold range (Supplementary Table 35). This residual tail was consistent with the high-consistency discordant cases in Fig. 4, indicating that confidence-based routing can reduce but not eliminate benchmark-defined failures. Across additional models, the retained-accuracy/coverage tradeoff for ConsistencyDx was also preserved, although the threshold required to achieve a given balance varied across architectures (Supplementary Table 36).

### External benchmark evaluation on VivaBench

**Extended Data Fig. 4:**
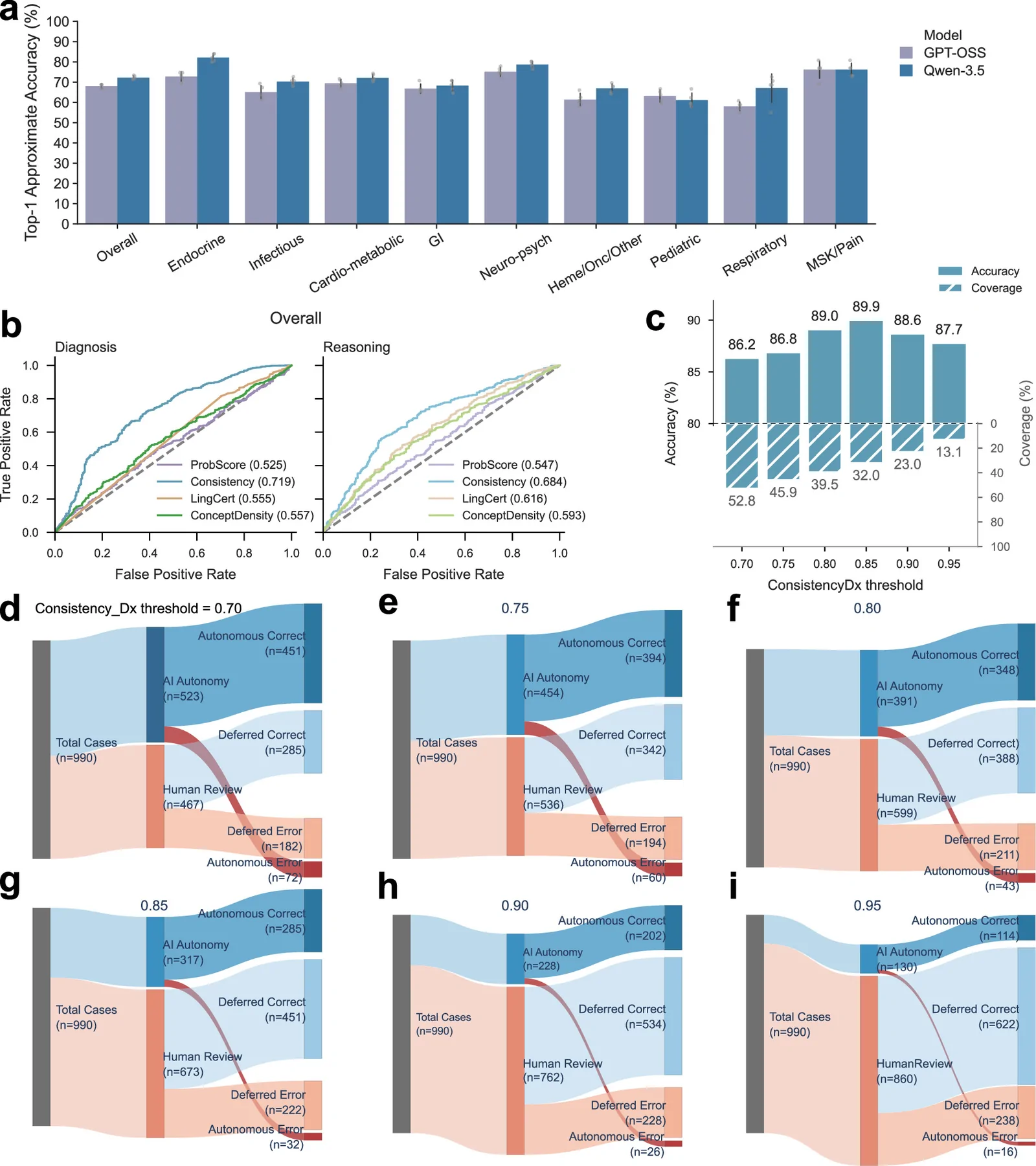
External benchmark evaluation and triage simulation on VivaBench. **a**, Because VivaBench is derived from physician-curated PubMed case reports and was designed to evaluate sequential reasoning under uncertainty, absolute performance is not directly comparable to the MIMIC-derived benchmarks. Top-1 approximate accuracy on the VivaBench benchmark, shown overall and across 10 specialty groups for the evaluated open-weight models. Overall accuracy was 67.96% ± 1.09 for GPT-OSS and 72.22% ± 0.92 for Qwen-3.5. Bars show mean accuracy ± s.d. across five stochastic model-inference runs (n = 5 per model or model-specialty group); overlaid points indicate individual runs. Detailed specialty-group results are provided in Supplementary Table S10. **b**, ROC analysis of reliability-related metrics on VivaBench. ConsistencyDx showed the highest discrimination of correctness among the evaluated metrics (AUC = 0.719), exceeding ConsistencyR (AUC = 0.684), whereas probability-based and linguistic/concept-density metrics showed weaker discrimination. Bootstrapped 95% confidence intervals for all AUC estimates are provided in Supplementary Table S11. **c**, Accuracy–coverage analysis for consistency-based case retention on VivaBench. Accuracy was computed among retained cases, and coverage was defined as the proportion of all cases retained at each ConsistencyDx threshold. Within the evaluated threshold range, the highest retained-set accuracy was observed at a threshold of 0.85 (89.9% accuracy at 32.0% coverage). Full operating points, including 95% confidence intervals, are provided in Supplementary Table S12. **d–i**, Selective-autonomy simulations on VivaBench across increasing ConsistencyDx thresholds (0.70, 0.75, 0.80, 0.85, 0.90 and 0.95). Cases meeting the threshold were routed to AI autonomy and the remaining cases to human review. Flows show autonomous correct, deferred correct, deferred error, autonomous error, illustrating the trade-off between automation coverage and residual risk as the threshold becomes more stringent. Source data

To evaluate portability beyond the MIMIC-derived benchmarks, we tested the on-premise agent on the PubMed-derived VivaBench benchmark, which spans 10 specialty groups and is designed to probe diagnostically challenging sequential reasoning under uncertainty^32^. Because VivaBench is based on physician-curated case reports and provides both a diagnosis list and accepted differentials, performance was evaluated using a benchmark-aligned top-1 approximate accuracy criterion (Methods). As expected for this more demanding external setting, absolute performance was lower than on the MIMIC-derived benchmarks. Among the evaluated open-weight models, Qwen-3.5 achieved higher overall top-1 approximate accuracy than GPT-OSS (72.22 ± 0.92% versus 67.96 ± 1.09%; Extended Data Fig. 4a and Supplementary Table 10).

We next asked whether the main confidence patterns and selective-autonomy behavior observed on MIRA-v2 were preserved externally. On VivaBench, ConsistencyDx again provided the strongest discrimination of correctness among the evaluated metrics (AUC = 0.719), whereas probability-based and linguistic/concept-density metrics showed weaker discrimination (Extended Data Fig. 4b and Supplementary Table 11). Consistency-based case retention again produced a graded tradeoff between retained-set accuracy and coverage (Extended Data Fig. 4c and Supplementary Table 12). Within the evaluated threshold range, the highest retained-set accuracy was observed at ConsistencyDx ≥ 0.85, yielding 89.9% accuracy at 32.0% coverage. Triage simulations across increasing thresholds showed the same qualitative pattern as in MIRA-v2: stricter thresholds reduced automation coverage while progressively lowering residual autonomous-stream errors (Extended Data Fig. 4d–i). These results indicate that the selective-autonomy framework remained portable to a broader external benchmark setting, although overall performance remained heterogeneous across domains.

We next reviewed representative autonomous-stream errors that persisted at higher ConsistencyDx thresholds, asking whether they reflected true misreasoning or disagreement between benchmark endpoints and the clinically appropriate framing at initial presentation (Supplementary Table 13). Two recurrent patterns emerged. First, several cases reflected a timepoint mismatch: the agent prioritized the acute syndrome or admission-relevant problem at presentation, whereas the benchmark endpoint corresponded to a diagnosis established only after later investigations or inpatient evolution. Examples included acute pericarditis versus later-confirmed tuberculosis, ST-elevation myocardial infarction (STEMI) versus Takotsubo syndrome and supracondylar humeral fracture versus reset osmostat. In these cases, physician review suggested that the model’s output was more consistent with the presenting complaint and standard early clinical reasoning than with the later benchmark endpoint.

Second, some cases represented stable but incorrect reasoning, in which the agent converged on a clinically plausible but ultimately incorrect diagnosis by anchoring on a familiar syndrome narrative without prioritizing the key disambiguating evidence. Examples included multiple myeloma rather than primary hyperparathyroidism in a hypercalcemic syndrome case and POEMS syndrome rather than metallosis. More broadly, these high-consistency failures reflected premature closure on a coherent, familiar diagnostic explanation rather than arbitrary outputs. One notable example was a case in which the model converged on thyrotoxic periodic paralysis, whereas physician review noted that the benchmark diagnosis depended on concealed self-induced beta-agonist exposure suggestive of factitious disorder. Such cues would be difficult even for human clinicians to recover when the patient is explicitly hiding the diagnosis and are especially challenging for a conversational agent lacking non-verbal observation. Clinical severity was heterogeneous: some were genuinely concerning high-confidence failures with potential for harmful misdirection, whereas others reflected benchmark-labeling disagreement rather than unsafe reasoning.

### Implementation sensitivity analyses of behavioral consistency

**Extended Data Fig. 5:**
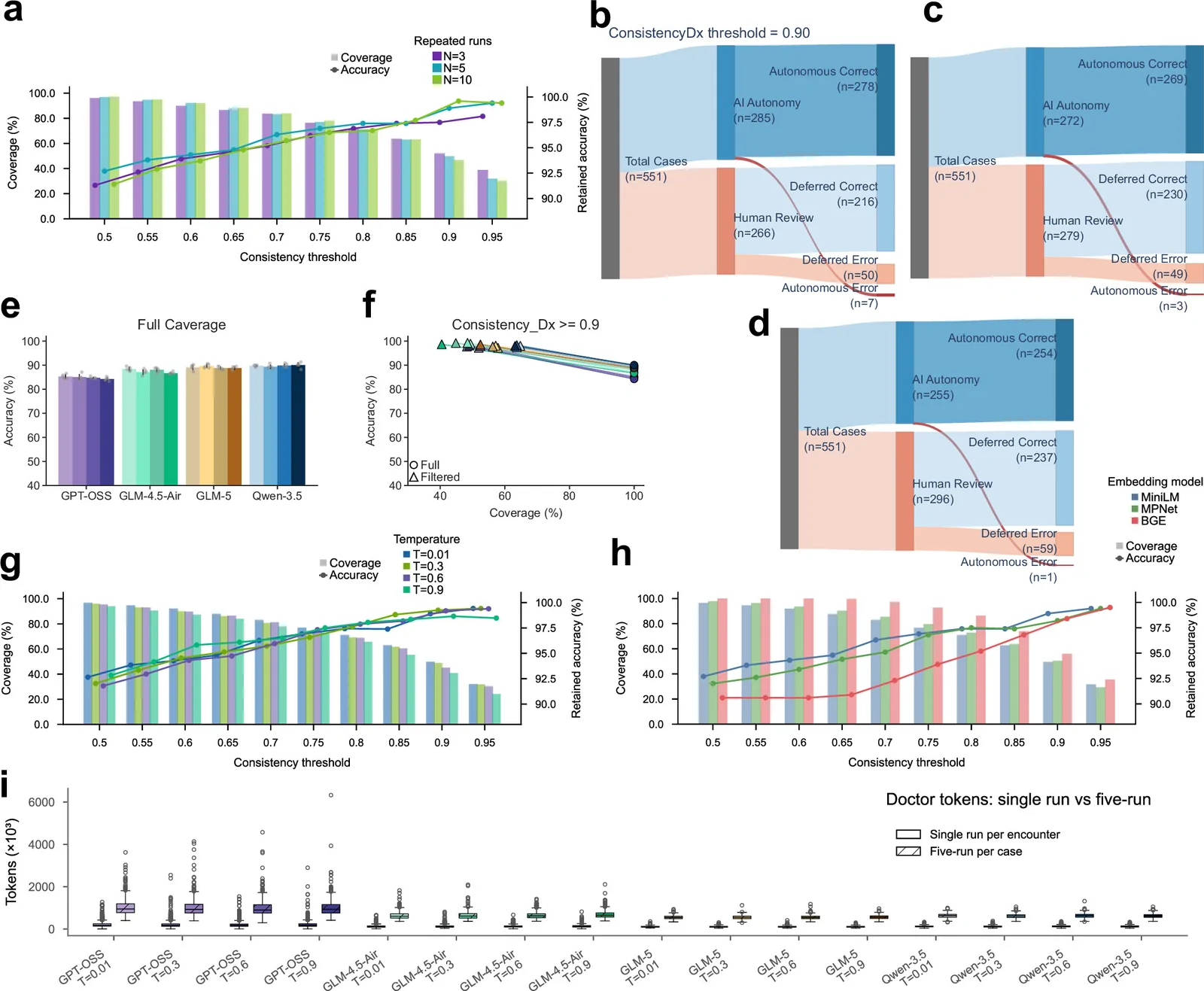
Implementation sensitivity analyses of behavioral consistency. Across panels, coverage denotes the proportion of benchmark cases retained above a specified ConsistencyDx threshold, and retained accuracy denotes diagnostic accuracy among retained cases. Unless otherwise stated, analyses used 551 unique MIRA-v2 benchmark cases. **a**, Coverage and retained accuracy across ConsistencyDx thresholds calculated from N = 3, N=5 or N = 10 repeated stochastic inference runs per case. Exact operating characteristics and 95% Wilson confidence intervals are provided in Supplementary Table S14; paired comparisons are reported in Supplementary Table S15. **b–d**, Representative clinical triage workflows at a ConsistencyDx threshold of 0.90 for N = 3, N = 5, and N = 10, respectively. **e**, Diagnostic accuracy at full coverage across model–temperature configurations. Bars show mean ± s.d. across five computationally repeated stochastic inference runs, and points denote individual runs (n = 5 runs per configuration; 551 cases per run). **f**, Coverage–accuracy operating points at full coverage and after retention at ConsistencyDx≥0.90 across models and decoding temperatures. **g**, Threshold-wise coverage and retained accuracy for GLM-4.5-Air at temperatures 0.01, 0.3, 0.6 and 0.9. Exact values and paired comparisons are provided in Supplementary Tables S16 and S17. **h**, Threshold-wise coverage and retained accuracy using MiniLM, MPNet and BGE embeddings. Exact values and paired comparisons are provided in Supplementary Tables S18 and S19. **i**, Doctor-agent token use for single-run and five-run consistency inference across model–temperature configurations. For single-run inference, each observation is one run-level encounter record (n = 2,755 per configuration, except GLM-5 at T = 0.3, n = 2,754, and Qwen-3.5 at T = 0.9, n = 2,753, owing to missing token-usage metadata). For five-run consistency inference, each observation is the case-level sum of doctor-agent tokens across all available stochastic inference runs for the same case (nominally five runs; n = 551 case-level sums per configuration). Boxes show the interquartile range, centre lines show medians, whiskers extend to the most extreme values within 1.5× the interquartile range, and more extreme observations are shown as open circles. Repeated inferences were computational repeats and were not treated as independent case-level observations. Source data

Unless otherwise stated, ConsistencyDx was estimated from five independent stochastic runs per case using a MiniLM sentence encoder. We examined sensitivity to repeated-run count, decoding temperature and embedding model choice (Extended Data Fig. 5a–h and Supplementary Tables 14–19). Across these analyses, the qualitative behavior of ConsistencyDx was preserved: higher thresholds generally increased retained-set accuracy at the cost of lower coverage, and ConsistencyDx remained informative for routing. Repeated-run count had modest effects on the cohort-level score distribution, but higher thresholds produced progressively more selective triage behavior. For example, at ConsistencyDx ≥ 0.90, retained accuracy increased from 97.5% (*N* = 3) to 98.9% (*N* = 5) and 99.6% (*N* = 10), whereas coverage decreased from 51.7% to 49.4% and 46.3%, respectively. At the same threshold, residual autonomous-stream errors also decreased from seven (*N* = 3) to three (*N* = 5) and one (*N* = 10). Decoding temperature also shifted the operating point of consistency-based triage, while embedding model choice had the largest effect on threshold calibration. At ConsistencyDx ≥0.85, for example, BGE retained 73.9% of cases compared to 62.6% for MiniLM and 63.7% for MPNet, without a corresponding retained-accuracy advantage. These findings indicate that absolute consistency thresholds are not universal and must be calibrated to the deployment configuration rather than transferred directly across settings.

Because consistency estimation requires repeated multi-turn encounters, we also quantified computational cost (Extended Data Fig. 5i and Supplementary Tables 20–23). Aggregating five runs increased doctor agent token use by approximately five-fold relative to single-pass inference, close to the expected linear scaling from repeated execution. Although the runs are, in principle, parallelizable, total token consumption and GPU compute remained substantially higher, and serial wall clock latency would increase correspondingly. Repeated-run consistency is, therefore, a useful but computationally costly decision-time reliability signal whose deployment value must be balanced against infrastructure constraints.

## Discussion

Clinical deployment of autonomous LLM agents depends not only on diagnostic accuracy but also on institutionally governed operation and reliable decision-time assessment of when outputs should be trusted^6,16–19^. In the present study, we addressed these requirements jointly by combining an on-premise agent architecture with inference-time reliability signals that support selective autonomy. Under a matched agent architecture and evaluation pipeline, on-premise performance on MIRA-v2 remained close to the cloud baseline, indicating that governance-oriented local deployment did not impose a large performance penalty in this setting. The main contribution, however, is not competitive performance alone but also the demonstration that reliability signals can be quantified at decision time and translated into explicit triage policies within a locally governed workflow^5,16–19^.

A central finding was that behavioral consistency provided the strongest indicator of diagnostic correctness. Cross-run semantic consistency of the final diagnostic output outperformed token-level likelihood and remained informative under induced information scarcity, where accuracy deteriorated but consistency both preserved discrimination and shifted downward in absolute value. By contrast, internal likelihood retained only partial utility and did not reliably decrease when evidence was removed, helping to explain why probability-based gating was less effective for selective autonomy. In this study, cross-run consistency was used not as a decoding rule but as a decision-time reliability signal for autonomous clinical workflows.

Our dual-stream evaluation further indicates that reliability depends on what is being scored. Linguistic hedging carried more information in the reasoning trace than in the final diagnostic label, consistent with uncertainty being more likely to appear in extended explanatory language than in a brief final diagnosis. This measure remained surface level and did not resolve the scope of uncertainty within a sentence, limiting fine-grained clinical interpretability. By contrast, concept density in reasoning did not behave as a confidence measure and, instead, showed an inverse trend with correctness, raising the possibility that jargon-rich rationales may project competence without corresponding factual grounding. However, post hoc analyses suggested that reasoning trace concept density covaried with aspects of the diagnostic workup rather than independently tracking incorrectness, reinforcing that it should not be interpreted as a conventional confidence measure (Supplementary Fig. 1). These findings argue for treating internal, expressed and behavioral signals as related but non-interchangeable views of reliability. A recent calibration probing study^33^ also decomposes confidence into intrinsic, consistency-based and semantic components and similarly finds that inconsistency-aware signals can outperform intrinsic probability alone, although the task setting and operationalization differ substantially from ours. For transparency, we also report a supplementary semantic-entropy-inspired comparison (SemanticCertaintyDx) on MIRA-v2; this metric showed strong discrimination but remained highly correlated with ConsistencyDx, which retained superior performance (Supplementary Table 37)^26^.

These signals can be operationalized for selective autonomy. Consistency-based gating retained a substantially larger autonomous subset than probability-based gating at similar retained-set accuracy, allowing lower-risk cases to be handled autonomously while concentrating unstable cases into the review stream. Selective autonomy, therefore, does not remove uncertainty but redistributes it. In an on-premise deployment, thresholds, escalation rules and validation procedures can remain under institutional governance rather than being delegated externally. At the same time, confidence-based routing is a mechanism for risk stratification, not a substitute for continuing clinical governance. The threshold-sweep analysis reinforces this: more conservative review policies reduced residual autonomous-stream errors but did not eliminate them in the benchmark setting. Some of these residual high-consistency errors also reflected benchmark-label limitations rather than uniformly unsafe reasoning, suggesting that zero benchmark discordance is neither a realistic nor a sufficient deployment target.

An additional deployment-relevant finding was the age gradient observed in the subgroup analyses. On both MIRA-v2 and CDM, diagnostic accuracy was lower in older age groups than in younger adults, whereas sex-stratified differences were smaller. This matters for selective autonomy because a routing concerns not only average retained accuracy but also which patients are more likely to be handled autonomously versus deferred. Exploratory age-stratified analyses were directionally consistent with this concern under a fixed consistency threshold (Supplementary Table 38). If such reductions affect older patients, they could map onto a population already carrying higher clinical risk, with implications for both safety and fairness. These analyses remain descriptive: without counterfactual demographic perturbation or prospective workflow evaluation, we cannot determine whether the observed gradients arise from benchmark composition, case complexity, documentation effects or model bias. We, therefore, interpret this as an important deployment consideration requiring dedicated bias auditing rather than as a resolved finding.

The external VivaBench analysis provides partial support for the portability of this framework beyond the MIMIC-derived setting. On this independent PubMed-derived benchmark, ConsistencyDx again emerged as the strongest evaluated signal, and threshold-based gating again produced a graded tradeoff between retained-set accuracy and coverage. Because VivaBench is assembled from physician-curated PubMed case reports and designed to evaluate sequential reasoning under uncertainty, its absolute performance should not be interpreted as directly comparable to the MIMIC-derived tasks but, rather, as a more demanding external reasoning setting. Physician review showed that these residual errors were heterogeneous, reflecting either a timepoint mismatch between presentation-level reasoning and a later etiologic benchmark endpoint or stable but incorrect reasoning in which the model anchored on a familiar syndrome pattern without prioritizing decisive disambiguating evidence (Results). Some were further amplified by the limits of a conversational setting, where key cues were concealed or depended on non-verbal observation. This distinction is clinically relevant: in first-contact settings, the task is often to identify the high-risk syndrome and avoid unsafe dismissal rather than to infer immediately the final etiologic diagnosis that may become apparent only after later investigations or clinical evolution. Benchmark disagreement should, therefore, not automatically be interpreted as equivalent to unsafe reasoning, although this does not diminish the importance of the truly high-confidence failures that remained.

Computational profiling highlights a practical tradeoff of consistency-based autonomy. Repeated-run consistency estimation increased token use by approximately five-fold relative to single-pass inference. Although the repeated runs are independent and, therefore, amenable to parallel execution, total token consumption and GPU compute remain substantially higher. Repeated-run consistency should, therefore, be understood as a useful but computationally costly decision-time reliability signal whose deployment value must be balanced against infrastructure constraints.

Several limitations should be acknowledged. First, both primary benchmarks derive from MIMIC-IV, reflecting a single-institution data ecology; VivaBench broadens the evaluation setting but does not substitute for validation on independent datasets or in prospective workflows. Second, the evaluation is limited to text-based diagnostic reasoning; although the framework is modular and could incorporate multimodal components, native image interpretation represents a distinct clinical and methodological subproblem rather than a simple extension of the workflow studied here. Third, a multi-agent critic extension showed heterogeneous condition-specific effects without improving overall performance, leaving the circumstances under which critique is beneficial undefined. Fourth, implementation analyses showed that absolute consistency thresholds were not universal: decoding temperature, semantic encoder choice and repeated-run count altered operating characteristics, indicating that thresholds must be calibrated to the deployment configuration. Finally, all evaluations were retrospective simulations; prospective studies and dedicated bias audits will be required to determine how these signals affect clinician reliance, review burden, safety outcomes and fairness across patient subgroups in real practice.

In summary, our findings support a practical framework for more trustworthy medical AI agents in which institutionally governed deployment is paired with decision-time reliability estimation to support selective autonomy with explicit human escalation. Behavioral consistency was the most informative signal in this setting, not because it eliminates uncertainty but because it helps determine when autonomous handling may be reasonable and when uncertainty should remain with the clinician.

## Methods

### Ethics statement

This study utilized deidentified, retrospective clinical data from the MIMIC-IV (version 2.2) and physician-curated cases from previously published reports in VivaBench. The collection of patient information and creation of the MIMIC-IV resource was reviewed and approved by the institutional review boards of the Beth Israel Deaconess Medical Center (BIDMC) and the Massachusetts Institute of Technology (MIT), which granted a waiver of informed consent. No participants were prospectively recruited or compensated for the present study, and no additional informed consent was obtained. All data processing was conducted within a fully on-premise, institutionally governed environment. No protected health information or deidentified clinical text was transmitted to, stored by or accessible to any external entities or model providers. All researchers involved in data analysis of this study completed the required CITI Program training (‘Data or Specimens Only Research’) and adhered strictly to the PhysioNet Credentialed Health Data Use Agreement.

### Dataset

To evaluate the agent’s clinical reasoning capabilities across distinct diagnostic settings and to assess generalizability, we used three benchmarks spanning two independent data sources (Fig. 1). Two benchmarks are derived from MIMIC-IV, a publicly available dataset of deidentified EHRs from BIDMC: MIRA-v2 (*n* = 551; seven conditions), adapted from the MIRA^29^ framework, which serves as the primary benchmark for diagnostic decision-making, and CDM^30^ (*n* = 2,400; four acute abdominal conditions), which serves as a cross-validation benchmark for diagnostic reasoning at scale. A third benchmark, VivaBench^32^ (*n* = 990; 10 clinical specialty groups), provides independent external validation across a broad multi-specialty case mix. Each benchmark is described in detail in Extended Data Fig. 1a.

The primary benchmark (MIRA-v2) includes seven conditions, including appendicitis, cholecystitis, diverticulitis, pancreatitis, pneumonia, pulmonary embolism and urinary tract infection. It simulates early emergency assessment. We excluded the pancreatic cancer subset (*n* = 23) from the original MIRA benchmark for three reasons. First, evaluation of pancreatic masses is inherently multimodal and depends heavily on cross-sectional imaging (computed tomography/magnetic resonance imaging) for diagnosis and staging; because our framework operates on structured text without imaging inputs, including these cases would introduce a modality mismatch that could confound performance and reliability analyses. Second, these encounters more often reflect oncological workup, staging and treatment planning rather than the acute, early-presentation diagnostic reasoning emphasized in this study, shifting the primary decision target from de novo diagnosis toward management decisions. Third, the small sample size limits statistical power and would yield unstable estimates at the disease level.

The cross-validation benchmark (CDM) spans appendicitis, cholecystitis, diverticulitis and pancreatitis, with each case simulating a complete clinical encounter beginning with abdominal pain.

To extend the evaluation beyond the acute-care, single-institution setting represented by the MIMIC-IV-derived benchmarks, we additionally assessed the agent on VivaBench, an independent PubMed-derived diagnostic benchmark (*n* = 990). VivaBench consists of physician-curated cases drawn from published case reports and spans 10 specialty groups, including cardiovascular, endocrine, gastrointestinal, infectious disease, neurological, hematology/oncology, pediatric and respiratory presentations. Unlike the MIMIC-IV-derived benchmarks, VivaBench is designed to simulate viva-style diagnostic reasoning under uncertainty across broader clinical domains rather than admission-specific emergency encounters. This makes it structurally distinct from the more common-condition, EHR-derived MIMIC benchmarks and less directly comparable in absolute performance.

### Agent framework architecture

#### Overview

We developed a fully on-premise, dual-agent simulation framework to evaluate clinical reasoning in autonomous medical agents. The architecture is adapted from the MIRA^29^ framework and centers on two interacting agents: a Physician Agent and a Patient Agent operating within a controlled local environment. Our implementation prioritizes modularity, observability and operational control and supports future multi-agent extensions. Interoperability and EHR integration standards (for example, FHIR^34^) were not modeled, as the present study focuses on diagnostic reasoning and confidence signaling rather than deployment integration.

#### The Patient Agent

The Patient Agent simulates a real patient within the clinical encounter. It is a non-tool-using conversational model restricted to case-specific information provided at initialization, including the chief complaint, a clinical history summary and pre-admission medications when available. For the MIMIC-IV-derived benchmarks, these inputs were derived from the source clinical record; for VivaBench, they were derived from the benchmark-provided case material. Benchmark-specific Patient Agent prompts are provided in Supplementary Tables 25 and 26. Across benchmarks, the Patient Agent was required to remain grounded in the provided case context and to avoid introducing unsupported symptoms, findings, diagnoses or treatments in the baseline setting. Pre-admission medication lists were included for MIRA-v2 when available and omitted for CDM because of data availability.

#### The Physician Agent

The Physician Agent performs diagnostic reasoning through multi-turn dialogue and tool use, using an on-premise LLM. It can request objective clinical evidence via function calling, including laboratory values, urinalysis, radiology reports, microbiology results and physical examination findings (Extended Data Table 1). Tool outputs are returned as textual observations and appended to the agent’s context for subsequent reasoning. Each episode terminates when the agent invokes a submission action (‘admission’), providing (1) a single diagnosis (Dx) and (2) a structured reasoning trace (R). These two outputs are used to compute the reliability-related metrics described in the ‘Multi-perspective confidence framework’ section. Benchmark-specific Physician Agent prompts are provided in Supplementary Tables 27 and 28.

#### Planning strategy

Given the context budget constraints of on-premise models, we removed the explicit Plan tool used in the original MIRA framework to reduce prompt overhead and preserve token capacity for dialogue history and tool outputs. The Physician Agent instead performed implicit planning, in which successive tool calls and intermediate reasoning adaptively guided the diagnostic trajectory across turns.

#### Clinical encounter workflow

Each simulation models a complete clinical encounter from initial presentation to final diagnosis. The encounter opens with the Patient Agent’s initial complaint, instantiated from the case’s chief complaint in the benchmark-specific case material. This opening utterance serves as the first input to the Physician Agent and initiates the diagnostic dialogue.

The core of the encounter is a dynamic, multi-turn interaction. During each encounter, the Physician Agent elicits subjective information from the Patient Agent and retrieves objective findings through the available clinical tools (Extended Data Table 1). Tool outputs are appended to the Physician Agent’s context. The encounter terminates when the Physician Agent invokes the admission tool or reaches the 10-turn limit.

#### Model selection under on-premise constraints

We screened open-weight LLMs for reliable multi-turn dialogue, structured tool calling and sufficient context length. GLM-4.5-Air^35^ was selected as the primary model after several candidates were excluded because of malformed tool calls, unsupported serving infrastructure or context-length limitations. During revision, GPT-OSS^36^, GLM-5 (ref. ^37^) and Qwen-3.5 (ref. ^38^) were additionally evaluated. Detailed screening criteria and failure modes are reported in Supplementary Table 24.

#### Computational cost and resource profiling

We quantified computational cost using token usage and wall clock runtime. Token usage was extracted from run metadata for each completed encounter. Doctor agent token use was analyzed separately from total workflow token use, where total workflow tokens included doctor agent, patient simulator and other workflow component tokens.

For single-run inference, token use was analyzed at the encounter level. For five-run consistency analysis, token use was aggregated at the case level by summing token use across the five repeated encounters for the same case and model temperature configuration. Token use was summarized using median and interquartile range. Five-run overhead ratios were calculated within each model temperature configuration as the median five-run case-level token use divided by the median single-run encounter-level token use.

Wall clock runtime was assessed separately in a controlled latency subset of 35 encounters, consisting of five randomly sampled cases from each of seven disease cohorts. The same subset was used for all four locally hosted model configurations. For each encounter, wall clock time was recorded from simulation start to completion and represents encounter-level runtime, including the doctor agent, patient simulator, tool calls and workflow overhead. Five-run runtime was estimated under a serial execution assumption as five times the measured single-run runtime.

The LLMs were served using vLLM^39^ on NVIDIA H200 and NVIDIA RTX PRO 6000 GPUs. Model-specific details are in Extended Data Table 2. The system was built using the OpenAI Agents SDK^40^ to support flexible orchestration of tool use and conversation flow.

### Experimental configurations

#### Single-agent baseline

Unless otherwise stated, the principal confidence, stress test and selective-autonomy analyses were conducted on MIRA-v2 using a single-agent baseline in which one Physician Agent interacted with one Patient Agent. This baseline used GLM-4.5-Air with *T* = 0.01, top_*p* = 1.0 and top_*k* = 0, where *T*, top_*p* and top_*k* denote sampling temperature, nucleus-sampling probability threshold and top-k sampling cutoff, respectively. We used this configuration for the main reliability analyses to keep the workflow fixed across experiments.

For benchmark−performance comparisons, we evaluated additional on-premise open-weight models under the same agent architecture and evaluation pipeline. On MIRA-v2, these included GLM-4.5-Air, GLM-5, Qwen-3.5 and GPT-OSS; each model is reported at its best-performing temperature setting. On CDM, the main on-premise evaluations were performed with GLM-4.5-Air and Qwen-3.5. Confusion matrix and adjudication analyses in Fig. 2c–f used the GLM-4.5-Air baseline.

#### Multi-agent extension

To explore potential safety enhancements, we implemented a multi-agent system by introducing a DDx Critic Agent. This agent, powered by a domain-specific LLM (MedGemma), serves as a secondary reviewer. It receives the primary agent’s differential diagnosis and returns a structured critique that highlights weaknesses or potential high-risk alternatives, functioning as an automated ‘devil’s advocate’.

The Physician Agent can optionally consult the Critic Agent before submitting a final diagnosis. The Critic Agent ablation (Extended Data Fig. 2) used the same baseline and differed only by addition of the DDx Critic Agent. The specific instruction governing the Critic Agent is detailed in Supplementary Table 29.

#### Adversarial stress testing

To assess robustness under informational scarcity, we performed an adversarial stress test comparing the Physician Agent’s performance in the standard, factually grounded simulation (baseline) with a perturbed condition designed to reduce evidentiary grounding in the patient narrative.

In the perturbed condition, we removed the chief complaint and the full history summary (including the appended admission medication section when present) from the Patient Agent’s system prompt, leaving the system prompt with no information about the patient (Supplementary Table 30). Without these structured anchors, the LLM-based Patient Agent was forced to confabulate context, generating a symptom history that was often incomplete, inconsistent or laden with unsupported details relative to the ground truth.

The key manipulation was, therefore, the quality and stability of conversational evidence available to the Physician Agent. This design enabled us to quantify how degraded patient-provided information affects diagnostic accuracy and the agent’s decision-time confidence signals. All other experimental settings were held constant across conditions, including model versions, decoding parameters (for example, temperature), tool availability, Physician Agent prompts and the number of repeated runs per case.

#### External benchmark configuration

For VivaBench, benchmark-level comparisons were performed with GPT-OSS and Qwen-3.5, both at *T* = 0.6. The main confidence and triage analyses on VivaBench (Extended Data Fig. 4b–i) used Qwen-3.5 with model-default Hugging Face sampling settings (top_*k* = 20, top_*p* = 0.95). Because VivaBench represents viva-style diagnostic encounters rather than admission-specific emergency workflows, benchmark-specific Patient Agent prompts and Physician Agent tool interfaces were used. The core diagnostic reasoning logic, submission format and confidence-estimation procedures remained unchanged.

#### Sensitivity analyses

Implementation-sensitivity analyses (Extended Data Fig. 5) varied repeated-run count (*N* = 3, 5, 10), decoding temperature (*T* = 0.01, 0.3, 0.6, 0.9) and semantic encoder (MiniLM, MPNet and BGE). Except for the repeated-run sensitivity analysis, all confidence, triage and implementation-sensitivity analyses used *N* = 5 stochastic runs and MiniLM for consistency estimation. In temperature-sensitivity analyses, top_*p* and top_*k* were held at the model-specific default settings listed in Extended Data Table 2.

### Performance evaluation

#### Automated benchmark scoring

We evaluated diagnostic performance using benchmark-specific scoring protocols. For the seven-disease MIRA-v2 benchmark, we used an LLM-based adjudication protocol^29^ (LLM-as-a-judge). The evaluator model was run independently from the clinical agent and received the agent’s predicted diagnosis together with the reference diagnosis label for the case, returning a structured decision on clinical equivalence. All LLM-based adjudication analyses reported here used Gemini 3.1 Flash-Lite^41^ as the evaluator model. The evaluator prompt used for the MIMIC-IV-derived benchmarks is provided in Supplementary Table 31.

For the four-disease CDM benchmark, we used the published fuzzy-matching evaluation procedure^30^, which compares the agent’s final diagnosis with a predefined set of permissible terms for each disease class. This protocol was retained to preserve direct comparability with the original CDM leaderboard and previously reported results.

For VivaBench, we also used an LLM-based adjudication procedure but with a benchmark-specific evaluation prompt adapted to the task format (Supplementary Table 32). VivaBench provides both a diagnosis list and an accepted differentials list, whereas the clinical agent outputs a single final diagnosis. We, therefore, aligned the evaluation to the benchmark’s top-1 approximate accuracy principle^32^: the evaluator assessed the prediction against both the diagnosis list (decision) and the accepted differential list (partial_credit). A case was counted as incorrect only when both decision and partial_credit were false.

**Extended Data Fig. 6:**
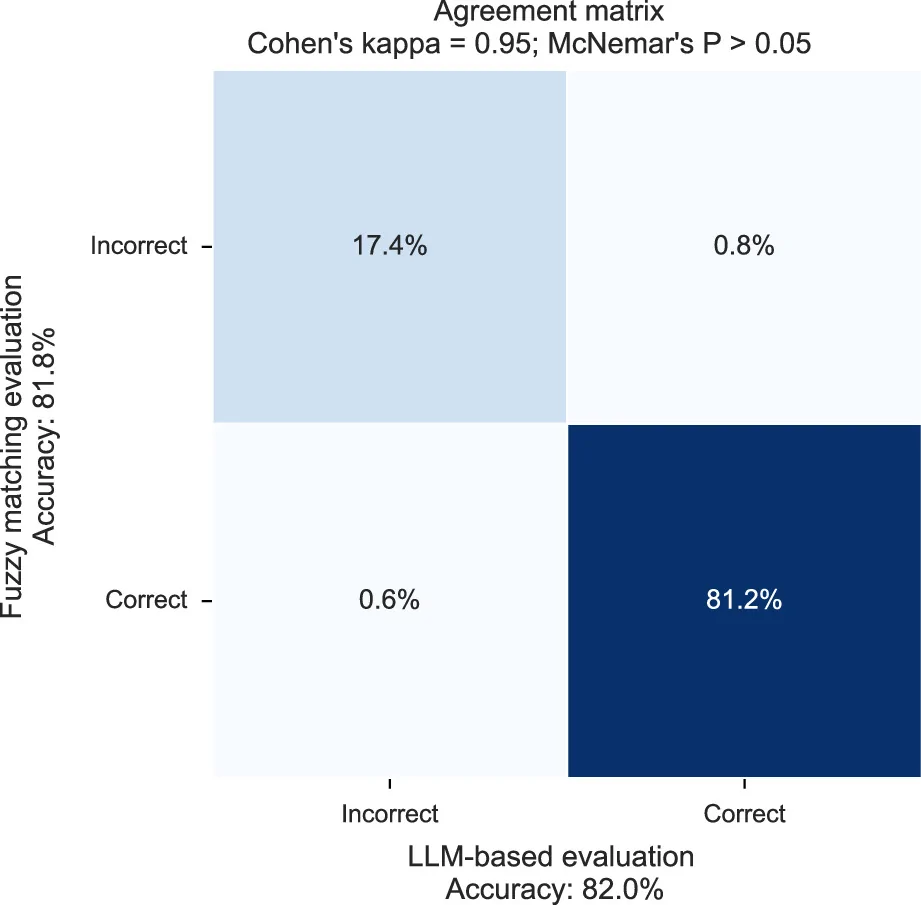
Validation of LLM-based evaluation against standard fuzzy matching. Concordance matrix comparing paired classifications from the LLM-based evaluator and the established fuzzy-matching protocol for run 1 of the CDM benchmark (pooled analysis, n = 2,400 unique benchmark cases; one paired classification per case). Total agreement was 98.6%, with Cohen’s κ = 0.952 (paired-bootstrap 95% CI, 0.936-0.968; 10,000 resamples). The LLM-based evaluator and fuzzy matching yielded accuracies of 82.04% and 81.79%, respectively; the LLM-minus-fuzzy difference was 0.25 percentage points (paired-bootstrap 95% CI, -0.21 to 0.71 percentage points). A two-sided exact McNemar test of the paired classifications identified 20 fuzzy-incorrect/LLM-correct and 14 fuzzy-correct/LLM-incorrect discordant pairs (P = 0.3915). No multiple-comparison adjustment was applied because this was a single prespecified comparison. These results support the use of LLM-based adjudication in settings where rigid term-list matching is impractical. Source data

To assess agreement between LLM-based adjudication and the published fuzzy-matching protocol in a setting where both were applicable, we performed a head-to-head concordance analysis on the CDM dataset. We evaluated a single prespecified run (GLM-4.5-Air; run 1; pooled *n* = 2,400). The two methods showed high agreement (Cohen’s *κ* = 0.95) and did not differ significantly in aggregate accuracy (McNemarʼs test, *P* > 0.05) (Extended Data Fig. 6), supporting the use of LLM-based adjudication when rigid term list matching is impractical.

#### Physician evaluation

To validate automated scoring and assess clinical validity beyond administrative labels, we performed blinded physician review in two rounds.

In round 1, we sampled a stratified random subset of 111 MIRA-v2 cases (approximately 20%), broadly reflecting disease prevalence. Two board-certified physicians independently reviewed each case using the same structured clinical information provided to the agent (summarized history, medications, physical examination, laboratory tests, urinalysis, microbiology and radiology). In phase 1, physicians generated an independent diagnosis without access to the dataset label or the agent output. In phase 2, they assessed the clinical validity of both the dataset reference label and the agent diagnosis (each marked clinically valid or not clinically valid). Disagreements were adjudicated by a third board-certified physician, and the final round 1 outcome was determined by majority vote.

Because some low-prevalence conditions remained underrepresented in round 1, particularly pneumonia (*n* = 6), we conducted a second physician evaluation round to enrich small disease strata. Specifically, we added diverticulitis (+16), pancreatitis (+16) and urinary tract infection (+15) cases to reach approximately 50% physician coverage in those categories and added all remaining pneumonia cases (+23), yielding full physician review for pneumonia (29/29 cases). This produced an additional 70 cases in round 2.

In round 2, six board-certified physicians participated: the three physicians from round 1 and three additional physicians. The 70 cases were randomly partitioned into three fixed subsets: X (*n* = 20), Y (*n* = 25) and Z (*n* = 25). For inter-rater agreement analysis, all six physicians reviewed subset X. The physicians were then split into two groups of three, with one group reviewing X + Y (*n* = 45) and the other reviewing X + Z (*n* = 45). Each case was, therefore, independently reviewed by at least three physicians, and round 2 case-level outcomes were determined by majority vote within the corresponding physician set (details in Supplementary Table 3).

#### Qualitative case review

To characterize failure modes, confidence-state discordance and benchmark-label ambiguity, physicians conducted targeted review of representative cases from both the MIRA-v2 and VivaBench analyses. In MIRA-v2, four prespecified baseline cases were reviewed to represent contrasting confidence profiles: two high-confidence incorrect cases by automated scoring and two low-confidence correct cases. Review focused on missing or misleading evidence, reasoning trajectory and safety-relevant implications for escalation. In VivaBench, we additionally reviewed 10 baseline cases under the Qwen-3.5 configuration that had high ConsistencyDx (≥0.85) but were scored as false by the LLM-based evaluator and, thus, represented cases incorrectly retained in the autonomous stream (‘Autonomous Error’) in the selective-autonomy simulations. Two physicians reviewed these cases to determine whether the evaluator-marked discordances were clinically meaningful errors, whether partial credit was warranted, why the model appeared confident despite disagreement with the benchmark endpoint and what missing or later-available evidence would have changed the answer.

### Multi-perspective confidence framework

We defined decisional trust using reference-independent, inference-time reliability measures computed from the agent’s outputs without access to ground truth labels. The framework captures three complementary dimensions: internal likelihood, expressed uncertainty in language and behavioral stability across repeated stochastic runs. These dimensions were selected to capture distinct aspects of model behavior: token-level preference under a given context, linguistic expression of certainty and semantic stability across repeated outputs. The framework was informed by previous work on self-consistency reasoning, semantic uncertainty estimation and uncertainty-aware diagnostic systems^26,27,42^ but evaluates these signals jointly within an autonomous clinical agent workflow and separately for both the final diagnosis (Dx) and reasoning trace (R). Unless stated otherwise, scores were computed per case. For ProbScore, LingCert and ConceptDensity, run-level values were averaged across runs; Consistency was computed once per case using the full set of runs.

#### Internal Confidence: probabilistic score (ProbScore)

We used token-level likelihood as a lightweight internal likelihood proxy. For each run, let the output text (Dx or R) contain k tokens $\left({t}_{1},\ldots ,{t}_{k}\right)$ generated given the context C. We compute the geometric mean token probability in log space:

$${S}_{\mathrm{prob}}=\exp \left(\frac{1}{k}\sum _{i=1}^{k}\mathrm{log}P({t}_{i}|{t}_{ < i},C)\right)$$

We report this score separately for diagnosis and reasoning as ProbScoreDx and ProbScoreR. Because this metric is derived from token-level generation likelihood within a single run, it may reflect local fluency or model preference without guaranteeing factual correctness or calibration. We, therefore, treated ProbScore as one component of a broader reliability framework rather than as a sufficient confidence measure on its own.

#### Expressed Confidence: linguistic and content cues (LingCert and ConceptDensity)

##### Hedging-based linguistic certainty (LingCert)

We treated linguistic hedging as a lightweight proxy for expressed uncertainty in the generated text. This choice was informed by previous work on hedge/speculation cues and uncertainty expression in biomedical and clinical language. Because our aim was a transparent, audit-friendly metric applicable directly to generated outputs, we implemented LingCert as a rule-based cue density score rather than a learned uncertainty detector. Given an output text with k words and h matched hedging cues, we compute:

$${S}_{\mathrm{ling}}=1-\frac{h}{k+\epsilon }$$

with ϵ=1 for numerical stability. Higher values indicate fewer hedging cues and, thus, higher expressed certainty. We compute LingCertDx and LingCertR. The hedging lexicon comprises 35 terms curated from established biomedical uncertainty language resources^43,44^, covering modal verbs (may, might, could), hedging verbs (suggest, indicate, appear, seem) and probabilistic modifiers (possible, probable, unlikely, uncertain); the full list is provided in Extended Data Table 3.

##### Clinical concept density (ConceptDensity)

To capture whether the output is dominated by clinically meaningful content, we compute the fraction of valid tokens attributable to core clinical concepts. We process text using a biomedical natural language processing (NLP) pipeline (spaCy + scispaCy) with Unified Medical Language System (UMLS) entity linking^45–47^ and retain only entities whose linked concept semantic types (type unique identifiers (TUIs)) fall within a predefined whitelist of core clinical categories (for example, diseases/syndromes, signs/symptoms, findings, anatomical structures and diagnostic/therapeutic procedures; full list in Extended Data Table 4). Let T be the set of valid tokens (excluding punctuation and whitespace), and let ${T}_{\mathrm{core}}\subseteq T$ be tokens covered by whitelisted entity spans. We define:

$${S}_{\mathrm{cd}}=\frac{|{T}_{\mathrm{core}}|}{{|T|}}$$

We compute ConceptDensityDx and ConceptDensityR. ConceptDensity was included as an exploratory content structure measure to test whether concept-rich outputs aligned with diagnostic reliability. As reported in the Results, ConceptDensityR did not behave as a positive confidence signal and was, therefore, not interpreted as a standalone reliability metric.

#### Behavioral Confidence: cross-run stability (Consistency)

We defined Behavioral Confidence as the semantic stability of the model’s output across repeated stochastic runs under identical prompts. This choice was motivated by previous work on self-consistency reasoning and related semantic uncertainty methods^26,42^, but here the resulting cross-run agreement score was used as an inference-time reliability signal rather than as a decoding or answer-selection rule. For each case, we generated *N* independent runs and embedded each output (Dx or R) using a sentence encoder (all-MiniLM). We then computed the mean pairwise cosine similarity across runs:

$$\bar{s}=\frac{2}{N(N-1)}\sum _{1\le i < j\le N}\cos ({e}_{i},{e}_{j})$$

To obtain a score bounded in [0,1], we clipped the mean pairwise cosine similarity to the unit interval:

$${S}_{\mathrm{con}}=\min (1,\max (0,\overline{s}))$$

We report ConsistencyDx and ConsistencyR. This approach is related to self-consistency methods and semantic entropy but differs in that it operates on full-output embeddings rather than discrete answer clusters and is applied separately to diagnostic conclusions and reasoning traces within a multi-step agentic workflow. Sensitivity of Consistency to the number of runs (*N*), embedding model choice and decoding temperature is assessed in the ‘Implementation sensitivity analyses of behavioral consistency’ section.

Unless otherwise stated, confidence analyses used five stochastic runs per case and MiniLM as the default sentence encoder for consistency estimation.

### Statistical analysis

Discriminative performance of reliability-related metrics was quantified using ROC analysis and summarized by the AUC. Bootstrapped 95% confidence intervals were computed for all AUC estimates. For the primary comparison between ConsistencyDx and ProbScoreDx, differences between correlated ROC curves were assessed using DeLong’s test; FDR-adjusted *P* values are reported where applicable. Detailed AUC summaries and confidence intervals are provided in the corresponding supplementary tables. Correlations among reliability-related metrics were assessed using Pearsonʼs correlation coefficients. Differences in score distributions between correct and incorrect cases were assessed using two-sided Mann−Whitney *U*-tests, with Holm correction applied across the family of metrics compared in the same analysis.

For threshold-based selective-autonomy analyses, retained-set accuracy and coverage were summarized with 95% Wilson confidence intervals. Threshold analyses were treated as descriptive operating characteristic analyses over predefined threshold grids rather than as formal threshold optimization procedures.

For the stress test analyses, baseline and perturbed conditions were paired at the case level (*n* = 551). Paired differences in diagnostic correctness (binary per case) were assessed using the exact McNemarʼs test, whereas paired differences in continuous confidence metrics were assessed using two-sided Wilcoxon signed-rank tests. Where multiple paired comparisons were performed within the same figure or analysis family, *P* values were adjusted using the Holm method.

For implementation-sensitivity analyses, paired differences in per-case ConsistencyDx values across repeated-run counts, decoding temperatures or embedding models were assessed using the Friedman test. Threshold-specific differences in binary coverage status across conditions were assessed using Cochran’s *Q* test. When correctness was compared across conditions within the subset of jointly retained cases, Cochran’s *Q* test was again used, restricted to the complete paired intersection.

Agreement between evaluation methods on paired case-level outcomes was assessed using Cohen’s *κ* and McNemarʼs test. Agreement between automated judging and physician adjudication was summarized using Gwet’s AC1, together with overall agreement and discordance counts. All statistical tests were two-sided unless otherwise stated.

### Use of AI tools

In accordance with the Committee on Publication Ethics (COPE) position statement of 13 February 2023 (https://publicationethics.org/cope-position-statements/ai-author), the authors hereby disclose the use of the following AI model during the writing of this article: GPT-5.2 (OpenAI) for checking spelling and grammar.

### Reporting summary

Further information on research design is available in the Nature Portfolio Reporting Summary linked to this article.

## Online content

Any methods, additional references, Nature Portfolio reporting summaries, source data, extended data, supplementary information, acknowledgements, peer review information; details of author contributions and competing interests; and statements of data and code availability are available at https://doi.org/10.1038/s41591-026-04609-x.

## Supplementary information

Supplementary Information Supplementary Fig. 1 and Tables 1−38.

Reporting Summary

Peer Review File

## Source data

Source Data Fig. 2 Diagnostic performance of a fully on-premise clinical agent across standardized benchmarks.

Source Data Figs. 3, 4 and 6 and Extended Data Fig. 3 Table with final diagnoses and all the confidence features.

Source Data Fig. 5 Stress testing decisional trust under induced information scarcity.

Source Data Extended Data Fig. 1 Benchmark demographic composition and subgroup diagnostic accuracy. The sheets with names starting from ‘cohort’ are related to panel a; the others are related to panels b and c.

Source Data Extended Data Fig. 2 Effect of the DDx Critic Agent on diagnostic accuracy.

Source Data Extended Data Fig. 4 External benchmark evaluation and triage simulation on VivaBench.

Source Data Extended Data Fig. 5 Implementation sensitivity analyses of behavioral consistency.

Source Data Extended Data Fig. 6 Validation of LLM-based evaluation against standard fuzzy matching.

## Data Availability

MIRA-v2 and CDM are derived from MIMIC-IV version 2.2 and are subject to PhysioNet credentialed access requirements, including completion of the required training and signing of the PhysioNet data use agreement for credentialed health data. MIRA-v2 can be reconstructed from MIMIC-IV version 2.2 using the code released by the benchmark developers at https://github.com/Dyke-F/MIRA. CDM can be reconstructed using the code released by the benchmark authors at https://github.com/paulhager/MIMIC-Clinical-Decision-Making-Dataset and is also available to credentialed users through its PhysioNet release at https://www.physionet.org/content/mimic-iv-ext-cdm/1.0/. VivaBench is publicly available under a CC BY 4.0 license on Hugging Face at https://huggingface.co/datasets/chychiu/VivaBench. Source data are provided with this paper.

## Extended data

**Extended Data Table 1:** Diagnostic Toolset for the Physician Agent

Tool Name	Parameters	Description	Example Output
Physical Exam	None	Retrieves comprehensive physical examination findings.	“Vitals: T: 100.0F HR: 125…”
Laboratory Test	Test Names	Orders specific quantitative laboratory tests from the available panel.	“White Blood Cells: 14.5 K/uL (Ref: 4-10).”
Urine Test	Test Names	Orders specific quantitative urinalysis markers.	“RBC: 14.0 #/hpf (Reference: 0.0 - 2.0)”
Radiology	Modality, Body Region	Requests diagnostic imaging studies. Returns the radiologist’s interpretation report.	“Radiology Report (Radiograph, Chest): …atelectasis versus pneumonia.”
Microbiology	Culture Type	Orders cultures to identify infectious pathogens in blood, urine, or other fluids.	“Blood Culture: No Growth.”
Bedside Test (VivaBench Only)	Test Names	Order bedside investigations such as ECG or other point-of-care tests.	â€œ- Electrocardiogram: evidence consistent with left ventricular hypertrophyâ€
Other Investigation (VivaBench Only)	Test Names	For non-blood, non-urine, non-microbiology, non-bedside investigations from remaining sections	â€œ- Peripheral Blood Film: fragmented red blood cells, increased polychromasia, thrombocytopeniaâ€
Admission	Final Diagnosis, Clinical Reasoning	Concludes the encounter. Submits the definitive diagnosis and a summarized reasoning trace, which serves as the basis for confidence assessment.	“Diagnosis: Pneumonia in patient with stage IV NSCLC” “Reasoning: Patient presented with symptoms of coughâ€¦â€

This table lists the diagnostic tools available to the Physician Agent for acquiring objective clinical evidence during multi-turn reasoning. Each episode concludes with a final submission action (“Admission”), which returns a diagnostic output and a structured reasoning trace used in subsequent reliability analyses. The full MIRA-v2 framework also includes therapeutic tools, but the present study focuses on the diagnostic toolset. For VivaBench, two additional diagnostic tools were added to cover investigation categories not present in the MIMIC-IV–derived benchmarks.

**Extended Data Table 2:** Model sources, inference frameworks, and deployment settings

Model Name	Identifier	Source / access	Inference framework	Default T, top-p, top-k	GPU
GLM-4.5-Air	zai-org/GLM-4.5-Air-FP8	Hugging Face weights; on-premise deployment	vLLM	1.0; 1.0; 0	2x H200
GLM-5	zai-org/GLM-5-FP8	Hugging Face weights; on-premise deployment	vLLM	1.0; 0.95; 0	8x H200
GPT-OSS	openai/gpt-oss-120b	Hugging Face weights; on-premise deployment	vLLM	1.0; 1.0; 0	1x H200
Qwen-3.5	Qwen/Qwen3.5-397B-A17B-FP8	Hugging Face weights; on-premise deployment	vLLM	0.6; 0.95; 20	4x H200
MedGemma	google/medgemma-27b-it	Hugging Face weights; on-premise deployment	vLLM	1.0; 1.0; 0	1x RTX PRO 6000 Blackwell
Gemini 3.1 Flash-Lite	google/gemini-3.1-flash-lite-preview	OpenRouter	OpenRouter API	1.0; 0.95; 64	
GPT-5.2	gpt-5.2-2025-12-11	OpenAI	OpenAI API		
all-MiniLM	sentence-transformers/all-MiniLM-L6-v2	Hugging Face	Sentence-Transformers		
MPNet	sentence-transformers/all-mpnet-base-v2	Hugging Face	Sentence-Transformers		
BGE	BAAI/bge-base-en-v1.5	Hugging Face	Sentence-Transformers		
(leaderboard) Gemma-3	google/gemma-3-27b-it	Not reported (leaderboard)	Not reported		

Note: H200 and RTX PRO 6000 Blackwell refer to NVIDIA GPUs. Hardware was reported only for locally hosted models; provider-hosted API models were listed as not reported.

**Extended Data Table 3:** Hedging Lexicon for the Linguistic Certainty Score (LingCert)

Lexicon version	Count	Phrase / Word
Original (compact)	15	possible, likely, suspected, cannot rule out, vs, might, probable, concern for, suggests, worrisome for, appears, indeterminate, question of, consider, suspicious for
Broad (inclusive, unfiltered)	48	Original (compact) + unlikely, certain, never, sometimes, rarely, often, almost, perhaps, occasionally, frequent, compatible with, improbable, always, probably, consistent with, possibly, not unreasonable, doubtful, not certain, low probability, usually, suggestive, not likely, low chance, no evidence of, possibility of, versus, uncertain, unclear, maybe, suspected, equivocal, not excluded
Refined (curated)	35	may, might, could, suggest, suggests, suggesting, indicate, indicates, appear, appears, seem, seems, suspect, suspected, possible, possibly, probable, probably, likely, unlikely, potential, potentially, presumed, uncertain, unclear, questionable, equivocal, improbable, doubtful, suggestive, approximate, perhaps, maybe, presumably, apparently

Three lexicon versions were evaluated: an original compact lexicon of 15 terms, a broad lexicon of 48 terms informed by BioScope and clinical EHR terminology, and a refined lexicon of 35 unambiguous single-word epistemic uncertainty terms. The refined lexicon was used for the analyses reported in the main text. Full AUC comparison across lexicon versions is provided in Supplementary Table S33.

**Extended Data Table 4:** Whitelist of core clinical concept categories for ConceptDensity

UMLS TUI	Semantic type (UMLS)
T047	Disease or Syndrome
T048	Mental or Behavioral Dysfunction
T184	Sign or Symptom
T033	Finding
T023	Body Part, Organ, or Organ Component
T060	Diagnostic Procedure
T059	Laboratory Procedure
T061	Therapeutic or Preventive Procedure
T121	Pharmacologic Substance
T195	Antibiotic
T109	Organic Chemical

This table lists the UMLS semantic types (TUIs) treated as core clinical concept categories when computing ConceptDensity.

## Code availability

All code in this study is available at https://github.com/KatherLab/onprem-medical-agents and archived on Zenodo at https://doi.org/10.5281/zenodo.20233449 (ref. ^48^) under a CC BY 4.0 license.
