# Supplementary material for: On-premise medical AI agents for reliable clinical decision-making
Source: Nat Med. 2026 Sep 15;32(10):3705–16. doi: 10.1038/s41591-026-04609-x (PMC13668652; doi:10.1038/s41591-026-04609-x)
Supplement: Supplementary file 1 [file 41591_2026_4609_MOESM1_ESM.pdf]

nature medicine

Supplementary information https://doi.org/10.1038/s41591-026-04609-x

On-premise medical AI agents for reliable clinical decision-making

In the format provided by the authors and unedited

Supplementary

Fig. S1 | Post-hoc analysis of reasoning-trace concept density against diagnostic process variables.

(a,b) Scatter plots of case-level ConceptDensityR versus the mean number of diagnostic tool calls (a) and retrieved evidence items (b). Each point represents one unique MIRA-v2 benchmark case (n = 551; correct, n = 499; incorrect, n = 52); blue and red points denote correct and incorrect diagnostic outcomes, respectively. Case-level ConceptDensityR and process variables were computed from five stochastic model inferences per case; ToolCallCount_mean and EvidenceRetrievedCount_mean denote means across the five runs. These computational repeats were not treated as independent observations. Black lines show least-squares linear visual guides. Two-sided Spearman rank correlations, with Holm adjustment across the two post-hoc comparisons, showed weak positive associations of ConceptDensityR with mean diagnostic tool calls (ρ = 0.131, P_Holm = 0.002) and retrieved evidence items (ρ = 0.266, P_Holm = 4.287×10^{-10}).

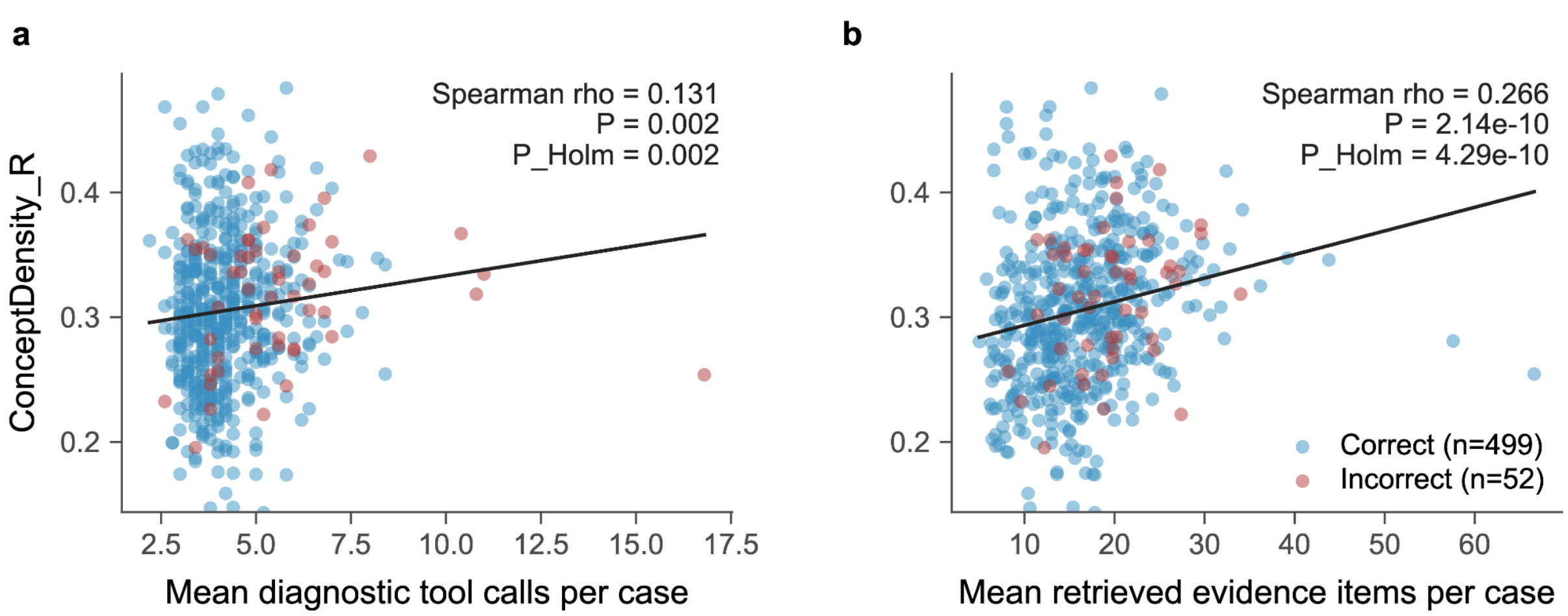

Table S1 | Per-disease accuracy of MIRA-v2 and CDM across models and temperature settings.

GLM-4.5-Air					
MIRA-v2					CDM
	T=0.01	T=0.3	T=0.6	T=0.9	T=0.01

Overall	**88.42 ± 0.87**	87.11 ± 1.14	88.09 ± 0.89	86.68 ± 0.47	81.24 ± 0.55
Appendicitis	97.16 ± 1.00	98.51 ± 0.74	98.51 ± 0.30	97.84 ± 0.74	96.28 ± 0.35
Cholecystitis	87.13 ± 1.70	87.13 ± 2.61	88.37 ± 3.24	86.05 ± 1.10	71.82 ± 0.76
Diverticulitis	92.59 ± 2.27	91.48 ± 1.01	91.11 ± 2.03	92.96 ± 2.41	68.79 ± 1.59
Pancreatitis	88.08 ± 5.16	86.54 ± 1.36	89.62 ± 3.49	87.69 ± 3.49	71.78 ± 2.11
Pneumonia	54.48 ± 6.63	54.48 ± 8.23	57.93 ± 6.63	53.10 ± 5.77	
Pulm. embolism	87.56 ± 0.93	80.89 ± 1.83	83.33 ± 1.76	82.22 ± 2.36	
UTI	82.86 ± 4.47	79.18 ± 3.03	77.55 ± 4.08	74.69 ± 4.70	
**GLM-5**					
MIRA-v2					
	T=0.01	T=0.3	T=0.6	T=0.9	
Overall	89.11 ± 1.13	**89.65 ± 0.78**	88.78 ± 0.66	88.78 ± 0.75	
Appendicitis	98.24 ± 0.37	98.65 ± 0.68	98.24 ± 0.60	98.78 ± 0.30	
Cholecystitis	81.40 ± 2.85	82.30 ± 1.65	81.71 ± 1.78	80.00 ± 2.01	
Diverticulitis	93.70 ± 1.01	94.44 ± 1.31	92.96 ± 0.83	94.44 ± 1.31	
Pancreatitis	97.69 ± 1.61	96.15 ± 1.92	97.69 ± 1.61	96.92 ± 1.72	
Pneumonia	60.69 ± 3.93	62.76 ± 8.59	58.62 ± 8.79	57.93 ± 4.50	
Pulm. embolism	91.78 ± 1.69	90.44 ± 2.79	89.78 ± 2.14	90.67 ± 2.17	
UTI	79.59 ± 4.56	84.08 ± 2.24	80.82 ± 3.10	81.63 ± 3.23	
**Qwen-3.5**					
MIRA-v2					CDM
	T=0.01	T=0.3	T=0.6	T=0.9	T=0.6
Overall	89.73 ± 0.38	89.33 ± 0.87	89.87 ± 0.75	**90.04 ± 1.14**	83.78 ± 0.47
Appendicitis	97.97 ± 0.48	98.38 ± 0.37	98.78 ± 0.57	98.78 ± 0.30	96.59 ± 0.16
Cholecystitis	85.27 ± 2.33	85.89 ± 1.39	86.36 ± 1.78	86.67 ± 2.82	71.17 ± 0.63
Diverticulitis	90.37 ± 3.04	89.63 ± 2.11	90.37 ± 2.41	90.00 ± 1.66	73.23 ± 0.51
Pancreatitis	96.92 ± 2.19	95.00 ± 1.72	96.92 ± 1.05	98.46 ± 0.86	81.23 ± 1.23
Pneumonia	66.21 ± 3.78	62.07 ± 4.22	62.07 ± 3.45	60.69 ± 5.23	
Pulm. embolism	90.22 ± 1.45	91.11 ± 2.22	90.44 ± 1.27	91.76 ± 2.56	
UTI	81.22 ± 2.66	77.55 ± 3.82	79.59 ± 6.29	77.87 ± 1.61	

GPT-OSS					
MIRA-v2					
	T=0.01	T=0.3	T=0.6	T=0.9	
Overall	**85.30 ± 0.94**	85.05 ± 1.27	84.75 ± 0.43	84.25 ± 0.75	
Appendicitis	97.97 ± 1.07	97.97 ± 0.83	97.16 ± 0.57	97.70 ± 1.40	
Cholecystitis	81.40 ± 1.90	81.24 ± 2.71	78.91 ± 3.21	78.91 ± 1.01	
Diverticulitis	90.37 ± 3.04	89.26 ± 1.55	92.22 ± 3.31	90.74 ± 2.27	
Pancreatitis	93.85 ± 2.51	93.46 ± 3.49	94.62 ± 0.86	91.92 ± 2.51	
Pneumonia	60.69 ± 3.08	55.86 ± 5.11	53.79 ± 4.63	57.93 ± 2.89	
Pulm. embolism	81.33 ± 3.96	83.11 ± 2.41	83.33 ± 3.24	80.89 ± 5.29	
UTI	64.49 ± 4.23	63.27 ± 5.00	64.90 ± 4.87	64.08 ± 3.41	
**GPT-5.2**					
MIRA-v2					
	T=0.01				
Overall	90.71 ± 0.90				
Appendicitis	97.70 ± 0.60				
Cholecystitis	85.58 ± 2.36				
Diverticulitis	92.59 ± 2.27				
Pancreatitis	99.23 ± 1.05				
Pneumonia	65.52 ± 7.31				
Pulm. embolism	94.22 ± 1.22				
UTI	80.41 ± 3.71				

Table S2 | Distribution of physician-reviewed cases across diagnostic categories.

Round 1 used stratified random sampling at approximately 20% coverage. Round 2 expanded coverage in low-prevalence categories: diverticulitis, pancreatitis, and UTI to ~50%, and pneumonia to 100% (full census).

Disease	Total N	Round 1 (n)	Round 2 (n)	Total reviewed (n)	% of total
Appendicitis	148	30	0	30	20
Cholecystitis	129	26	0	26	20

Pulmonary embolism	90	18	0	18	20
Diverticulitis	54	11	16	27	50
Pancreatitis	52	10	16	26	50
UTI	49	10	15	25	51
Pneumonia	29	6	23	29	100
Total	551	111	70	181	33

Table S3 | Per-disease physician consensus on diagnostic validity.

Disease	Both Valid (%)	GT Valid Only (%)	Agent Valid Only (%)	Neither Valid (%)
Appendicitis	96.7	3.3	0	0
Cholecystitis	92.3	7.7	0	0
Diverticulitis	88.9	0	11.1	0
Pancreatitis	92.3	7.7	0	0
Pneumonia	44.8	41.4	10.3	3.5
Pulmonary Embolism	94.4	5.6	0	0
UTI	68.0	20.0	8.0	4.0
Overall	81.8	12.7	4.4	1.1

Table S4 | Inter-rater agreement for physician adjudication across review rounds.

Two rounds of blinded physician review were conducted. Round 1 (n = 111) used two primary reviewers with a third adjudicator for disagreements. Round 2 (n = 70; with 20 cases reviewed by six physicians for inter-rater agreement analysis) expanded coverage for low-prevalence conditions using six reviewers.

Round	Physicians	Cases	Task	Raw Agreement	Gwet's AC1	PABAK
1	2	111	gt validity	0.964	0.963	0.928
1	2	111	agent validity	0.937	0.929	0.874
2	6	20	gt validity	0.867	0.837	0.733
2	6	20	agent validity	0.837	0.700	0.673

Table S5 | Per-disease agreement between LLM-based evaluator and physician consensus on agent-diagnosis validity.

Disease	Cases	Raw Agreement	Gwet's AC1	PABAK
Appendicitis	30	1.000	1.000	1.000
Cholecystitis	26	0.962	0.953	0.923
Diverticulitis	27	0.963	0.962	0.926
Pancreatitis	26	1.000	1.000	1.000
Pneumonia	29	0.724	0.454	0.448
Pulmonary Embolism	18	0.944	0.934	0.889
UTI	25	0.880	0.830	0.760

Table S6 | ROC AUC summary for MIRAv2 baseline analyses corresponding to Fig. 3 and Extended Data Fig. E3.

Area under the receiver operating characteristic curve (AUC) for all evaluated confidence and reliability-related metrics under baseline conditions, reported for the overall cohort and for each disease category, separately for the diagnostic output and reasoning trace. Lower and upper bounds indicate bootstrapped 95% confidence intervals based on 2,000 bootstrap iterations.

Dataset	Stream	Metric	AUC	95%CI
Overall	Diagnosis	ConceptDensityDx	0.674	[0.616,0.729]
Overall	Diagnosis	ConsistencyDx	0.860	[0.804,0.908]
Overall	Diagnosis	LingCertDx	0.629	[0.558,0.698]
Overall	Diagnosis	ProbScoreDx	0.747	[0.665,0.818]
Overall	Reasoning	ConceptDensityR	0.426	[0.350,0.505]
Overall	Reasoning	ConsistencyR	0.785	[0.703,0.859]
Overall	Reasoning	LingCertR	0.792	[0.733,0.848]
Overall	Reasoning	ProbScoreR	0.721	[0.649,0.784]
appendicitis	Diagnosis	ConceptDensityDx	0.901	[0.828,0.966]
appendicitis	Diagnosis	ConsistencyDx	0.991	[0.968,1.000]
appendicitis	Diagnosis	LingCertDx	0.469	[0.448,0.486]
appendicitis	Diagnosis	ProbScoreDx	0.913	[0.821,0.984]
appendicitis	Reasoning	ConceptDensityR	0.379	[0.209,0.566]
appendicitis	Reasoning	ConsistencyR	0.956	[0.894,1.000]
appendicitis	Reasoning	LingCertR	0.926	[0.883,0.966]
appendicitis	Reasoning	ProbScoreR	0.738	[0.506,0.972]
cholecystitis	Diagnosis	ConceptDensityDx	0.831	[0.700,0.936]
cholecystitis	Diagnosis	ConsistencyDx	0.711	[0.568,0.838]
cholecystitis	Diagnosis	LingCertDx	0.544	[0.449,0.667]

cholecystitis	Diagnosis	ProbScoreDx	0.595	[0.414,0.752]
cholecystitis	Reasoning	ConceptDensityR	0.373	[0.204,0.555]
cholecystitis	Reasoning	ConsistencyR	0.661	[0.453,0.847]
cholecystitis	Reasoning	LingCertR	0.806	[0.679,0.915]
cholecystitis	Reasoning	ProbScoreR	0.772	[0.635,0.887]
diverticulitis	Diagnosis	ConceptDensityDx	0.078	[0.026,0.163]
diverticulitis	Diagnosis	ConsistencyDx	0.595	[0.069,0.935]
diverticulitis	Diagnosis	LingCertDx	0.392	[0.333,0.451]
diverticulitis	Diagnosis	ProbScoreDx	0.484	[0.039,0.817]
diverticulitis	Reasoning	ConceptDensityR	0.712	[0.510,0.902]
diverticulitis	Reasoning	ConsistencyR	0.562	[0.176,0.869]
diverticulitis	Reasoning	LingCertR	0.425	[0.196,0.765]
diverticulitis	Reasoning	ProbScoreR	0.680	[0.255,0.954]
pancreatitis	Diagnosis	ConceptDensityDx	0.635	[0.401,0.839]
pancreatitis	Diagnosis	ConsistencyDx	0.948	[0.859,1.000]
pancreatitis	Diagnosis	LingCertDx	0.448	[0.406,0.490]
pancreatitis	Diagnosis	ProbScoreDx	0.583	[0.213,0.953]
pancreatitis	Reasoning	ConceptDensityR	0.651	[0.474,0.823]
pancreatitis	Reasoning	ConsistencyR	0.625	[0.260,0.953]
pancreatitis	Reasoning	LingCertR	0.422	[0.198,0.693]
pancreatitis	Reasoning	ProbScoreR	0.609	[0.318,0.875]
pneumonia	Diagnosis	ConceptDensityDx	0.693	[0.490,0.879]
pneumonia	Diagnosis	ConsistencyDx	0.781	[0.586,0.929]
pneumonia	Diagnosis	LingCertDx	0.748	[0.562,0.914]
pneumonia	Diagnosis	ProbScoreDx	0.781	[0.586,0.924]
pneumonia	Reasoning	ConceptDensityR	0.467	[0.243,0.695]
pneumonia	Reasoning	ConsistencyR	0.757	[0.562,0.924]
pneumonia	Reasoning	LingCertR	0.676	[0.464,0.871]
pneumonia	Reasoning	ProbScoreR	0.529	[0.319,0.752]
pulmonary_embolism	Diagnosis	ConceptDensityDx	0.068	[0.017,0.133]
pulmonary_embolism	Diagnosis	ConsistencyDx	0.917	[0.817,0.986]
pulmonary_embolism	Diagnosis	LingCertDx	0.578	[0.444,0.725]
pulmonary_embolism	Diagnosis	ProbScoreDx	0.779	[0.594,0.934]
pulmonary_embolism	Reasoning	ConceptDensityR	0.516	[0.356,0.681]
pulmonary_embolism	Reasoning	ConsistencyR	0.864	[0.709,0.973]
pulmonary_embolism	Reasoning	LingCertR	0.891	[0.805,0.959]
pulmonary_embolism	Reasoning	ProbScoreR	0.649	[0.450,0.826]
uti	Diagnosis	ConceptDensityDx	0.705	[0.446,0.926]
uti	Diagnosis	ConsistencyDx	0.826	[0.670,0.953]
uti	Diagnosis	LingCertDx	0.705	[0.465,0.930]
uti	Diagnosis	ProbScoreDx	0.702	[0.477,0.911]

uti	Reasoning	ConceptDensityR	0.705	[0.500,0.876]
uti	Reasoning	ConsistencyR	0.860	[0.733,0.957]
uti	Reasoning	LingCertR	0.616	[0.357,0.868]
uti	Reasoning	ProbScoreR	0.651	[0.403,0.872]

Table S7 | Variance inflation factors for the top-performing reliability-related metrics.

All variance inflation factors were low (all < 5), indicating that the retained metrics were not strongly collinear and could be jointly interpreted in multivariable analyses.

Metric	VIF Value
ConsistencyDx	2.26
ConsistencyR	1.97
LingcertR	1.50
ProbscoreDx	1.47
ProbscoreR	1.35

Table S8 | ROC AUC summary for the perturbed stress-test condition corresponding to Fig. 5c.

Area under the receiver operating characteristic curve (AUC) for all evaluated metrics in the perturbed condition, reported separately for the diagnostic output and reasoning trace. Lower and upper bounds indicate bootstrapped 95% confidence intervals.

Dataset	Stream	Metric	AUC	95% CI
Overall	Diagnosis	ConceptDensityDx	0.686	[0.644,0.731]
Overall	Diagnosis	ConsistencyDx	0.875	[0.845,0.905]
Overall	Diagnosis	LingCertDx	0.636	[0.594,0.678]
Overall	Diagnosis	ProbScoreDx	0.723	[0.678,0.767]
Overall	Reasoning	ConceptDensityR	0.457	[0.404,0.506]
Overall	Reasoning	ConsistencyR	0.793	[0.751,0.836]
Overall	Reasoning	LingCertR	0.785	[0.743,0.824]
Overall	Reasoning	ProbScoreR	0.765	[0.720,0.806]

Table S9 | Accuracy–coverage operating points for ConsistencyDx thresholds on MIRA-v2.

Coverage was defined as the proportion of cases retained above the specified ConsistencyDx threshold. Coverage and retained accuracy are reported with case-level 95% confidence

intervals computed using the Wilson score interval. Threshold analyses are descriptive and were not used to optimize a deployment threshold on an independent validation set.

dataset	threshold	accuracy	accuracy_ci	coverage	coverage_95CI
Overall	0.5	0.927	[0.901,0.946]	0.964	[0.945,0.976]
Overall	0.55	0.938	[0.914,0.956]	0.944	[0.921,0.960]
Overall	0.6	0.943	[0.919,0.960]	0.918	[0.892,0.938]
Overall	0.65	0.948	[0.925,0.965]	0.875	[0.845,0.900]
Overall	0.7	0.963	[0.941,0.977]	0.828	[0.794,0.857]
Overall	0.75	0.969	[0.948,0.982]	0.766	[0.729,0.799]
Overall	0.8	0.974	[0.953,0.986]	0.708	[0.668,0.744]
Overall	0.85	0.974	[0.951,0.986]	0.626	[0.585,0.666]
Overall	0.9	0.989	[0.968,0.996]	0.494	[0.452,0.535]
Overall	0.95	0.994	[0.968,0.999]	0.316	[0.278,0.356]

Table S10 | Per-specialty group accuracy of VivaBench.

Specialty Group	Model	Top-1 approximate accuracy (% ± SD)
Overall	GPT-OSS	67.96 ± 1.09
Overall	Qwen-3.5	72.22 ± 0.92
Endocrine & Reproductive	GPT-OSS	72.80 ± 2.23
Endocrine & Reproductive	Qwen-3.5	82.13 ± 1.85
Infectious Disease & Immunology	GPT-OSS	65.07 ± 3.22
Infectious Disease & Immunology	Qwen-3.5	70.27 ± 1.74
Cardiovascular & Metabolic	GPT-OSS	69.46 ± 1.93
Cardiovascular & Metabolic	Qwen-3.5	72.16 ± 1.75
Gastrointestinal	GPT-OSS	66.80 ± 2.28
Gastrointestinal	Qwen-3.5	68.30 ± 2.66
Neurological / Psychiatric	GPT-OSS	75.15 ± 2.23
Neurological / Psychiatric	Qwen-3.5	78.68 ± 1.56
Hematology / Oncology / Other	GPT-OSS	61.43 ± 3.05
Hematology / Oncology / Other	Qwen-3.5	66.96 ± 2.19
Pediatric	GPT-OSS	63.19 ± 3.01
Pediatric	Qwen-3.5	61.16 ± 3.46
Respiratory	GPT-OSS	58.04 ± 2.24
Respiratory	Qwen-3.5	67.06 ± 6.99
Musculoskeletal & Pain	GPT-OSS	76.15 ± 4.21
Musculoskeletal & Pain	Qwen-3.5	76.15 ± 3.22

Table S11 | ROC AUC summary for VivaBench baseline analyses

The table reports the AUC of each confidence metric with its corresponding 95% confidence interval in brackets.

Dataset	Stream	Metric	AUC	95% CI
vivabench_pubmed	Diagnosis	ConceptDensityDx	0.557	[0.517,0.595]
vivabench_pubmed	Diagnosis	ConsistencyDx	0.719	[0.684,0.756]
vivabench_pubmed	Diagnosis	LingCertDx	0.555	[0.523,0.587]
vivabench_pubmed	Diagnosis	ProbScoreDx	0.525	[0.486,0.567]
vivabench_pubmed	Reasoning	ConceptDensityR	0.593	[0.552,0.636]
vivabench_pubmed	Reasoning	ConsistencyR	0.684	[0.645,0.722]
vivabench_pubmed	Reasoning	LingCertR	0.616	[0.576,0.657]
vivabench_pubmed	Reasoning	ProbScoreR	0.547	[0.506,0.586]

Table S12 | Accuracy–coverage operating points for diagnosis confidence thresholds over VivaBench.

dataset	threshold	accuracy	accuracy_95CI	coverage	coverage_95CI
vivabench_pubmed	0.7	0.862	[0.830,0.889]	0.528	[0.497,0.559]
vivabench_pubmed	0.75	0.868	[0.834,0.896]	0.459	[0.428,0.490]
vivabench_pubmed	0.8	0.89	[0.855,0.917]	0.395	[0.365,0.426]
vivabench_pubmed	0.85	0.899	[0.861,0.928]	0.320	[0.292,0.350]
vivabench_pubmed	0.9	0.886	[0.838,0.921]	0.230	[0.205,0.258]
vivabench_pubmed	0.95	0.877	[0.809,0.923]	0.131	[0.112,0.154]

Table S13 | Physician qualitative review of high-consistency missed-error cases on VivaBench

Cases were selected from the Qwen-3.5 baseline configuration with ConsistencyDx ≥ 0.85 and LLM evaluator = false, corresponding to autonomous errors in the selective-autonomy simulations.

Case ID	Model output	Benchmark endpoint	physician	Clinical assessment	Likely error type	Why high confidence?	Missing or later-available evidence	Short note
1	Multiple myeloma	Primary hyperparathyroidism due to parathyroid adenoma	A	Clinically meaningful error; no partial credit	Stable but incorrect reasoning	Anchoring on a familiar high-calcium/CRAB-like syndrome	PTH testing	Model converged on a plausible but incorrect common diagnosis and failed to exclude hyperparathyroidism.
2	Supracondylar fracture of left humerus	Reset osmostat	A	Partially correct but missed main diagnostic problem	Timepoint mismatch / benchmark ambiguity	Locked onto the presenting complaint	Broader work-up for hyponatremia	The fracture was real, but the clinically relevant diagnostic problem was the cause of hyponatremia.

3	POEMS syndrome	Metallosis with systemic cobalt/chromium toxicity	A	Clinically meaningful error; no partial credit	Stable but incorrect reasoning	Syndrome-pattern matching to POEMS features	Metal ion testing	Model overcommitted to a rare but superficially matching syndrome without excluding metallosis.
4	Multiple myeloma	Microscopic polyangiitis	A	Clinically meaningful error; no partial credit	Stable but incorrect reasoning	Anchoring on CRAB-like features suggestive of myeloma	Broader biological testing and ANCA-focused work-up	Model favored a common myeloma pattern instead of identifying ANCA-associated vasculitis.
5	Community-acquired pneumonia	Granulomatosis with polyangiitis	A	Clinically meaningful error; no partial credit	Timepoint mismatch / benchmark ambiguity	Bias toward common first-presentation etiology	ANCA testing and fuller systemic evaluation	Model treated the presentation as CAP and did not escalate the vasculitis differential.
6	Thyrotoxic periodic paralysis	Factitious disorder imposed on self / salbutamol overdose	A	Clinically meaningful error; no partial credit	Stable but incorrect reasoning	Classical syndrome fit despite incomplete confirmation	Thyroid-function testing; recognition of concealed self-induced cause	Model committed to a plausible syndrome, but the true diagnosis required recognizing concealed self-harm behavior.
7	IgA nephropathy	Hemolytic anemia secondary to SARS-CoV-2 infection	A	Clinically meaningful error; no partial credit	Stable but incorrect reasoning	Anchoring on apparent hematuria in a young patient	Recognition that pigment represented hemoglobinuria from hemolysis	Model interpreted the urinary finding as renal bleeding rather than hemolysis-related pigmenturia.
8	Acute pericarditis (model suspicion)	Tuberculosis	B	Reasonable initial diagnosis; partial credit likely warranted	Timepoint mismatch / benchmark ambiguity	Model identified a pathognomonic acute syndrome relevant at presentation	Delayed tuberculosis testing and later etiologic confirmation	In an acute setting, flagging pericarditis was judged more important than inferring tuberculosis before delayed test results returned.
9	STEMI constellation	Takotsubo syndrome	B	Reasonable initial diagnosis; partial credit likely warranted	Timepoint mismatch / benchmark ambiguity	Model captured the actionable presenting syndrome	Coronary angiography and later identification of apical ballooning	STEMI was judged the correct presentation-level diagnosis; Takotsubo became diagnosable only after CAG.
10	Lung abscess / severe pneumonia-type process	Autoimmune disease diagnosed later in admission	B	Reasonable initial diagnosis; partial credit likely warranted	Timepoint mismatch / benchmark ambiguity	Model prioritized the admission-worthy acute process	Later history (epistaxis) and delayed autoimmune synthesis	Model appropriately flagged an admission-level pulmonary process; the autoimmune diagnosis emerged only after later clinical evolution.

Table S14 | Threshold-stratified accuracy and coverage for consistency-based case retention across repeated runs.

For each ConsistencyDx threshold, results are reported separately for N=3, N=5, and N=10 repeated runs. Accuracy denotes the proportion of correct final decisions among retained cases, where retained cases are those with ConsistencyDx greater than or equal to the threshold. Coverage denotes the proportion of all cases retained at each threshold. Values are shown as point estimates with 95% Wilson confidence intervals. Empty accuracy entries indicate that no cases were retained at that threshold, so accuracy was not estimable.

threshold	runs_N	accuracy	accuracy_95CI	coverage	coverage_95CI
0.5	3	0.913	[0.886, 0.934]	0.958	[0.938, 0.972]
0.5	5	0.927	[0.901, 0.946]	0.964	[0.945, 0.976]
0.5	10	0.914	[0.887, 0.935]	0.969	[0.951, 0.981]
0.55	3	0.926	[0.900, 0.946]	0.931	[0.907, 0.949]
0.55	5	0.938	[0.914, 0.956]	0.944	[0.921, 0.960]
0.55	10	0.929	[0.904, 0.948]	0.946	[0.923, 0.962]
0.6	3	0.939	[0.914, 0.957]	0.895	[0.866, 0.918]
0.6	5	0.943	[0.919, 0.960]	0.918	[0.892, 0.938]
0.6	10	0.937	[0.912, 0.955]	0.917	[0.890, 0.937]
0.65	3	0.945	[0.921, 0.962]	0.862	[0.831, 0.888]
0.65	5	0.948	[0.925, 0.965]	0.875	[0.845, 0.900]
0.65	10	0.948	[0.925, 0.965]	0.878	[0.848, 0.903]
0.7	3	0.952	[0.928, 0.968]	0.833	[0.800, 0.862]
0.7	5	0.963	[0.941, 0.977]	0.828	[0.794, 0.857]
0.7	10	0.957	[0.934, 0.972]	0.835	[0.802, 0.864]
0.75	3	0.962	[0.939, 0.976]	0.76	[0.723, 0.794]
0.75	5	0.969	[0.948, 0.982]	0.766	[0.729, 0.799]
0.75	10	0.965	[0.943, 0.979]	0.777	[0.740, 0.810]
0.8	3	0.969	[0.947, 0.982]	0.713	[0.674, 0.749]
0.8	5	0.974	[0.953, 0.986]	0.708	[0.668, 0.744]
0.8	10	0.967	[0.944, 0.980]	0.708	[0.668, 0.744]
0.85	3	0.974	[0.952, 0.986]	0.633	[0.592, 0.673]
0.85	5	0.974	[0.951, 0.986]	0.626	[0.585, 0.666]
0.85	10	0.977	[0.955, 0.988]	0.628	[0.587, 0.667]
0.9	3	0.975	[0.950, 0.988]	0.517	[0.476, 0.559]
0.9	5	0.989	[0.968, 0.996]	0.494	[0.452, 0.535]
0.9	10	0.996	[0.978, 0.999]	0.463	[0.422, 0.505]
0.95	3	0.981	[0.952, 0.993]	0.385	[0.345, 0.426]
0.95	5	0.994	[0.968, 0.999]	0.316	[0.278, 0.356]
0.95	10	0.994	[0.966, 0.999]	0.299	[0.263, 0.339]

Table S15 | Threshold-wise paired comparisons across repeated runs (N=3, 5, 10).

For each consistency threshold, p_consistency_friedman is from a Friedman test comparing per-case ConsistencyDx across N=3, 5, and 10 (same cases paired across runs). p_coverage_cochranQ is from Cochran's Q test comparing per-case binary coverage status (ConsistencyDx above threshold vs not) across N=3, 5, and 10. p_accuracy_cochranQ_on_covered_intersection is from Cochran's Q test comparing per-case binary correctness across N=3, 5, and 10, restricted to cases covered in all three runs (n_acc_complete). Missing p-values indicate non-estimable tests due to insufficient discordant information.

threshold	p_consistency_friedman	p_coverage_cochranQ	p_accuracy_cochranQ_on_covered_intersection
0.5	0.123	0.325	0.264
0.55	0.123	0.168	0.168
0.6	0.123	0.033	0.202
0.65	0.123	0.289	0.097
0.7	0.123	0.799	0.066
0.75	0.123	0.437	0.607
0.8	0.123	0.878	0.607
0.85	0.123	0.875	
0.9	0.123	0.002	
0.95	0.123	1.60E-08	

Table S16 | Threshold-stratified accuracy and coverage for consistency-based case retention across temperatures (Model = GLM-4.5-Air).

For each ConsistencyDx threshold, results are reported separately for T=0.01, 0.3, 0.6, and 0.9. Accuracy denotes the proportion of correct final decisions among retained cases, where retained cases are those with ConsistencyDx greater than or equal to the threshold. Coverage denotes the proportion of all cases retained at each threshold. Values are shown as point estimates with 95% Wilson confidence intervals. Empty accuracy entries indicate that no cases were retained at that threshold, so accuracy was not estimable.

threshold	T	accuracy	accuracy_95CI	coverage	coverage_95CI
0.5	0.01	0.927	[0.901, 0.946]	0.964	[0.945, 0.976]
0.55	0.01	0.938	[0.914, 0.956]	0.944	[0.921, 0.960]
0.6	0.01	0.943	[0.919, 0.960]	0.918	[0.892, 0.938]
0.65	0.01	0.948	[0.925, 0.965]	0.875	[0.845, 0.900]
0.7	0.01	0.963	[0.941, 0.977]	0.828	[0.794, 0.857]
0.7	0.3	0.957	[0.934, 0.972]	0.804	[0.769, 0.835]

0.7	0.6	0.96	[0.937, 0.974]	0.809	[0.775, 0.840]
0.7	0.9	0.965	[0.943, 0.979]	0.777	[0.740, 0.810]
0.75	0.01	0.969	[0.948, 0.982]	0.766	[0.729, 0.799]
0.75	0.3	0.966	[0.943, 0.980]	0.742	[0.704, 0.777]
0.75	0.6	0.973	[0.953, 0.985]	0.748	[0.710, 0.782]
0.75	0.9	0.975	[0.955, 0.986]	0.728	[0.689, 0.763]
0.8	0.01	0.974	[0.953, 0.986]	0.708	[0.668, 0.744]
0.8	0.3	0.976	[0.956, 0.987]	0.69	[0.650, 0.727]
0.8	0.6	0.979	[0.959, 0.989]	0.684	[0.644, 0.722]
0.8	0.9	0.981	[0.960, 0.991]	0.653	[0.613, 0.692]
0.85	0.01	0.974	[0.951, 0.986]	0.626	[0.585, 0.666]
0.85	0.3	0.988	[0.970, 0.995]	0.613	[0.572, 0.653]
0.85	0.6	0.982	[0.961, 0.992]	0.601	[0.559, 0.641]
0.85	0.9	0.983	[0.962, 0.993]	0.55	[0.508, 0.591]
0.9	0.01	0.989	[0.968, 0.996]	0.494	[0.452, 0.535]
0.9	0.3	0.993	[0.973, 0.998]	0.485	[0.443, 0.526]
0.9	0.6	0.992	[0.971, 0.998]	0.448	[0.407, 0.490]
0.9	0.9	0.987	[0.961, 0.995]	0.405	[0.365, 0.446]
0.95	0.01	0.994	[0.968, 0.999]	0.316	[0.278, 0.356]
0.95	0.3	0.994	[0.968, 0.999]	0.314	[0.277, 0.354]
0.95	0.6	0.994	[0.966, 0.999]	0.299	[0.263, 0.339]
0.95	0.9	0.985	[0.946, 0.996]	0.238	[0.204, 0.275]

Table S17 | Threshold-wise paired comparisons across temperatures (T=0.01, 0.3, 0.6, 0.9).

For each ConsistencyDx threshold, paired tests were performed across the same 551 cases. p_consistency_friedman is from a Friedman test comparing per-case ConsistencyDx across temperatures. p_coverage_cochranQ is from Cochran's Q test comparing per-case binary coverage status (ConsistencyDx greater than or equal to the threshold vs below threshold) across temperatures. p_accuracy_cochranQ_on_covered_intersection is from Cochran's Q test comparing per-case binary correctness across temperatures, restricted to cases retained at all temperatures. Missing p-values indicate non-estimable tests due to insufficient discordant information.

**threshold**	**p_consistency_friedman**	**p_coverage_cochranQ**	**p_accuracy_cochranQ_on_covered_intersection**
0.5	3.76E-12	0.028	0.502
0.55	3.76E-12	0.002	0.379
0.6	3.76E-12	0.001	0.410
0.65	3.76E-12	0.057	0.484

0.7	3.76E-12	0.013	0.392
0.75	3.76E-12	0.127	0.300
0.8	3.76E-12	0.013	
0.85	3.76E-12	2.07E-04	
0.9	3.76E-12	1.29E-05	
0.95	3.76E-12	3.31E-06	

Table S18 | Threshold-stratified operating characteristics across embedding models.

For each ConsistencyDx threshold, accuracy and coverage are reported separately for MiniLM, MPNet, and BGE embeddings. Accuracy denotes diagnostic accuracy among retained cases, where retained cases are those with ConsistencyDx greater than or equal to the threshold. Coverage denotes the proportion of all cases retained at each threshold. Values are shown as point estimates with two-sided 95% Wilson confidence intervals.

threshold	embed	accuracy	accuracy_95CI	coverage	coverage_95CI
0.5	MiniLM	0.927	[0.901, 0.946]	0.964	[0.945, 0.976]
0.5	bge	0.906	[0.878, 0.927]	1	[0.993, 1.000]
0.5	mpnet	0.92	[0.894, 0.940]	0.978	[0.962, 0.987]
0.55	MiniLM	0.938	[0.914, 0.956]	0.944	[0.921, 0.960]
0.55	bge	0.906	[0.878, 0.927]	1	[0.993, 1.000]
0.55	mpnet	0.926	[0.901, 0.946]	0.962	[0.942, 0.975]
0.6	MiniLM	0.943	[0.919, 0.960]	0.918	[0.892, 0.938]
0.6	bge	0.906	[0.878, 0.927]	1	[0.993, 1.000]
0.6	mpnet	0.934	[0.909, 0.952]	0.935	[0.911, 0.952]
0.65	MiniLM	0.948	[0.925, 0.965]	0.875	[0.845, 0.900]
0.65	bge	0.909	[0.882, 0.930]	0.996	[0.987, 0.999]
0.65	mpnet	0.944	[0.920, 0.961]	0.902	[0.874, 0.924]
0.7	MiniLM	0.963	[0.941, 0.977]	0.828	[0.794, 0.857]
0.7	bge	0.923	[0.898, 0.943]	0.971	[0.953, 0.982]
0.7	mpnet	0.951	[0.928, 0.967]	0.853	[0.821, 0.880]
0.75	MiniLM	0.969	[0.948, 0.982]	0.766	[0.729, 0.799]
0.75	bge	0.939	[0.915, 0.957]	0.927	[0.903, 0.946]
0.75	mpnet	0.968	[0.947, 0.981]	0.795	[0.759, 0.827]
0.8	MiniLM	0.974	[0.953, 0.986]	0.708	[0.668, 0.744]
0.8	bge	0.952	[0.928, 0.968]	0.862	[0.831, 0.888]
0.8	mpnet	0.975	[0.955, 0.986]	0.726	[0.687, 0.762]
0.85	MiniLM	0.974	[0.951, 0.986]	0.626	[0.585, 0.666]
0.85	bge	0.968	[0.946, 0.981]	0.739	[0.700, 0.774]
0.85	mpnet	0.974	[0.952, 0.986]	0.637	[0.596, 0.676]
0.9	MiniLM	0.989	[0.968, 0.996]	0.494	[0.452, 0.535]

0.9	bge	0.984	[0.963, 0.993]	0.559	[0.517, 0.600]
0.9	mpnet	0.982	[0.958, 0.992]	0.503	[0.461, 0.544]
0.95	MiniLM	0.994	[0.968, 0.999]	0.316	[0.278, 0.356]
0.95	bge	0.995	[0.972, 0.999]	0.354	[0.315, 0.395]
0.95	mpnet	0.994	[0.966, 0.999]	0.292	[0.256, 0.332]

Table S19 | Paired statistical comparisons of consistency-based selective prediction across embedding models.

For each ConsistencyDx threshold, paired tests were performed across the same 551 cases using MiniLM, MPNet, and BGE embeddings. p_consistency_friedman is from a Friedman test comparing per-case ConsistencyDx scores across embedding models. p_coverage_cochranQ is from Cochran's Q test comparing per-case binary coverage status (ConsistencyDx greater than or equal to the threshold vs below threshold) across embedding models. p_accuracy_cochranQ_on_covered_intersection is from Cochran's Q test comparing per-case binary correctness across embedding models, restricted to cases retained by all three embeddings. Missing p-values indicate non-estimable tests due to insufficient discordant information.

threshold	p_consistency_friedman	p_coverage_cochranQ	p_accuracy_cochranQ_on_covered_intersection
0.5	6.89E-28	9.97E-07	
0.55	6.89E-28	1.31E-10	
0.6	6.89E-28	4.07E-16	
0.65	6.89E-28	2.05E-24	
0.7	6.89E-28	1.15E-29	
0.75	6.89E-28	1.34E-31	
0.8	6.89E-28	5.69E-32	
0.85	6.89E-28	1.64E-20	
0.9	6.89E-28	1.63E-07	
0.95	6.89E-28	3.32E-06	

Table S20 | Five Run Token Overhead Ratios

group_label	temperature	doctor_five_vs_single_medianRatio	total_five_vs_single_medianRatio
GPT-OSS	T=0.01	5.27	5.28
GPT-OSS	T=0.3	5.23	5.26
GPT-OSS	T=0.6	5.11	5.15
GPT-OSS	T=0.9	5.21	5.27
GLM-4.5-Air	T=0.01	5.26	5.35

GLM-4.5-Air	T=0.3	5.27	5.29
GLM-4.5-Air	T=0.6	5.26	5.32
GLM-4.5-Air	T=0.9	5.29	5.29
GLM-5	T=0.01	5.05	5.05
GLM-5	T=0.3	5.04	5.09
GLM-5	T=0.6	5.08	5.07
GLM-5	T=0.9	5.06	5.1
Qwen-3.5	T=0.01	5.11	5.09
Qwen-3.5	T=0.3	5.11	5.07
Qwen-3.5	T=0.6	5.09	5.12
Qwen-3.5	T=0.9	5.05	5.03

Table S21 | Run Token By Model Family

**group_label**	**run_setting**	**doctor_tokens_median_k**	**doctor_tokens_iqr_k**	**doctor_tokens_mean_k**	**total_tokens_median_k**	**total_tokens_iqr_k**	**total_tokens_mean_k**	**doctor_share_of_total_median_pct**	**doctor_share_of_total_mean_pct**
GPT-OSS	Single run per encounter	177.5	131.2-246.6	202.5	192.1	142.8-266.5	219.3	92.4	92.4
GPT-OSS	Five-run per case	926.4	762.9-1170.6	1012.7	1010.9	834.4-1263.3	1096.3	91.6	92.4
GLM-4.5-Air	Single run per encounter	118.7	93.2-153.6	131.3	128.8	99.9-171.1	143.4	92.2	91.6
GLM-4.5-Air	Five-run per case	624.4	538.6-737.8	656.4	684.6	584.9-808.3	716.9	91.2	91.6
GLM-5	Single run per encounter	109.1	88.5-130.2	111.2	122.9	97.5-148.8	125.7	88.8	88.5
GLM-5	Five-run per case	551.5	494.3-610.6	556	623.7	556.7-693.5	628.4	88.4	88.5
Qwen-3.5	Single run per encounter	122.9	100.9-145.5	124.6	139.4	113.4-165.8	141	88.2	88.4
Qwen-3.5	Five-run per case	625.8	555.0-687.7	622.9	706.5	630.4-776.9	705.1	88.6	88.4

Table S22 | Token Run By Experiment

**group_label**	**temperature**	**run_setting**	**doctor_tokens_median_k**	**doctor_tokens_iqr_k**	**doctor_tokens_mean_k**	**total_tokens_median_k**	**total_tokens_iqr_k**	**total_tokens_mean_k**	**doctor_share_of_total_median_pct**	**doctor_share_of_total_mean_pct**
GPT-OSS	T=0.01	Single run per encounter	179.6	133.9-249.7	206.2	195.4	145.5-268.7	223.3	91.9	92.3
GPT-OSS	T=0.01	Five-run per case	946.7	777.2-1193.9	1030.9	1031.5	846.1-1292.2	1116.7	91.8	92.3

GPT-OSS	T=0.3	Single run per encounter	177.5	132.4-246.6	202.7	192.1	145.1-264.7	219.5	92.4	92.3
GPT-OSS	T=0.3	Five-run per case	928.7	761.1-1168.5	1013.4	1011.3	832.9-1258.8	1097.6	91.8	92.3
GPT-OSS	T=0.6	Single run per encounter	175.6	129.5-239.9	198.7	190.3	140.9-260.3	214.7	92.3	92.6
GPT-OSS	T=0.6	Five-run per case	897	752.5-1149.8	993.7	980.8	821.8-1243.9	1073.6	91.5	92.6
GPT-OSS	T=0.9	Single run per encounter	177.4	128.2-248.8	202.6	192	139.2-271.0	219.5	92.4	92.3
GPT-OSS	T=0.9	Five-run per case	924	764.0-1160.7	1012.9	1010.8	835.9-1249.9	1097.3	91.4	92.3
GLM-4.5-Air	T=0.01	Single run per encounter	116	90.9-150.9	129.6	126.1	96.7-168.0	141.4	92.1	91.7
GLM-4.5-Air	T=0.01	Five-run per case	610.1	522.5-722.1	647.9	673.9	566.4-789.6	706.8	90.5	91.7
GLM-4.5-Air	T=0.3	Single run per encounter	116.2	90.8-150.7	129.1	126.1	97.2-167.5	140.9	92.1	91.6
GLM-4.5-Air	T=0.3	Five-run per case	612.9	520.9-729.9	645.3	667.5	569.7-801.9	704.3	91.8	91.6
GLM-4.5-Air	T=0.6	Single run per encounter	118.2	93.8-150.8	129.6	127.7	100.5-168.5	141.6	92.6	91.5
GLM-4.5-Air	T=0.6	Five-run per case	621.8	535.7-723.1	647.8	679.5	580.4-792.5	707.9	91.5	91.5
GLM-4.5-Air	T=0.9	Single run per encounter	124	97.9-161.1	137	135.2	105.1-178.6	149.7	91.7	91.5
GLM-4.5-Air	T=0.9	Five-run per case	655.7	564.6-761.2	684.8	714.7	615.9-837.4	748.5	91.7	91.5
GLM-5	T=0.01	Single run per encounter	108.6	87.9-129.8	110.8	122.6	96.9-147.8	125.2	88.6	88.5
GLM-5	T=0.01	Five-run per case	548.3	494.0-607.0	553.8	619.2	557.2-690.2	626.1	88.5	88.5
GLM-5	T=0.3	Single run per encounter	109.2	87.1-130.7	111	123.1	96.4-149.5	125.6	88.7	88.4
GLM-5	T=0.3	Five-run per case	550.1	493.5-612.6	554.9	627.2	556.6-694.8	627.6	87.7	88.4
GLM-5	T=0.6	Single run per encounter	108	87.7-129.2	110.6	121.3	96.6-147.8	124.8	89.1	88.6
GLM-5	T=0.6	Five-run per case	548.5	490.8-604.8	553	614.8	549.8-684.2	624.1	89.2	88.6
GLM-5	T=0.9	Single run per encounter	110.4	90.6-130.6	112.4	124.6	100.3-150.0	127.2	88.6	88.4
GLM-5	T=0.9	Five-run per case	558.5	500.1-618.0	562.1	635.6	561.0-702.8	635.9	87.9	88.4

Qwen-3.5	T=0.01	Single run per encounter	123.7	102.3-145.0	125.5	140.4	115.3-165.7	142.3	88.1	88.2
Qwen-3.5	T=0.01	Five-run per case	631.9	559.0-694.3	627.6	715	632.0-787.8	711.3	88.4	88.2
Qwen-3.5	T=0.3	Single run per encounter	120.7	98.7-143.6	122.5	137.3	112.1-163.7	138.9	87.9	88.2
Qwen-3.5	T=0.3	Five-run per case	617.2	548.4-675.1	612.7	696.8	622.7-763.1	694.5	88.6	88.2
Qwen-3.5	T=0.6	Single run per encounter	124.3	102.4-147.2	126.2	140.2	114.8-167.2	142.6	88.6	88.5
Qwen-3.5	T=0.6	Five-run per case	632.6	557.4-694.2	631.1	717.6	633.1-783.4	712.8	88.1	88.5
Qwen-3.5	T=0.9	Single run per encounter	122.9	99.2-146.6	124.1	139.5	111.3-166.9	140.4	88.1	88.4
Qwen-3.5	T=0.9	Five-run per case	620.7	550.9-681.5	620.3	701.2	625.1-771.0	701.7	88.5	88.4

Table S23 | Controlled subset wall-clock runtime by deployed model configuration.

Single-run encounter-level wall-clock runtime was measured in a controlled subset of 35 encounters per model, consisting of five randomly sampled cases from each of seven disease cohorts. The same subset was used for all deployed model configurations. Runtime was measured from simulation start to completion and includes the doctor agent, patient simulator, tool calls, and workflow overhead. Values are reported as mean, standard deviation (SD), median, interquartile range (IQR), minimum, and maximum. Estimated five-run consistency runtime was calculated as five times the measured single-run runtime under a serial execution assumption.

model	singleRun_latency_mean_s	singleRun_latency_sd_s	singleRun_latency_median_s	singleRun_latency_q1_s	singleRun_latency_q3_s	singleRun_latency_min_s	singleRun_latency_max_s
GLM-4.5-Air	40.8	10.7	38.4	32.7	47.3	27.1	78.0
GLM-5	117.3	30.7	112.2	94.0	140.8	69.8	205.1
GPT-OSS	152.3	80.8	125.6	100.2	189.2	59.8	373.2
Qwen-3.5	117.2	60.7	96.0	70.3	149.5	55.3	295.3

Table S24 | Open-weight Model Screening

Model	Failure mode	Status
Llama-3.3-70B-Instruct	Skipped dialogue;<br>malformed JSON for structured arguments;	Excluded

Kimi K2.5	vLLM tool-parser incompatibility at time of experiments	Excluded
GLM-4.5V	Context-length limit exceeded in multi-turn encounters	Excluded
Gemma-4-31B	malformed JSON for structured arguments	Excluded
GLM-4.5-Air-FP8	–	Selected (primary)
GPT-OSS-120B*	–	Evaluated (revision)
GLM-5*	–	Evaluated (revision)
Qwen3.5-397B*	–	Evaluated (revision)

* Models added during manuscript revision to address reviewer concerns regarding generalizability across architectures.

Table S25 | System Prompt for the Patient Agent on MIMIC-based Benchmarks.

This prompt is adapted from MIRA[29].

You are simulating a patient in the emergency department of Beth Medical Center in Boston. Your {primary_symptom}

Below, you have been provided with a summary of the clinical history that gives a brief description of your symptoms.
This patient history is based on a real-world hospital stay, and may contain information that is only generated **after** the situation you are simulating (during the hospital stay).
In such a case, ignore the information from the hospital stay including the procedures, treatments and diagnoses.
Important note: In case the initial information (provided to you below as 'Clinical History Summary') contains information on your hospital stay (that happens after the situation you simulate now), ignore it and never reveal it to the doctor.
For instance, there might be information on procedures in the emergency department ("in the ED ...") that you should not reveal to the doctor.

- Behave and speak as a real patient would.
- Respond only with information from the clinical history summary. Do not add any new information, symptoms, findings, or medications that are not mentioned; assume they are absent.
- For instance, if asked about medication details not specified (e.g., dosage), inform the doctor that you do not know.
- As another example, if questioned about a symptom not included in the summary, state that you do not have that symptom.
- Ignore any placeholders like “___” in the clinical history summary.
- If asked closed questions, only answer the question.
- If asked open questions, respond with 1-3 sentences, not telling all the information at once.
- Strictly adhere to the information provided below.
- Speak in simple terms, as a layman would - without medical jargon (but provide all information you have).
- If you are asked about your current medication, respond with the admission medication provided below. If you are provided with the string 'No current medication.' or 'None'or something similar, state that you are not taking any medication at the moment.

- If you receive information like this: 'The Preadmission Medication list may be inaccurate and requires further investigation.' or similar, ignore this information. Take the provided medication as ground truth.
- If you have information on the dosage and frequency of each medication, include it in your response - if not, leave it out.

In the course of the conversation, the doctor will inform you about the results of the diagnostic tests (lab results, imaging like CT or ultrasound, etc.) that you have been through and any further next steps in diagnosis and treatments.
Please confirm if you understand and are ready to continue.

Your 'Clinical History Summary':
{anamnesis_summary}

Table S26 | System Prompt for the Patient Agent on VivaBench.

You are simulating a patient in the emergency department of a Medical Center. Your {primary_symptom}

Your job is to answer the doctor's questions truthfully using only the structured case facts provided below.

Rules:
- Stay in character as the patient.
- Do not reveal the final diagnosis unless the doctor explicitly shares it first.
- Do not volunteer all findings at once. Answer only what the doctor asks.
- Use simple patient-facing language.
- If you are asked about information not present in the case, say you do not know or that it was not mentioned.
- Do not reveal imaging, blood, or microbiology results unless the doctor has already ordered and discussed them.

Structured case summary:
{anamnesis_summary}

Table S27 | System Prompt for the Physician Agent on MIMIC-based Benchmarks.

This prompt is adapted from MIRA[29].

You are a medical superintelligence. Engage in a conversational interaction with a patient to comprehensively complete their case from clinical history, through diagnostics, to treatment within an emergency department setting. You will have access to tools equivalent to a medical doctor to gather information and make decisions.
Once you have completed all diagnostic steps and selected all relevant treatment options, like requesting medication or a surgical procedure, explain it to the patient and only once you have finished explaining or answered their questions, finish the case using the `admission` action.

Steps
1. **Detailed Clinical History (Medical History & Interview):**
- Obtain a detailed medical history from the patient, including current symptoms, past medical history, family history, medication use, allergies, and lifestyle factors.

- **Ask focused questions, maximum of 2-3 at a time**, and wait for the patient`s response before asking the next question.
- Begin with open-ended questions to allow the patient to describe their concerns and symptoms in their own words.
- Clarify and elaborate with targeted questions to fill in details and ensure a complete understanding of the patient`s condition.
- Only once you have completed the complete clinical history, begin the diagnostic process.

2. **Diagnostic Tools & Actions:**
  - Use diagnostic tools to gather further information as needed. This may include requesting lab tests, imaging, or other diagnostic procedures.
  - **Use tools with the highest diagnostic accuracy and the highest likelihood of providing relevant information for the current patient condition.**
    - Example: CT Chest is more accurate than a Chest X-ray. Choose the right imaging for the potential diagnosis.
  - Continually assess information obtained from these tools to refine your understanding of the patient`s condition.
  - Explain what you are doing to the patient.
  - **Rules for Requesting Tests and Handling Responses:**
    - **Check History First:** Before requesting ANY diagnostic test, you MUST first review the entire conversation history to ensure you are not making a duplicate request. A request is considered a duplicate if you have already received a result for it OR if you have been told the result is unavailable.
    - **Handling a "Data Not Available" Response:** If the system returns a message like "No lab result available" or "result unavailable or unmeasurable", you MUST treat this as a final answer for that test.
      Action: Acknowledge this limitation and make your clinical decisions based on the information you currently have.
      Prohibition: DO NOT under any circumstances request that same test again.
    - **Handling an "Execution Error" Response:** If the system returns an error message caused by your own request format (e.g., "Invalid JSON input"), this is a correctable mistake.
      Action: You MUST analyze the error message, fix the format of your tool call, and then **retry the request exactly once.**

3. **Decide on Treatment:**
  - Implement medical treatments, prescribing medication, or / and recommending surgical procedures based on the findings from the clinical history and diagnostic results.
  - Before calling `prescribe_medication`, you MUST first review the conversation history. NEVER prescribe a medication that has already been prescribed in a previous step.
    - **Comprehensive Medication Management**: Your `prescribe_medication` call MUST be comprehensive.
      - **Include ALL new medications** required to treat the diagnosis and its complications (e.g., antibiotics for infection, potassium replacement for hypokalemia).
      - **Include ALL pre-existing medications** that the patient should continue taking.
      - **Explicitly state any medications to be paused or stopped**, with a brief reason (e.g., "Pause Metformin due to acute kidney injury").
  - If you want to perform a procedure, first call the `search_procedure` tool to search for the procedure and receive a list of up to 10 options that you can call the `request_procedure` tool with.

4. **Finish:**
 - Before finishing, ensure that you have completed all actions required.
 - Before finishing, ensure that you have uploaded *all* relevant medication (new medication and medication that the patient is already taking (eventually paused)).
 - Once you have completed all diagnostic steps and selected all relevant treatment options, like requesting medication or a surgical procedure, explain it to the patient and only once you have finished explaining or answered their questions, finish the case using the `admission` action.

Output Format
At each step, briefly explain your actions and thoughts in the conversation to the patient, and then present the conclusion with the decided treatment plan.

Notes
- You must communicate everything you do to the patient.
- Ensure all interactions are patient-centric, maintaining a professional and empathetic tone.
- Incorporate all gathered information efficiently to determine the most appropriate course of action.
- Adapt to changes in patient status or new information, adjusting the actions as necessary.
- Communicate all actions to the patient before finishing the case through the `admission` action.

Table S28 | System Prompt for the Physician Agent on VivaBench.

You are a medical superintelligence. Engage in a conversational interaction with a patient to comprehensively complete their case from clinical history to diagnostics. You will have access to tools equivalent to a medical doctor to gather information and make decisions.

Rules:
- Follow a staged clinical workflow: review, provisional assessment, investigation, final diagnosis.
- Start with clinical history taking and physical examination before ordering any investigations.
- Ask one or two focused questions at a time.
- During the review stage, use conversation and `request_physical_exam` to understand the presentation.
- Before beginning investigations, form a provisional differential internally and explain your next diagnostic step to the patient.
- Once you start ordering investigations, do not go back to broad history taking unless absolutely required to clarify a specific test result.
- Order only targeted investigations that would support, narrow, or rule out your leading diagnoses.
- Do not finalize the case based on history or physical examination alone if additional available investigations would materially affect the diagnosis.
- Do not request the same unavailable or already-returned test again.
- Explain your actions to the patient in plain language.
- When sufficient diagnostic evidence has been gathered, provide your final diagnosis and call `finish`.

Available tools in this benchmark may include:
- `request_physical_exam`
- `request_blood_test`
- `request_urine_test`
- `request_bedside_test`
- `requestRadiology`
- `request_microbiology`
- `request_other_investigation`
- `finish`

Tool guidance:
- Use `request_blood_test` for blood and blood-adjacent laboratory studies.
- Use `request_urine_test` for urinalysis, urine microscopy, and urine culture requests.
- Use `request_bedside_test` for ECG, point-of-care, or bedside physiological studies.
- Use `requestRadiology` only for imaging performed by radiology services.
- Use `request_microbiology` for cultures, PCR, serology, and infectious microbiology studies.
- Use `request_other_investigation` for specialized studies that are not blood, urine, microbiology, radiology, or bedside tests, such as peripheral smear, tissue, CSF, genetic, or other fluid investigations.

Your goal is accurate, disciplined clinical reasoning under benchmark constraints, not maximal test ordering.

Table S29 | System Prompt for the Differential Diagnosis (DDx) Critic Agent.

You are a senior clinical expert specializing in diagnostic safety and risk mitigation. You do NOT interact with patients or execute external tools.<br>Your sole purpose is to act as a "devil's advocate" to critique a provided differential diagnosis.<br>Your task is to analyze the patient summary and the proposed diagnoses and provide a critical analysis.<br>Your output MUST be a structured critique that includes:<br>1. **Arguments Against**: For each proposed diagnosis, provide the strongest counter-argument or point out missing evidence.<br>2. **"Don't Miss" Diagnoses**: For the most likely diagnosis, identify critical, high-risk alternative diagnoses that share similar symptoms but have not been considered.<br>3. **Revised Plan**: Suggest specific actions (e.g., additional tests) to address the weaknesses you've identified.<br>Your response should be concise, critical, and focused on improving diagnostic safety.

Table S30 | System Prompt for the Patient Agent (Perturbed Condition).

You are simulating a patient in the emergency department of Beth Medical Center in Boston.<br>- Behave and speak as a real patient would.<br>- If asked closed questions, only answer the question.<br>- If asked open questions, respond with 1-3 sentences, not telling all the information at once.<br>- Strictly adhere to the information provided below.<br>- Speak in simple terms, as a layman would - without medical jargon.<br><br>In the course of the conversation, the doctor will inform you about the results of the diagnostic tests (lab results, imaging like CT or ultrasound, etc.) that you have been through and any further next steps in diagnosis and treatments.<br>Please confirm if you understand and are ready to continue.

Table S31 | System Prompt for the LLM evaluator for the MIMIC-IV-derived benchmarks.

This prompt is adapted from MIRA[29].

You are a medical expert. You will be provided with a ground truth diagnosis and an assistant's diagnosis.<br>These can be on different levels of specificity. Therefore you also receive a matching criterion.<br>Your task is to determine if the assistant's diagnosis matches the ground truth diagnosis based on the matching criterion.

Respond with the given json schema, providing a reasoning for your answer and a boolean decision (True if they match, False otherwise).
Mostly consider the overall 'Matching criterion', not any specific details.
Decide false if the ground truth and assistant diagnoses conflict with each other.

Example 1:
Ground Truth: 'Appendicitis'
Assistant: 'Complicated appendicitis with local peritonitis and perforation'
Matching criterion: 'appendicitis'
Decision: 'True'
Example 2:
Ground Truth: 'Appendicitis'
Assistant: 'Appendicitis with local peritonitis'
Matching criterion: 'appendicitis'
Decision: 'True'
Example 3:
Ground Truth: 'Acute appendicitis with appendicolith'
Assistant: 'Appendicitis without local peritonitis'
Matching criterion: 'appendicitis'
Decision: 'True'
Example 4:
Ground Truth: 'Acute cholecystitis'
Assistant: 'Appendicitis with local peritonitis'
Matching criterion: 'cholecystitis'
Decision: 'False'
Example 5:
Ground Truth: 'Acute cholecystitis'
Assistant: 'Choledocholithiasis with possible cholecystitis'
Matching criterion: 'cholecystitis'
Decision: 'True'
Example 6:
Ground Truth: 'other pulmonary embolism and infarction'
Assistant: 'massive pulmonary embolism with right ventricular strain'
Matching criterion: 'Pulmonary Embolism'
Decision: 'True'
Example 7:
Ground Truth: 'Sepsis, unspecified organism'
Assistant: 'Diverticulitis with perforation'
Matching criterion: 'diverticulitis'
Decision: 'True'

Ground Truth: {ground_truth}
Assistant: {assistant_diag}
Matching criterion: {self.criterion}

First, analyze the relationship between the ground truth diagnosis, the assistant's diagnosis, and the matching criterion. Explain relevant clinical reasoning.
Then, output your judgment in the following strict JSON format:
```json
{{
    "reasoning": "Explain in clear and concise clinical logic why the diagnoses match or not, in one paragraph.",
"decision": true  // or false
}}
```

Table S32 | System Prompt for the LLM evaluator for VivaBench.

```
You are a medical expert evaluating a benchmark diagnosis.

    You will be given:
    - gold diagnoses for the case
    - accepted differential diagnoses
    - the assistant's final diagnosis

    The case may have multiple correct diagnoses.

    Your task is to judge whether the assistant's diagnosis is:
    - fully correct,
    - partially correct,
    - or incorrect.

    Rules:
      - Set "decision" to true only if the assistant identifies all clinically central gold diagnoses, or gives an equivalent diagnosis that correctly covers them, without adding a major contradictory diagnosis.
      - Set "partial_credit" to true if the assistant identifies at least one important gold diagnosis, or a closely related accepted differential, but misses one or more important gold diagnoses.
      - If the assistant identifies only an accepted differential and none of the gold diagnoses, it cannot be fully correct.
      - If the assistant diagnosis is clinically unrelated to the gold diagnoses, set both "decision" and "partial_credit" to false.
    - If the assistant includes a major contradictory diagnosis, set "decision" to false.
    - List any important missed gold diagnoses in "missed_gold_diagnoses".
    - Use clinical meaning, not exact wording.

    Examples:
    Example 1:
        Gold diagnoses: ["Legionnaires' disease (Legionella pneumonia)", "Rhabdomyolysis", "Acute kidney failure"]
    Accepted differentials: []
    Assistant diagnosis: "Legionnaires' disease, rhabdomyolysis, and acute kidney injury"
    Output:
    ```json
    {{
       "reasoning": "The assistant captures all major gold diagnoses with clinically equivalent wording. Acute kidney injury is an acceptable wording variant for acute kidney failure, so the answer is overall correct.",
      "decision": true,
      "partial_credit": false,
      "missed_gold_diagnoses": []
    }}
    ```

    Example 2:
        Gold diagnoses: ["Legionnaires' disease (Legionella pneumonia)", "Rhabdomyolysis", "Acute kidney failure"]
    Accepted differentials: []
    Assistant diagnosis: "Legionnaires' disease"
    Output:
    ```json
    {{
```
```

````
    "reasoning": "The assistant correctly identifies one important gold diagnosis but misses the associated rhabdomyolysis and acute kidney failure. The answer is incomplete rather than fully correct.",
    "decision": false,
    "partial_credit": true,
    "missed_gold_diagnoses": ["Rhabdomyolysis", "Acute kidney failure"]
  }}
  ```

  Example 3:
  Gold diagnoses: ["New-onset thyrotoxicosis"]
  Accepted differentials: ["Acalculous cholecystitis"]
  Assistant diagnosis: "Acalculous cholecystitis"
  Output:
  ```json
  {{
    "reasoning": "The assistant names only an accepted differential and fails to identify the gold diagnosis of new-onset thyrotoxicosis. The answer is not overall correct, but it is clinically related enough to merit partial credit.",
    "decision": false,
    "partial_credit": true,
    "missed_gold_diagnoses": ["New-onset thyrotoxicosis"]
  }}
  ```

  Example 4:
  Gold diagnoses: ["Giant cell arteritis (temporal arteritis)"]
  Accepted differentials: []
  Assistant diagnosis: "Parapneumonic effusion secondary to pneumonia"
  Output:
  ```json
  {{
    "reasoning": "The assistant provides a diagnosis that is clinically unrelated to the gold diagnosis of giant cell arteritis. The gold diagnosis is entirely missed and the answer is incorrect.",
    "decision": false,
    "partial_credit": false,
    "missed_gold_diagnoses": ["Giant cell arteritis (temporal arteritis)"]
  }}
  ```

  Example 5:
  Gold diagnoses: ["Acute pulmonary embolism", "Right heart strain"]
  Accepted differentials: []
  Assistant diagnosis: "Pulmonary embolism"
  Output:
  ```json
  {{
    "reasoning": "The assistant identifies the main disease process of pulmonary embolism but misses the associated right heart strain, which is an important gold diagnosis. The answer is clinically meaningful but incomplete.",
    "decision": false,
    "partial_credit": true,
    "missed_gold_diagnoses": ["Right heart strain"]
  }}
  ```
````
````

```
Example 6:
Gold diagnoses: ["Acute pancreatitis"]
Accepted differentials: ["Biliary colic"]
Assistant diagnosis: "Acute pancreatitis with acute appendicitis"
Output:
```json
{{
  "reasoning": "The assistant identifies the gold diagnosis of acute pancreatitis, but also adds acute appendicitis, which is a major contradictory diagnosis not supported by the gold labels. Therefore the answer is not fully correct, though it still captures an important gold diagnosis.",
  "decision": false,
  "partial_credit": true,
  "missed_gold_diagnoses": []
}}
```

Gold diagnoses: {json.dumps(gold_diagnoses, ensure_ascii=False)}
Accepted differentials: {json.dumps(gold_differentials, ensure_ascii=False)}
Assistant diagnosis: {assistant_diag}

Return strict JSON only:
```json
{{
  "reasoning": "Explain briefly why the assistant is correct, partially correct, or incorrect.",
  "decision": true,
  "partial_credit": false,
  "missed_gold_diagnoses": []
}}
```
```

Table S33 | LingCertDx AUC across different versions of the hedging lexicon.

**Lexicon version**	**LingCertDx AUC [95% CI]**	**LingCertR AUC [95% CI]**
Original (compact)	0.633 [0.567, 0.702]	0.705 [0.629, 0.778]
Broad (inclusive, unfiltered)	0.632 [0.566, 0.701]	0.594 [0.515, 0.670]
Refined (curated)	0.635 [0.569, 0.705]	0.789 [0.727, 0.840]

Table S34 | Cross-model ROC AUC summary for MIRA-v2 confidence analyses.

AUC for the evaluated confidence and reliability-related metrics on Qwen-3.5, GLM-5 and GPT-OSS under baseline conditions on MIRA-v2, reported for the overall cohort and separately for the diagnostic output and reasoning trace. Lower and upper bounds indicate bootstrapped 95% confidence intervals based on 2,000 bootstrap iterations. Across all three additional models, ConsistencyDx remained the strongest evaluated metric in the diagnostic stream.

Model	Stream	Metric	AUC	95%CI
Qwen-3.5	Diagnosis	ConceptDensityDx	0.707	[0.653,0.756]
Qwen-3.5	Diagnosis	ConsistencyDx	0.852	[0.795,0.902]
Qwen-3.5	Diagnosis	LingCertDx	0.589	[0.532,0.648]
Qwen-3.5	Diagnosis	ProbScoreDx	0.662	[0.583,0.736]
Qwen-3.5	Reasoning	ConceptDensityR	0.445	[0.369,0.519]
Qwen-3.5	Reasoning	ConsistencyR	0.700	[0.620,0.775]
Qwen-3.5	Reasoning	LingCertR	0.702	[0.636,0.762]
Qwen-3.5	Reasoning	ProbScoreR	0.675	[0.601,0.743]
GLM-5	Diagnosis	ConceptDensityDx	0.640	[0.577,0.699]
GLM-5	Diagnosis	ConsistencyDx	0.834	[0.774,0.889]
GLM-5	Diagnosis	LingCertDx	0.590	[0.527,0.666]
GLM-5	Diagnosis	ProbScoreDx	0.539	[0.446,0.635]
GLM-5	Reasoning	ConceptDensityR	0.449	[0.373,0.522]
GLM-5	Reasoning	ConsistencyR	0.699	[0.614,0.777]
GLM-5	Reasoning	LingCertR	0.682	[0.615,0.754]
GLM-5	Reasoning	ProbScoreR	0.715	[0.640,0.786]
GPT-OSS	Diagnosis	ConceptDensityDx	0.695	[0.645,0.743]
GPT-OSS	Diagnosis	ConsistencyDx	0.842	[0.796,0.882]
GPT-OSS	Diagnosis	LingCertDx	0.615	[0.559,0.672]
GPT-OSS	Diagnosis	ProbScoreDx	0.735	[0.682,0.789]
GPT-OSS	Reasoning	ConceptDensityR	0.582	[0.513,0.650]
GPT-OSS	Reasoning	ConsistencyR	0.803	[0.746,0.853]
GPT-OSS	Reasoning	LingCertR	0.752	[0.697,0.805]
GPT-OSS	Reasoning	ProbScoreR	0.478	[0.448,0.503]

Table S35 | Review-fraction and residual-error operating points for ConsistencyDx on MIRA-v2.

Threshold-sweep analysis showing retained cases, coverage, deferred cases, review fraction, residual autonomous-stream errors, deferred-stream errors, and retained accuracy across ConsistencyDx thresholds under baseline conditions on MIRA-v2. This analysis is descriptive and was used to characterize how residual benchmark-defined autonomous failures decline as review becomes more conservative; it was not used to define a deployment guarantee of zero-risk autonomy.

Threshold	Retained n	Coverage	Deferred n	Review fraction	Autonomous errors	Deferred errors	Retained accuracy
0.5	531	0.964	20	0.036	39	13	0.927
0.55	520	0.944	31	0.056	32	20	0.938
0.6	506	0.918	45	0.082	29	23	0.943

0.65	482	0.875	69	0.125	25	27	0.948
0.7	456	0.828	95	0.172	17	35	0.963
0.75	422	0.766	129	0.234	13	39	0.969
0.8	390	0.708	161	0.292	10	42	0.974
0.85	345	0.626	206	0.374	9	43	0.974
0.9	272	0.494	279	0.506	3	49	0.989
0.95	174	0.316	377	0.684	1	51	0.994
1	99	0.180	452	0.820	1	51	0.990

Table S36 | Cross-model accuracy–coverage operating points for ConsistencyDx on MIRA-v2.

Coverage was defined as the proportion of cases retained above the specified ConsistencyDx threshold. Retained accuracy and coverage are reported for Qwen-3.5, GLM-5 and GPT-OSS under baseline conditions on MIRA-v2, with case-level 95% confidence intervals computed using the Wilson score interval. Threshold analyses are descriptive and were not used to optimize a deployment threshold on an independent validation set.

Model	Threshold	Accuracy	Accuracy_CI	Coverage	Coverage_CI
Qwen-3.5	0.5	0.923	[0.898,0.943]	0.971	[0.953,0.982]
Qwen-3.5	0.55	0.926	[0.901,0.946]	0.962	[0.942,0.975]
Qwen-3.5	0.6	0.929	[0.904,0.948]	0.949	[0.928,0.965]
Qwen-3.5	0.65	0.934	[0.909,0.952]	0.935	[0.911,0.952]
Qwen-3.5	0.7	0.938	[0.913,0.956]	0.902	[0.874,0.924]
Qwen-3.5	0.75	0.952	[0.928,0.968]	0.862	[0.831,0.888]
Qwen-3.5	0.8	0.962	[0.940,0.976]	0.809	[0.775,0.840]
Qwen-3.5	0.85	0.968	[0.946,0.981]	0.733	[0.695,0.768]
Qwen-3.5	0.9	0.98	[0.959,0.990]	0.632	[0.591,0.671]
Qwen-3.5	0.95	0.985	[0.962,0.994]	0.488	[0.447,0.530]
Qwen-3.5	1			0	[0.000,0.007]
GLM-5	0.5	0.926	[0.901,0.946]	0.962	[0.942,0.975]
GLM-5	0.55	0.928	[0.902,0.947]	0.953	[0.932,0.968]
GLM-5	0.6	0.926	[0.901,0.946]	0.938	[0.915,0.956]
GLM-5	0.65	0.933	[0.908,0.952]	0.920	[0.894,0.940]
GLM-5	0.7	0.948	[0.925,0.965]	0.878	[0.848,0.903]
GLM-5	0.75	0.957	[0.934,0.972]	0.840	[0.807,0.869]
GLM-5	0.8	0.959	[0.936,0.974]	0.795	[0.759,0.827]
GLM-5	0.85	0.979	[0.960,0.989]	0.699	[0.659,0.736]
GLM-5	0.9	0.981	[0.959,0.991]	0.572	[0.530,0.612]
GLM-5	0.95	0.979	[0.951,0.991]	0.423	[0.382,0.465]
GLM-5	1			0	[0.000,0.007]

GPT-OSS	0.5	0.881	[0.850,0.906]	0.958	[0.938,0.972]
GPT-OSS	0.55	0.892	[0.862,0.916]	0.942	[0.919,0.959]
GPT-OSS	0.6	0.905	[0.876,0.927]	0.913	[0.886,0.934]
GPT-OSS	0.65	0.911	[0.882,0.933]	0.877	[0.846,0.901]
GPT-OSS	0.7	0.921	[0.893,0.943]	0.829	[0.796,0.859]
GPT-OSS	0.75	0.934	[0.906,0.954]	0.771	[0.734,0.804]
GPT-OSS	0.8	0.946	[0.919,0.965]	0.710	[0.670,0.746]
GPT-OSS	0.85	0.958	[0.931,0.974]	0.644	[0.603,0.683]
GPT-OSS	0.9	0.972	[0.946,0.986]	0.519	[0.477,0.560]
GPT-OSS	0.95	0.989	[0.961,0.997]	0.332	[0.294,0.372]
GPT-OSS	1			0	[0.000,0.007]

Table S37 | SemanticCertaintyDx supplementary comparison with ConsistencyDx on MIRA-v2.

Comparison of the semantic-entropy-inspired metric SemanticCertaintyDx with ConsistencyDx under baseline conditions on MIRA-v2. SemanticCertaintyDx was derived by clustering repeated diagnostic outputs into semantic groups, computing entropy over the resulting cluster distribution, and transforming entropy as exp(-entropy) so that larger values indicate higher confidence. The table reports discrimination performance (AUC), the DeLong comparison between SemanticCertaintyDx and ConsistencyDx, and their Pearson correlation. This analysis is provided for transparency and was not incorporated into the primary framework because of the high correlation between the two measures.

Metric	AUC	95% CI	Diff	DeLong P	Pearson Correlation
ConsistencyDx	0.860	[0.804,0.908]	0.029	0.024	0.865
SemanticCertaintyDx	0.832	[0.771,0.884]			

Table S38 | Age-stratified routing outcomes at ConsistencyDx ≥ 0.9 on MIRA-v2.

Exploratory post-hoc analysis of selective-autonomy routing at the main ConsistencyDx operating threshold used in the primary MIRA-v2 analysis. For each age group, the table reports retained cases, coverage, deferred cases, review fraction, residual autonomous-stream errors, deferred-stream errors, and retained accuracy. This analysis is descriptive and was not used for threshold optimization or causal interpretation of subgroup differences.

Age Group	Retained n	Coverage	Deferred n	Review fraction	Autonomous errors	Deferred errors	Retained accuracy

18-39	131	0.655	69	0.345	1	10	0.992
40-64	110	0.476	121	0.524	2	18	0.982
≥65	31	0.258	89	0.742	0	21	1.000
