# Supplementary material for: On-premise medical AI agents for reliable clinical decision-making
Source: Nat Med. 2026 Sep 15;32(10):3705–16. doi: 10.1038/s41591-026-04609-x (PMC13668652; doi:10.1038/s41591-026-04609-x)
Supplement: Supplementary file 2 [file 41591_2026_4609_MOESM2_ESM.pdf]

nature portfolio

Corresponding author(s): Jakob Nikolas Kather

Last updated by author(s): Jul 21, 2026

Reporting Summary

Nature Portfolio wishes to improve the reproducibility of the work that we publish. This form provides structure for consistency and transparency in reporting. For further information on Nature Portfolio policies, see our Editorial Policies and the Editorial Policy Checklist.

Statistics

For all statistical analyses, confirm that the following items are present in the figure legend, table legend, main text, or Methods section.

n/a	Confirmed	
☐	☒	The exact sample size (*n*) for each experimental group/condition, given as a discrete number and unit of measurement
☐	☒	A statement on whether measurements were taken from distinct samples or whether the same sample was measured repeatedly
☐	☒	The statistical test(s) used AND whether they are one- or two-sided<br>*Only common tests should be described solely by name; describe more complex techniques in the Methods section.*
☒	☐	A description of all covariates tested
☐	☒	A description of any assumptions or corrections, such as tests of normality and adjustment for multiple comparisons
☐	☒	A full description of the statistical parameters including central tendency (e.g. means) or other basic estimates (e.g. regression coefficient) AND variation (e.g. standard deviation) or associated estimates of uncertainty (e.g. confidence intervals)
☐	☒	For null hypothesis testing, the test statistic (e.g. *F*, *t*, *r*) with confidence intervals, effect sizes, degrees of freedom and *P* value noted<br>*Give P values as exact values whenever suitable.*
☒	☐	For Bayesian analysis, information on the choice of priors and Markov chain Monte Carlo settings
☒	☐	For hierarchical and complex designs, identification of the appropriate level for tests and full reporting of outcomes
☐	☒	Estimates of effect sizes (e.g. Cohen's *d*, Pearson's *r*), indicating how they were calculated

Our web collection on statistics for biologists contains articles on many of the points above.

Software and code

Policy information about availability of computer code

Data collection	Data were obtained from three benchmarks spanning two independent sources. CDM benchmark cases were downloaded from the MIMIC-IV-Ext-CDM resource hosted on PhysioNet and converted into the required evaluation format using custom Python scripts. MIRA-v2 analyses used a derived dataset generated by the benchmark developers from MIMIC-IV v2.2 and provided to us directly by the benchmark authors under the applicable data use agreement; at the time of writing, the dataset generation pipeline is not publicly available. VivaBench cases were obtained from the publicly available Hugging Face dataset release and were similarly converted into the agent input format. Agent runs and output generation were performed using custom Python code (Python 3.13.0).
Data analysis	Data analysis and visualization were performed in Python (3.11.0) using pandas (2.2.3), NumPy (2.2.4), scipy (1.16.1), statsmodels (0.14.5), and matplotlib (3.10.1) / seaborn (0.13.2).<br>Full pinned dependency list: pyproject.toml and uv.lock.

For manuscripts utilizing custom algorithms or software that are central to the research but not yet described in published literature, software must be made available to editors and reviewers. We strongly encourage code deposition in a community repository (e.g. GitHub). See the Nature Portfolio guidelines for submitting code & software for further information.

Data

Policy information about availability of data

All manuscripts must include a data availability statement. This statement should provide the following information, where applicable:

- Accession codes, unique identifiers, or web links for publicly available datasets
- A description of any restrictions on data availability
- For clinical datasets or third party data, please ensure that the statement adheres to our policy

Three benchmarks were analyzed in this study, spanning two independent data sources. The CDM and MIRA-v2 benchmarks are derived from MIMIC-IV v2.2 and are subject to PhysioNet credentialed access requirements, including completion of required training and agreement to the PhysioNet data use agreement for credentialed health data. MIRA-v2 can be reconstructed from MIMIC-IV v2.2 using the code released by the benchmark developers at https://github.com/Dyke-F/MIRA. CDM can be reconstructed using the code released by the benchmark authors at https://github.com/paulhager/MIMIC-Clinical-Decision-Making-Dataset and is also available to credentialed users through its PhysioNet release at https://www.physionet.org/content/mimic-iv-ext-cdm/1.0/. VivaBench is publicly available under a CC-BY-4.0 license on Hugging Face at https://huggingface.co/datasets/chychiu/VivaBench.

Research involving human participants, their data, or biological material

Policy information about studies with human participants or human data. See also policy information about sex, gender (identity/presentation), and sexual orientation and race, ethnicity and racism.

Reporting on sex and gender	Sex was considered in the analysis for descriptive reporting and subgroup diagnostic accuracy analyses (Extended Data Fig. E1). Age group was also used in an exploratory age-stratified post-hoc routing analysis on MIRA-v2 (Supplementary Table S38). Sex labels were taken from the benchmark source metadata and harmonized across datasets by mapping to common Female/Male categories. Gender identity information was not available, and no analyses were performed by gender identity.
Reporting on race, ethnicity, or other socially relevant groupings	Race and ethnicity were not used as study variables and no analyses were stratified by race or ethnicity.
Population characteristics	The study analyzed three retrospective clinical benchmarks spanning two data sources (MIMIC-IV-derived EHR benchmarks and the PubMed-derived VivaBench). Age group and sex (harmonized across datasets) were summarized descriptively and used for subgroup diagnostic accuracy analyses (Extended Data Fig. E1). No additional participant characteristics were collected by the authors.
Recruitment	This study used retrospective de-identified EHR data; no participants were recruited by the authors.
Ethics oversight	This study used de-identified, retrospective clinical data derived from MIMIC-IV (v2.2). The collection of patient information and creation of the MIMIC-IV resource were reviewed and approved by the Institutional Review Boards of Beth Israel Deaconess Medical Center and the Massachusetts Institute of Technology, with a waiver of informed consent. VivaBench analyses used publicly available clinical case vignettes drawn from published case reports; no participant recruitment or additional data collection was performed by the authors.

Note that full information on the approval of the study protocol must also be provided in the manuscript.

Field-specific reporting

Please select the one below that is the best fit for your research. If you are not sure, read the appropriate sections before making your selection.

☒ Life sciences ☐ Behavioural & social sciences ☐ Ecological, evolutionary & environmental sciences

For a reference copy of the document with all sections, see nature.com/documents/nr-reporting-summary-flat.pdf

Life sciences study design

All studies must disclose on these points even when the disclosure is negative.

Sample size	Sample sizes were determined by predefined benchmark cohorts (CDM: 2,400 cases; MIRA-v2: 551 cases; VivaBench: 990 cases). We evaluated all eligible cases available in each benchmark. No prospective sample-size calculation was performed because this was a retrospective benchmarking study.
Data exclusions	Data exclusions followed the benchmark definitions and pre-specified preprocessing. For the MIRA-v2 benchmark, we excluded the pancreatic cancer subset (n = 23) from the original benchmark for the pre-specified reasons described in Methods (modality mismatch, decision-target mismatch, and limited sample size). No other exclusions were applied.
Replication	Reproducibility was assessed by repeating the full benchmark evaluation across five independent runs under identical settings and reporting run-to-run variability (mean ± s.d.). All planned benchmark replications were completed successfully. Occasional technical inference failures were handled using the prespecified retry procedure and were not treated as failed experimental replications. Analysis code is available, and benchmark data are accessible under PhysioNet credentialed access and applicable third-party distribution terms (see Data availability).

Randomization	No experimental groups were created and no model training was performed; all available benchmark cases were used for evaluation. Randomization was applied only for physician adjudication. In Round 1, a stratified random subset of 111 MIRA-v2 cases (~20%) was selected for blinded review. In Round 2, an additional 70 cases were added to enrich underrepresented disease strata; these cases were randomly partitioned into three fixed subsets (X, Y, Z) and assigned to physician groups for inter-rater agreement analyses as described in Methods and Supplementary Table S3.
Blinding	Physician adjudication was blinded. In Phase I (Rounds 1–2), physicians generated independent diagnoses using the same structured case information provided to the agent, blinded to the dataset reference label and the agent output. In Phase II (Rounds 1–2), physicians assessed the clinical validity of two diagnoses presented in randomized order and were blinded to which diagnosis originated from the agent versus the dataset reference label. Physicians were also blinded to the automated LLM-judge decision. Disagreements were adjudicated by additional board-certified physicians, and final case-level outcomes were determined by majority vote within the assigned physician set.

Reporting for specific materials, systems and methods

We require information from authors about some types of materials, experimental systems and methods used in many studies. Here, indicate whether each material, system or method listed is relevant to your study. If you are not sure if a list item applies to your research, read the appropriate section before selecting a response.

Materials & experimental systems

n/a	Involved in the study
☒	☐ Antibodies
☒	☐ Eukaryotic cell lines
☒	☐ Palaeontology and archaeology
☒	☐ Animals and other organisms
☒	☐ Clinical data
☒	☐ Dual use research of concern
☒	☐ Plants

Methods

n/a	Involved in the study
☒	☐ ChIP-seq
☒	☐ Flow cytometry
☒	☐ MRI-based neuroimaging

Plants

Seed stocks	Not applicable.
Novel plant genotypes	Not applicable.
Authentication	Not applicable.
