# Supplementary material for: On-premise medical AI agents for reliable clinical decision-making
Source: Nat Med. 2026 Sep 15;32(10):3705–16. doi: 10.1038/s41591-026-04609-x (PMC13668652; doi:10.1038/s41591-026-04609-x)
Supplement: Supplementary file 3 [file 41591_2026_4609_MOESM3_ESM.pdf]

nature portfolio

Peer Review File

On-Premise Medical AI Agents for Reliable Clinical Decision-Making

Corresponding Author: Professor Jakob Kather

This file contains all reviewer reports in order by version, followed by all author rebuttals in order by version.

Version 0:

Reviewer comments:

Reviewer #1

(Remarks to the Author)
Intro

I liked the categorization of trust into operational and decisional dimensions. I think it is interesting, relevant, and provides a useful framing for the field.
I also fully agree with the statement: "In agentic systems, small stochastic differences can propagate across multi-step reasoning and tool use, producing divergent intermediate states and unsafe conclusions." This is an important point. Yet I think the authors should go deeper here. The problem is not only variation and non-determinism in outputs—which is a fact—but also the growing evidence that these models can systematically amplify biases, that labels and sociodemographic characteristics act as pressure points that change clinical recommendations in ways that are medically unjustified.
I tjink that this is directly relevant to the trust framing: stochastic variation may seem like noise at the individual level, but at scale it can produce inequitable, biased care. For a paper about trust in autonomous agents, I think this evidence must be acknowledged. I would suggest citing Omar et al. (Nat Med, 2025; doi:10.1038/s41591-025-03626-6), which showed outputs that sociodemographic labels shifted clinical recommendations, and maybe other similar works.
Second, the confidence estimation literature coverage is somewhat selective. The authors cite the Geng et al. NAACL 2024 survey and Wang et al. (2022) on self-consistency, which is very relevant, but do not engage with the substantial recent progress in this space. Specifically, the self-consistency approach of Wang et al. is cited, but there is now a much richer body of work on semantic entropy (and Farquhar et al., Nature, 2024; doi:10.1038/s41586-024-07421-0), confidence-weighted self-consistency (CISC; Taubenfeld et al., ACL 2025 Findings; doi:10.18653/v1/2025.findings-acl.1030), The very recent ConfiDx system (Zhou et al., npj Digital Medicine, 2025; doi:10.1038/s41746-025-02071-6) directly addresses uncertainty-aware diagnosis and outperformed standalone experts by 10.7% in uncertainty recognition. These works are directly relevant to the proposed framework and should be discussed to clarify what the current paper adds beyond existing multi-signal confidence approaches.
Third, the framing of on-premise deployment as a novel contribution would benefit from engaging with the recent npj Digital Medicine perspective on implementing LLMs in healthcare while balancing control, collaboration, costs, and security (Dennstädt et al., 2025; doi:10.1038/s41746-025-01476-7), which directly addresses the governance trade-offs discussed here.. Citing these would sharpen the argument for why on-premise deployment still constitutes an advance rather than restating what the field already recognizes.
Fourth, for me, the regulatory discussion (HIPAA, GDPR, PIPEDA) in paragraph 2 is thorough but disproportionately long. I would shorten it to 3–4 sentences and redirect the space to the literature gaps above.

Methods and scale
Dataset. Both benchmarks derive from MIMIC-IV (Beth Israel Deaconess Medical Center), meaning the agent is evaluated exclusively on one institution's documentation patterns, coding conventions, and patient population. For a paper that claims to provide a "validated blueprint" for clinical AI, I think external validation on at least one independent dataset would substantially strengthen the evidence. If this is not feasible, the limitation should be stated much more prominently than its current brief mention in the Discussion.
I agree that the framework does provide value in evaluating more general diagnostic and agentic capabilities. But I feel the statement "Together, these benchmarks enable a comprehensive evaluation of diagnostic reasoning depth, generalizability, and procedural decision-making in realistic clinical environments" overreaches, given that only 7 conditions (all acute abdominal/thoracic) are tested. The title similarly says "Clinical AI Agents" without qualification. I suggest toning this down to

match the actual scope.

Model selection. The authors state GLM-4.5-Air was selected for “the most reliable tool-call behavior” in their local serving stack, citing the model’s own technical report. I think this needs more transparency: how many candidate models were screened, what were the alternatives (e.g., Llama-3, Qwen-2.5, Mixtral), and what were the specific failure modes? Without this, the selection looks post hoc. Also, the comparison with GPT-5.2 is informative but asymmetric—GPT-5.2 is a much larger model. The finding that the performance gap is small (88.3% vs. 90.2%) is encouraging, but it reflects this specific pairing, not a general property of on-premise deployment. I suggest stating this nuance explicitly.

Planning strategy. I agree that removing the explicit Plan tool to reduce prompt overhead is an acceptable step given context-budget constraints. But for practicality and transparency, I think the authors should report, at least in the Extended Data,, information about token usage per encounter, wall-clock time per case, and GPU resource requirements. This would help readers assess the real-world feasibility of both the baseline agent and especially the consistency analysis (which requires 5 independent runs per case).

Multi-perspective confidence framework. This is the methodological centerpiece of the paper and is conceptually well motivated. I have some specific, maybe minor concerns about each component:
(1) ProbScore is defined as the geometric mean of token-level probabilities. I think this conflates linguistic fluency with diagnostic certainty. A well-trained LLM will assign high token probabilities to syntactically fluent text regardless of factual grounding. The stress test results (ProbScore remains high when accuracy degrades) confirm this.
(2) The LingCert hedging lexicon contains only 15 terms (Extended Data Table E1). This is quite limited compared to established uncertainty language resources. More importantly, the metric is purely surface-level: it counts hedging words as a fraction of total words without distinguishing whether hedging applies to the primary diagnosis or secondary findings. A trace saying “The patient likely has appendicitis, though we cannot rule out a UTI” would be scored the same as one expressing uncertainty about the primary diagnosis itself. I think the metric lacks the granularity needed for clinical interpretability.
(3) ConceptDensity_R showed an inverse association with correctness (AUC = 0.440) whic worse than chance. I think including a metric that anti-correlates with correctness as a “confidence signal” is misleading. I would suggest either reframing it explicitly as a “red flag” indicator (high density = elevated risk) or removing it from the primary framework and presenting it as a separate negative finding.
(4) Consistency is the strongest metric, and the cross-run semantic similarity approach is sound. However, the number of runs (N) is not clearly stated in the Methods. I assume N = 5 from the Extended Data, but this should be explicit. Have the authors tested sensitivity to N (e.g., N = 3 vs. N = 10)?

Regarding the statistical analysis, some points of thought:
AUC values are reported throughout without confidence intervals. For n = 551, bootstrapped 95% CIs should be reported. Without them, the reader cannot tell whether the difference between Consistency_Dx (AUC = 0.852) and ProbScore_Dx (AUC = 0.745) is statistically significant. I suggest a formal comparison (Im not an expeert in this specific area, but maybe DeLong test?).
Additionally, AUC values are reported throughout without confidence intervals. For n = 551, bootstrapped 95% CIs should be reported. Without them, the reader cannot tell whether the difference between Consistency_Dx (AUC = 0.852) and ProbScore_Dx (AUC = 0.745) is statistically significant. I suggest a formal comparison (e.g., DeLong test).

Results
I think the authors should avoid interpretive statements in the Results section. For example, “exceeding prior open-weight baselines including Gemma-3 (70.5%)” introduces a comparison framing that belongs in the Discussion. I suggest keeping the Results clean and factual, reporting numbers without editorializing.
I feel the section “Deconstructing Decisional Trust with a Multi-Perspective Confidence Framework” is somewhat dense and hard to read. There is a lot of information packed into a few paragraphs- eight confidence metrics across two streams, AUC values, distributional patterns, condition-level variation- all reported sequentially without a clear hierarchy. I suggest restructuring: lead with the main finding (Consistency_Dx is the strongest signal), then briefly describe the others, moving detailed per-condition results to the Extended Data.
The case studies in Figure 4b are well done and clinically informative. think this is one of the strongest parts of the paper.

minor point: The physician adjudication (Fig. 2e) has very small sample sizes per disease (pneumonia: n = 6). For the condition with the worst model performance (55.2%), 6 cases is not enough to draw reliable conclusions about clinical validity. I think this should be acknowledged.

Discussion
The finding that ConceptDensity inversely correlates with correctness is actually clinically important: it suggests that terminologically dense reasoning may signal confabulation rather than competence. I think this deserves more attention and, ideally, a post-hoc analysis comparing concept density in correct vs. incorrect traces against the number of tool calls or evidence retrieved.
I think the Discussion should address the computational cost of the consistency metric. Running 5 independent multi-turn agent encounters per case is expensive and slow. This is a practical barrier to deployment, especially in the on-premise setting where compute is more constrained than cloud.
The Discussion also does not address the decoding parameters (temperature, top-p) used for the stochastic runs. This directly affects consistency: low temperature inflates it, high temperature deflates it. The choice of sampling parameters is a confounder for the core metric of the paper and must be reported transparently and discussed as a limitation.

The opening claim that on-premise deployment "need not incur a large performance penalty" is based on one model pairing on one benchmark. I suggest tempering this to reflect that it is a specific finding, not a general property.

Some minor other comments:
- The abstract states the agent achieved accuracy "matching GPT-5.2." On AIDOC-v2, the gap is 88.3% vs. 90.2%—a 1.9-point difference. "Matching" implies equivalence. I suggest "approaching" or "comparable to."
- I honestly feel that he phrase "validated blueprint for the next generation of clinical AI" is overstated for a retrospective simulation study on a single data source with 7 conditions. The blueprint is promising but not yet validated in the sense required for clinical deployment.
- References 3 and 29 appear to cite the same document (NIST AI 600-1). References 17 and 30 both cite Vasey et al. DECIDE-AI with different journal attributions. These duplications should be resolved.

Overall
I do appreciate the authors work. It was a nice, refreshing (albeit somewhat technical) read. Please see my comments as suggestions and thoughts shared with you.
I think the core idea (like combining on-premise deployment with multi-signal confidence estimatio) is valuable and addresses a real need. The behavioral consistency metric is the most interesting contribution, and the dual-stream evaluation is a useful analytical lens. The case studies in Figure 4 are well executed.
However, several issues limit my enthusiasm: (1) the evaluation is confined to a single institution with a narrow disease scope, yet the claims are broad; (2) the confidence metrics beyond consistency show concerning properties (ProbScore miscalibrates under stress, LingCert has a minimal lexicon, ConceptDensity anti-correlates with correctness) that are acknowledged but not sufficiently addressed; (3) statistical reporting lacks confidence intervals for key results; (4) the literature engagement misses directly relevant recent work on LLM uncertainty, bias amplification, and confidence-aware agents; and (5) some claims overreach the evidence. I think the manuscript requires a good revision before it is suitable for Nature Medicine.

(Remarks on code availability)
The code is shared via Zenodo under CC-BY-4.0, which is appreciated

Reviewer #2

(Remarks to the Author)
The authors present an autonomous clinical AI agent framework evaluated on two diagnostic benchmarks derived from MIMIC. In addition to reporting diagnostic accuracy, the study proposes several confidence signals—behavioral consistency, linguistic certainty, and concept density—to estimate prediction reliability. Behavioral consistency across repeated stochastic runs is found to be the strongest indicator of correctness and is used to implement a selective autonomy triage workflow. The authors also perform physician adjudication on a subset of cases and conduct perturbation experiments simulating incomplete clinical information.

Originality and significance: The manuscript addresses an important problem in clinical AI deployment: determining when automated systems can safely operate without human oversight. The proposed selective autonomy framework is conceptually interesting, and the use of behavioral consistency as a confidence signal is a potentially useful methodological contribution. However, similar concepts exist in machine learning reliability and self-consistency reasoning methods. The novelty therefore lies mainly in applying these ideas to clinical agent workflows rather than introducing entirely new methodological principles.

Data & methodology: The overall experimental design—benchmark evaluation combined with confidence analysis and perturbation experiments—is reasonable. However, several limitations affect the strength of the conclusions. First, both datasets derive from the MIMIC ecosystem, limiting external validity. Second, the main confidence signal relies on repeated stochastic inference, yet the manuscript does not quantify the computational cost or latency implications of this approach in real clinical settings. Additional methodological clarification is needed regarding inference parameters, prompt design, number of repeated runs used for consistency estimation, and sensitivity of results to embedding models used in similarity calculations.

Statistics and treatment of uncertainties: Performance metrics are reported clearly, but statistical rigor could be improved. Confidence intervals for key metrics (e.g., AUC and coverage estimates) should be provided, and the process for selecting confidence thresholds should be clarified to avoid potential overfitting.

Conclusions: robustness and reliability: The results suggest that behavioral consistency correlates with prediction correctness and may help identify reliable AI outputs. However, the manuscript's broader claims regarding safe autonomous clinical deployment appear stronger than the evidence presented. The study remains a retrospective benchmark analysis, and additional validation would be required to support claims about real-world clinical reliability.

How to strengthen the manuscript:

-External or temporal validation using data outside the MIMIC ecosystem
-Quantification of computational cost and latency for repeated inference used in behavioral consistency
-Expanded physician adjudication with reporting of inter-rater agreement
-Analysis of the clinical severity of residual errors in the autonomous stream

-Additional robustness tests reflecting realistic clinical data shifts

References and credit to previous work: The manuscript generally cites relevant literature. However, discussion of uncertainty estimation could reference related work on self-consistency reasoning in language models.

Suggested improvements:

-Several additions could strengthen the manuscript:
-External or temporal validation using data outside the MIMIC ecosystem
-Quantification of computational cost and latency for repeated inference used in behavioral consistency
-Expanded physician adjudication with reporting of inter-rater agreement
-Analysis of the clinical severity of residual errors in the autonomous stream
-Subgroup analyses to evaluate performance across patient demographics
-Additional robustness tests reflecting realistic clinical data shifts

References: The manuscript generally cites relevant literature. However, discussion of uncertainty estimation could reference related work on self-consistency reasoning in LLMs.

Context: This manuscript addresses an important and timely problem in clinical AI: how to deploy autonomous or semi-autonomous diagnostic agents in healthcare settings that require strong governance, reliability, and human oversight. The focus on on-premise deployment and confidence-based selective autonomy is clinically relevant, as institutions increasingly seek AI systems that are both high-performing and operationally trustworthy. The manuscript is generally well-positioned within the growing literature on clinical LLM agents, although the claims would benefit from clearer positioning as a retrospective benchmark study rather than as a validated real-world deployment framework.

(Remarks on code availability)

Reviewer #3

(Remarks to the Author)
The authors build an on-prem agent using open-source LLMs and an existing framework for diagnostic decision making. The Physician agent interacts with the patient agent to solicit information and eventually make a decision. The authors compare the discriminative performance of different 'confidence signals' across two benchamrks using MIMIC-IV. They then simulate clinician workflows that incorporate the agent when highly confident. Overall this was a significant effort, however the work falls short in many places and the data do not support the conclusions. This is not ready for publication.

1. [On-prem vs. cloud-based solutions] The authors suggest they have demonstrated that an on-prem agent architecture can address trust gaps. As presented, though, the evidence mainly shows that locally run open-source models/tools can achieve similar performance to cloud-based proprietary models on two tasks drawn from a single dataset (MIMIC-IV).
-Because there are already many benchmarks comparing open-source (that can be run locally) and proprietary models, it’s not clear to me that this adds substantial new insight to that broader conversation.
-In addition, the evaluated models are entirely text-based. Since many diagnoses (e.g., pneumonia) often rely heavily on imaging data, this limits the clinical utility and generalizability of the conclusions.

2. [Integrating confidence signals] The paper also argues that interpretable confidence signals help address trust gaps. I think this is a promising direction, but several aspects of the current evaluation make the claim feel insufficiently supported:

-The experiments rely on a single LLM/agent and datasets; how do the observations generalize across agents? across datasets?
-The experiments rely on a single test set. Under distribution shift, models can be confident but wrong; communicating confidence while being wrong could harm trust. In the proposed design, agents act autonomously on “confident” cases, meaning clinicians might never notice this failure mode. That seems risky; some clinical oversight in all cases (even if only via random sampling) may be important.
-There was little to no justification for the various confidence metrics. Where did these come from? Moreover, why do we expect them to measure different things? ProbScore and Consistency should be highly correlated based on their underlying formulation - the closer the probability is to 0.5 the more randomness/inconsistency I'll observe in the output
-ProbScore normalizes for output length, but it still might disproportionately favor short outputs. Have the authors examined the relationship between output length and probabilistic score? (ConceptDensity may also be length-sensitive.)
-Discriminative performance of confidence signals is quantified using ROC analysis on a single test set per benchmark, which raises concerns about generalization. In Figure 3, are results combined across both benchmarks, and were patterns consistent across benchmarks? From the extended data, it appears relative discriminative performance varies substantially across diagnoses (e.g., pulmonary embolism vs. cholecystitis
-A key finding is that behavioral stability is a stronger indicator of diagnostic correctness than internal likelihood estimates. It’s not clear whether this will generalize beyond this model and dataset.
-All experiments are conducted in simulation, so it’s hard to anticipate how deferring “easy” cases would affect real clinician workflow and decision-making. For example, only seeing difficult cases might increase fatigue and potentially errors.
-Since diagnosis is ultimately binary (disease vs. no disease), why not map the generated output to a set of binary labels so

that “fuzzy” text matching isn’t required?

3. [Organization] I found the paper difficult to follow in places.
-Some results appear in the Methods section (e.g., performance with and without a critic agent). I actually found that comparison informative and interesting, but it felt buried in the methods/supplement and received little attention in the Introduction/Results/Discussion. I’d be interested to see these experiments expanded, including analysis of why the critic agent helps in some cases but not others.
-There are also a number of tangential experiments (relative to the central “trust gap” goal), such as adversarial stress testing. While interesting on their own, I wasn’t fully convinced of their direct connection to the main research question or hypothesis as currently framed.
-some of the citations are incomplete; despite the architecture being adapted from the AIDOC framework, citations 28 points to a paper that doesn't seem to exist (at least a google search doesn't return anything); this is concerning given how much the paper relies on this framework

(Remarks on code availability)
N/A

Version 1:

Reviewer comments:

Reviewer #1

(Remarks to the Author)
Thank you for revising this paper. I think that the revision was clearly taken seriously - the additional analyses and expanded discussion are appreciated and genuinely improve the work. I have four remaining points. I think none of them require major new experiments, but maybe they are worth thinking about carefully before the paper is finalized:

1. The demographic subgroup analyses added in revision (Extended Data Fig. E1) are a welcome addition. But I think the finding sitting in those panels is not a minor one. Accuracy drops from 91.8% in the 18-39 age group to 79.7% in the >=65 group on MIRA-v2, with a similar gradient in CDM. That is a 12-point performance gap in the patient population potentially carrying higher burden in ER . The limitation statement at lines 531-535 correctly acknowledges the absence of counterfactual demographic testing, and I appreciate that. But the Discussion does not engage with what the age gradient itself means for deployment safety and fairness. For a paper that explicitly frames a selective autonomy workflow, I think it is worth asking out loud: who is most likely to be handled autonomously versus deferred, and does this gradient map onto a group already at higher clinical risk?
I suggest adding at least a paragraph in the Discussion that engages with the finding directly - not just scopes it as a limitation. The data are already there. This would be a Discussion revision only.

2. Semantic entropy analysis - transparency: In response to my comment on the confidence estimation literature, the authors ran a semantic entropy-inspired analysis (SemanticCertaintyDx: AUC = 0.832 vs ConsistencyDx AUC = 0.860, DeLong p = 0.024, r = 0.86). I respect the decision not to incorporate this into the main text given the high correlation. But the analysis currently exists only in the response letter? it does not appear anywhere in the revised paper, nor the supplementary, at least from what I see. A reader has no way to know it was done or what it found. The result itself is actually reassuring: the two measures converge, and Consistency is statistically superior. I would suggest adding a brief supplementary table or note - not as a primary result, but for transparency. It would also be useful to others working in this space.

3. This is, for me, the one remaining somewhat substantive point: the revision added three new models and demonstrated that competitive diagnostic performance extends across architectures. That is a real and meaningful addition! BUT the confidence framework itself - all ConsistencyDx analyses, all ProbScore and LingCert ROC curves, selective autonomy simulations, the stress test, and the physician adjudication analyses - remains evaluated exclusively on GLM-4.5-Air. Qwen-3.5, which achieves 90.04% accuracy (slightly above the confidence model's 88.42%), was available during revision, yet no confidence metric analyses are reported for it.
The paper's central claim is that consistency-based gating is a deployable reliability framework. For that claim to hold at the level of this journal, I think it needs to be shown - at least partially - that ConsistencyDx behaves comparably as a reliability signal on models other than the one it was developed on. I would not suggest this requires a full replication across all four models. But running ConsistencyDx and the AUC analysis on at least one additional model - Qwen-3.5 seems the natural choice given its accuracy and availability - would meaningfully strengthen the generalizability argument that currently rests on a single model for the paper's primary contribution. A supplementary table would be sufficient.

4. ProbScore presentation is somewhat inconsistent with its revised framing, asthe Methods caveat at lines 833-836 is the right framing, and I appreciate it. But ProbScore still appears prominently in Fig. 3, the correlation matrix (Fig. 4a), and the selective autonomy comparison (Fig. 6b), presented alongside ConsistencyDx as a parallel reliability candidate. The stress-test result - ProbScore remains high, and in pneumonia actually increases, when accuracy degrades - is reported correctly. But the full implication of that finding is not drawn out in the Results narrative.
A reader working through Fig. 3 and Fig. 6 could reasonably conclude that ProbScore is a viable alternative to Consistency for triage gating, which the data do not support. I think a brief sentence in the Results when the stress-test findings are presented - explicitly noting that the behavior of ProbScore under evidence removal (stable or increasing despite accuracy collapse) is inconsistent with what a calibrated confidence signal should do - would bring the presentation into alignment

with the Methods framing already established. This is a small addition but would make the message cleaner and more consistent.

Overall, as said above, I think that this revision is a genuine improvement, and the authors engaged seriously with every point. The paper is - in fact - a novel and timely contribution to the field. Work on operational trust and decision-time reliability is needed at this stage of clinical AI deployment, and I think this paper makes a real contribution to that conversation. The four points above are specific and addressable, and I hope that the paper will be in very strong shape once they are considered. I appreciate the authors' work on this - it was a careful and substantive revision, and I thank them for taking the comments seriously.

(Remarks on code availability)

Reviewer #2

(Remarks to the Author)
The authors have adequately addressed my concerns.

(Remarks on code availability)

Reviewer #3

(Remarks to the Author)
Thank you for your response. Most of my original issues with the paper was with the over claiming - the conclusions were not supported by the experiments/data. You have largely addressed this by appropriately changing the claims; however some of the broader framing in the introduction remains.

Specifically, the introduction talks about ‘when an agent’s output is likely reliable enough to act on and when it should be reviewed,’ suggesting that in some cases there will be no human review, despite the author response that states they no longer present confidence-based routing as eliminating the need for oversight. The last sentence in the introduction says "...by helping determine when an agent's output may be reasonable to act on and when it should be escalated to human review.'

Since the proposed approach is framed as a risk stratification mechanism (with an AUC=0.86 for ConsistencyDX) it'd be helpful to see this further contextualized. What I want to know is what fraction of the cases need to be reviewed to guarantee no 'failures' i.e., cases where the model was confident but wrong. One can also sweep the review threshold, and as each point measure how many cases would have been confidently wrong (and not undergone review). I believe the ROC and PRC curves capture this additional context and would be good to include in a revision.

(Remarks on code availability)

Version 2:

Reviewer comments:

Reviewer #1

(Remarks to the Author)
I really, and once again, appreciate the author's serious work based on the review comments.
I wish the best for all, and have no further comments, as I see the revisions answer all of my points.
Best.

(Remarks on code availability)

Open Access This Peer Review File is licensed under a Creative Commons Attribution 4.0 International License, which permits use, sharing, adaptation, distribution and reproduction in any medium or format, as long as you give appropriate credit to the original author(s) and the source, provide a link to the Creative Commons license, and indicate if changes were made.
In cases where reviewers are anonymous, credit should be given to 'Anonymous Referee' and the source.
The images or other third party material in this Peer Review File are included in the article's Creative Commons license, unless indicated otherwise in a credit line to the material. If material is not included in the article's Creative Commons license and your intended use is not permitted by statutory regulation or exceeds the permitted use, you will need to obtain permission directly from the copyright holder.
To view a copy of this license, visit https://creativecommons.org/licenses/by/4.0/

Response to Reviewers

Manuscript NMED-A149668: “On-Premise Medical AI Agents for Reliable Decision-Making”

We thank all the reviewers for their helpful comments, which have really helped us to improve the manuscript. We have carefully revised the paper to incorporate the suggestions.

General clarification: Before responding point by point, we note three manuscript-wide clarifications made during revision. First, we corrected the description of the LLM-based evaluator used in the benchmark adjudication pipeline: the original submission referred to **GPT-4o**, whereas the evaluator actually used in the revised benchmark-scoring pipeline was **gemini-3.1-flash-lite-preview**. This correction resulted in **minor updates to some adjudication-based summary values**, but did **not** change the main findings or conclusions of the study. Second, to maintain consistency with the latest version of the related benchmark/framework manuscript, the benchmark/framework previously referred to as **AIDOC** is now referred to as **MIRA** throughout the revised manuscript. Third, we revised the title from **“On-Premise Confidence-Aware Autonomous Clinical AI Agents”** to **“On-Premise Medical AI Agents for Reliable Decision-Making”** to better match the scope of the revised manuscript. All responses below refer to the corrected evaluator description, updated terminology, revised title, and revised numerical values where applicable.

Reviewer #1 - Clinical AI

Introduction

Point 1.1 *- Trust framing*

I liked the categorization of trust into operational and decisional dimensions. I think it is interesting, relevant, and provides a useful framing for the field.

Response:

Thank you very much for this encouraging comment. We are grateful that you found the distinction between **operational** and **decisional** trust useful and relevant. This framing is central to the manuscript, and we have strengthened the revised paper further in response to your subsequent suggestions, including clearer positioning of the study, expanded methodological transparency, and additional analyses.

Point 1.2 *- Bias amplification and sociodemographic effects*

Comment: I also fully agree with the statement: "In agentic systems, small stochastic differences can propagate across multi-step reasoning and tool use, producing divergent intermediate states and unsafe conclusions." This is an important point. Yet I think the authors should go deeper here. The problem is not only variation and non-determinism in outputs—which is a fact—but also the growing evidence that these models can systematically amplify biases, that labels and sociodemographic characteristics act as pressure points that change clinical recommendations in ways that are medically unjustified.

I think that this is directly relevant to the trust framing: stochastic variation may seem like noise at the individual level, but at scale it can produce inequitable, biased care. For a paper about trust in autonomous agents, I think this evidence must be acknowledged. I would suggest citing Omar et al. (Nat Med, 2025; doi:10.1038/s41591-025-03626-6), which showed outputs that sociodemographic labels shifted clinical recommendations, and maybe other similar works.

Response:

Thank you for highlighting that trust in agentic clinical systems extends beyond stochastic non-determinism to include systematic bias amplification and inequitable recommendations driven by sociodemographic labels. We agree that this evidence is directly relevant to decisional trust in autonomous agents at scale. In the revised manuscript, we expanded the Introduction to explicitly acknowledge this failure mode and cited **Omar et al. (Nat Med 2025)** as an example demonstrating that sociodemographic labels can shift clinical recommendations in medically unjustified ways.

"Beyond stochastic variation, recent evidence demonstrates that LLMs can systematically amplify biases at scale: sociodemographic labels and patient characteristics have been shown to shift clinical recommendations in medically unjustified ways.(Omar et al., Nat Med, 2025)) That is, output variation that appears random at the level of individual cases may aggregate into systematic disparities across patient subgroups when deployed at scale, a risk that has been documented in recent LLM evaluations." (Lines 79-85)

We have added subgroup analyses stratified by patient demographics. Specifically, the revised figure now reports the age and sex composition of each benchmark cohort (**Extended Data Fig. E1a,b**) and model performance across age and sex subgroups (**Extended Data Fig. E1c**). These analyses show that performance heterogeneity was more apparent across age groups than across sex groups.

We further clarify that our added subgroup analyses evaluate performance heterogeneity across age and sex strata in the evaluated cohorts, but they do not constitute a formal counterfactual bias audit. Future evaluations of autonomous medical agents should combine repeated-run robustness testing with counterfactual demographic perturbation experiments to determine whether protected or socially salient attributes influence recommendations independent of clinical need.

"Finally, all evaluations were retrospective simulations; although we added descriptive age- and sex-stratified subgroup analyses, we did not perform counterfactual demographic label perturbation experiments. Prospective studies and dedicated bias audits will be required to determine how these signals affect clinician reliance, review burden, safety outcomes and fairness across patient subgroups in real practice." (Lines 531-535)

***Point 1.3** - Gaps in the confidence estimation literature*

Comment: Second, the confidence estimation literature coverage is somewhat selective. The authors cite the Geng et al. NAACL 2024 survey and Wang et al. (2022) on self-consistency, which is very relevant, but do not engage with the substantial recent progress in this space. Specifically, the self-consistency approach of Wang et al. is cited, but there is now a much richer body of work on semantic entropy (and Farquhar et al., Nature, 2024; doi:10.1038/s41586-024-07421-0), confidence-weighted self-consistency (CISC; Taubenfeld et al., ACL 2025 Findings; doi:10.18653/v1/2025.findings-acl.1030), The very recent ConfiDx system (Zhou et al., npj Digital Medicine, 2025; doi:10.1038/s41746-025-02071-6) directly addresses uncertainty-aware diagnosis and outperformed standalone experts by 10.7% in uncertainty recognition. These works are directly relevant to the proposed framework and should be discussed to clarify what the current paper adds beyond existing multi-signal confidence approaches.

Response:

Thank you for pointing us to this recent and highly relevant literature. We have revised the manuscript to discuss three related lines of work more explicitly: **semantic entropy** (Farquhar et

al., *Nature* 2024), **Confidence-Informed Self-Consistency (CISC)** (Taubenfeld et al., *ACL Findings* 2025), and **ConfiDx** (Zhou et al., *npj Digital Medicine* 2025).

For **semantic entropy**, we now cite Farquhar et al. to place our behavioral-consistency analysis in the broader context of meaning-level uncertainty across stochastic generations.

To assess its relevance to our task more directly, we also performed an additional semantic-entropy-inspired analysis on the MIRA-v2 baseline. We added a semantic-entropy-inspired confidence feature, **SemanticCertaintyDx**. The feature captures uncertainty at the level of diagnostic meaning by clustering repeated diagnostic outputs into semantic groups and computing entropy over the resulting cluster distribution. We then transform entropy as exp(-entropy) so that larger values indicate higher confidence.

SemanticCertaintyDx achieved strong discrimination performance (AUC = 0.832), confirming that semantic-level uncertainty is relevant to diagnostic reliability. However, ConsistencyDx remained significantly stronger (AUC = 0.860 vs. 0.832; DeLong p = 0.02364, FDR-corrected p = 0.02364), suggesting that our behavioral consistency metric captures reliability information not fully subsumed by semantic-cluster uncertainty. Because the two measures were also highly correlated (r = 0.86), we did not incorporate this additional metric into the main revised manuscript.

For **CISC**, we now discuss it as a related but methodologically distinct approach. CISC uses model-derived confidence to weight sampled reasoning paths during answer selection, whereas our study evaluates cross-run consistency as a **decision-time reliability signal** rather than an answer-selection mechanism.

For **ConfiDx**, we now cite it to clarify the distinction between uncertainty-aware diagnosis in **single-turn static clinical note interpretation** and our setting of **multi-step agentic clinical workflows** with iterative tool use and interactive information gathering.

We have also added a brief note in the **Discussion** on the concurrent work of **Kissling et al. (2026)**, whose confidence decomposition is conceptually aligned with our multi-perspective framing, while making clear that the task setting and operationalization differ from ours.

Changes made:

- Expanded the **Introduction** to discuss semantic entropy, CISC, and ConfiDx (Lines 93-98).
- Clarified how our framework differs from prior self-consistency and uncertainty-estimation approaches (Lines 98-102).
- Added a brief note in the **Discussion** on the conceptually related concurrent work by Kissling et al (Lines 450-454).

Point 1.4 *- On-premise deployment framing and missing governance literature*

Comment: Third, the framing of on-premise deployment as a novel contribution would benefit from engaging with the recent npj Digital Medicine perspective on implementing LLMs in healthcare while balancing control, collaboration, costs, and security (Dennstädt et al., 2025; doi:10.1038/s41746-025-01476-7), which directly addresses the governance trade-offs discussed here.. Citing these would sharpen the argument for why on-premise deployment still constitutes an advance rather than restating what the field already recognizes.

Response:

Thank you for the comments. We agree that the framing of on-premise deployment benefits from engagement with recent governance literature. In the revised Introduction, we now cite Dennstädt et al., npj Digital Medicine, 2025) in the **Introduction**.

"Beyond regulatory compliance, recent perspectives on healthcare LLM implementation have systematically articulated the trade-offs between control, collaboration, cost, and security in deployment choices (Dennstädt et al., npj Digital Medicine, 2025), reinforcing the practical relevance of institutionally governed deployment as a deliberate design decision rather than a default constraint." (Lines 57-61)

Point 1.5 *- Regulatory discussion is disproportionately long*

Comment: Fourth, for me, the regulatory discussion (HIPAA, GDPR, PIPEDA) in paragraph 2 is thorough but disproportionately long. I would shorten it to 3–4 sentences and redirect the space to the literature gaps above.

Response:

Thank you for the comments. We agree that the regulatory discussion was disproportionately detailed for an Introduction. In the revised manuscript, we condensed the regulatory paragraph

from its original length to three sentences that preserve the essential governance argument without enumerating the specific provisions of each regulatory framework (Lines 54-57). The space recovered has been redirected to the confidence estimation literature discussion described in our response to Point 1.2 above.

Methods and Scale

Point 1.6 *- Single-institution dataset and overreaching generalizability claims*

Comment: Dataset. Both benchmarks derive from MIMIC-IV (Beth Israel Deaconess Medical Center), meaning the agent is evaluated exclusively on one institution's documentation patterns, coding conventions, and patient population. For a paper that claims to provide a "validated blueprint" for clinical AI, I think external validation on at least one independent dataset would substantially strengthen the evidence. If this is not feasible, the limitation should be stated much more prominently than its current brief mention in the Discussion.

I agree that the framework does provide value in evaluating more general diagnostic and agentic capabilities. But I feel the statement "Together, these benchmarks enable a comprehensive evaluation of diagnostic reasoning depth, generalizability, and procedural decision-making in realistic clinical environments" overreaches, given that only 7 conditions (all acute abdominal/thoracic) are tested. The title similarly says "Clinical AI Agents" without qualification. I suggest toning this down to match the actual scope.

Response:

Thank you for the comments. We agree. The original manuscript overstated the scope of the evaluation given that the two primary benchmarks are both derived from MIMIC-IV and cover a limited set of diagnostic tasks. In the revised manuscript, we have moderated these claims throughout, including removal of language such as “validated blueprint” and revision of statements implying broad “generalizability” or “comprehensive evaluation” beyond the benchmark scope. We also revised the title accordingly, changing it from “**On-Premise Confidence-Aware Autonomous Clinical AI Agents**” to “**On-Premise Medical AI Agents for Reliable Decision-Making**” to better match the actual scope of the study and to avoid implying broader generalizability than is supported by the evaluated benchmarks.

To strengthen the evidence beyond the MIMIC-derived setting, we added an external benchmark evaluation on the independent PubMed-derived **VivaBench** dataset and now report both benchmark-level performance and selective-autonomy behavior in a new Results

subsection (**Extended Data Fig. E4; Supplementary Tables S10–S13**). We make clear, however, that this external benchmark broadens the evaluation setting but does not replace validation on independent clinical datasets or in prospective workflows.

We also revised the **Discussion** to state the single-institution origin and limited disease scope of the primary benchmarks more prominently, and we adjusted the framing of the study accordingly.

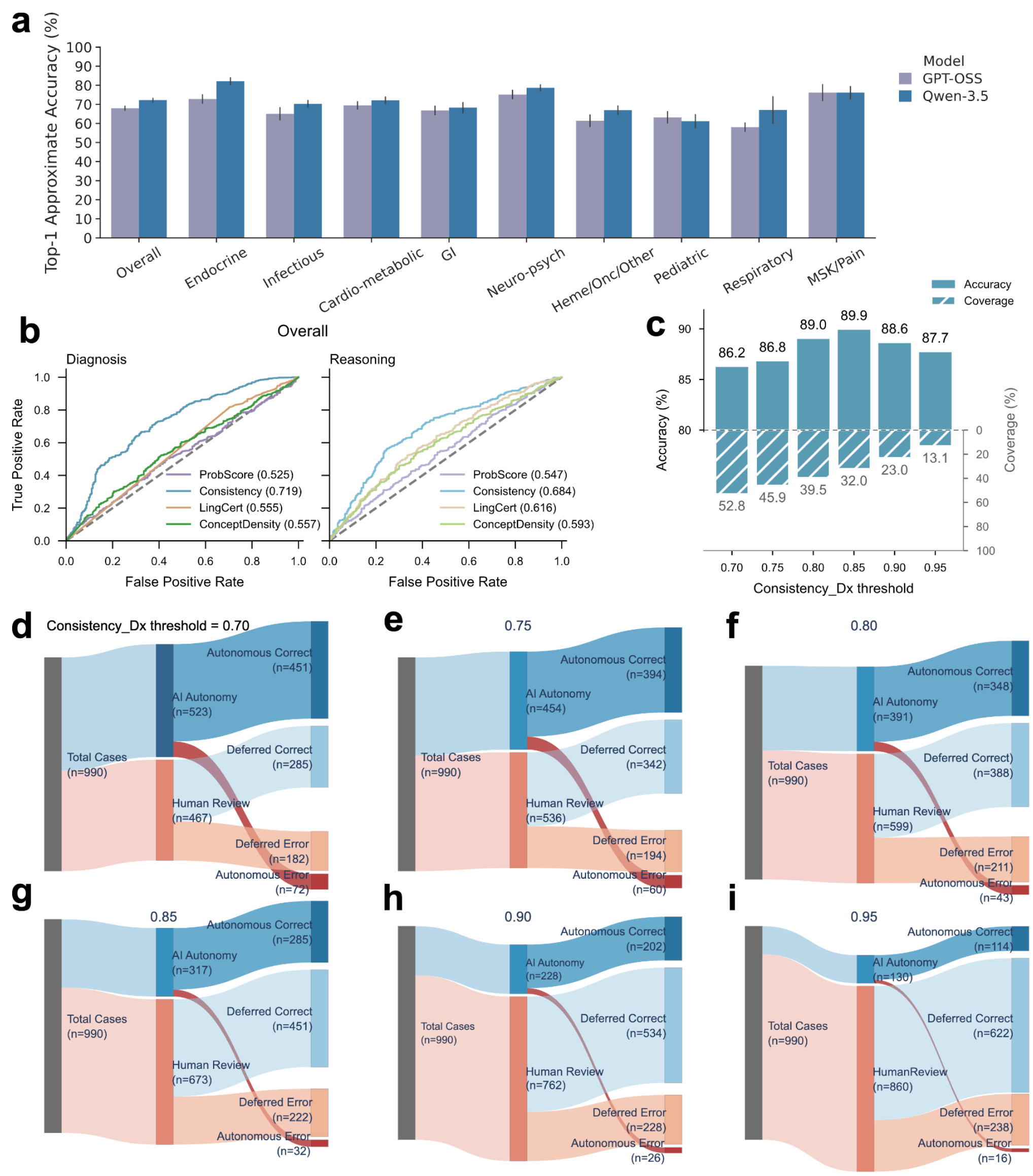

Fig. E4 | External benchmark evaluation and triage simulation on ViviaBench.

a, Because VivaBench is derived from physician-curated PubMed case reports and was designed to evaluate sequential reasoning under uncertainty, absolute performance is not directly comparable to the

MIMIC-derived benchmarks. Top-1 approximate accuracy on the ViviaBench benchmark, shown overall and across 10 specialty groups for the evaluated open-weight models. Overall accuracy was 67.96 ± 1.09 for GPT-OSS and 72.22 ± 0.92 for Qwen-3.5. **b,** ROC analysis of reliability-related metrics on ViviaBench. ConsistencyDx showed the highest discrimination of correctness among the evaluated metrics (AUC = 0.719), exceeding ConsistencyR (AUC = 0.684), whereas probability-based and linguistic/concept-density metrics showed weaker discrimination. **c,** Accuracy–coverage analysis for consistency-based case retention on ViviaBench. Accuracy was computed among retained cases, and coverage was defined as the proportion of all cases retained at each ConsistencyDx threshold. Within the evaluated threshold range, the highest retained-set accuracy was observed at a threshold of 0.85 (89.9% accuracy at 32.0% coverage). **d–i,** Selective-autonomy simulations on ViviaBench across increasing ConsistencyDx thresholds (0.70, 0.75, 0.80, 0.85, 0.90 and 0.95). Cases meeting the threshold were routed to AI autonomy and the remaining cases to human review. Flows show autonomous correct, deferred correct, deferred error, autonomous error, illustrating the trade-off between automation coverage and residual risk as the threshold becomes more stringent.

Changes made:

- Moderated scope-related claims throughout the manuscript, including removal/revision of "validated blueprint" and "comprehensive evaluation of generalizability" from **Methods**.

  "Together, these three benchmarks offer complementary evaluation perspectives, spanning diagnostic breadth (MIRA-v2), depth of reasoning (CDM), and cross-specialty generalizability (VivaBench)." (Lines 579-581)

- Revised the **title** to **"On-Premise Medical AI Agents for Reliable Decision-Making"** to better reflect the manuscript's scope.
- Added external benchmark evaluation on **VivaBench**.
- Expanded the **Discussion** to state the single-institution and limited-scope limitations more explicitly.

  "First, the primary benchmarks were derived from MIMIC-IV and therefore reflect a single-institution data ecology; although VivaBench broadens the evaluation setting, it does not replace validation on independent clinical datasets or in prospective workflows." (Lines 514-517)

Point 1.7 *- Model selection transparency*

Comment: Model selection. The authors state GLM-4.5-Air was selected for "the most reliable tool-call behavior" in their local serving stack, citing the model's own technical report. I think this needs more transparency: how many candidate models were screened, what were the alternatives (e.g., Llama-3, Qwen-2.5, Mixtral), and what were the specific failure modes? Without this, the selection looks post hoc. Also, the comparison with GPT-5.2 is informative but asymmetric—GPT-5.2 is a much larger model. The finding that the performance gap is small

(88.3% vs. 90.2%) is encouraging, but it reflects this specific pairing, not a general property of on-premise deployment. I suggest stating this nuance explicitly.

Response:

Thank you for this important point regarding model selection transparency. In our original submission, we did not adequately describe the screening process. We have now added a detailed account to the revised Methods (**Supplementary Table S24**). We screened four candidate open-weight models with documented tool-calling capabilities: Llama-3.3-70B-Instruct, Kimi K2.5, GLM-4.5V, and GLM-4.5-Air. Three models were excluded due to technical incompatibilities with our agentic framework or context-length constraints:

- **Llama-3.3-70B-Instruct** exhibited a systematic failure mode in which the physician agent bypassed the clinical dialogue phase entirely, proceeding directly to tool calls without eliciting patient history. Additionally, the model frequently generated malformed JSON for structured tool arguments (e.g., nested parameter lists for laboratory test requests), exceeding the three-retry error tolerance in a substantial proportion of encounters.

- **Kimi K2.5** could not be deployed due to unresolved tool-call parsing incompatibilities in our local serving infrastructure (vLLM), which at the time of our experiments lacked stable tool-parser support for these model architectures.

- **GLM-4.5V** was excluded due to context-length constraints that prevented completion of multi-turn clinical encounters with full tool-call histories.

GLM-4.5-Air was the only candidate that successfully completed the full encounter pipeline with reliable tool-call behavior, structured argument generation, and sufficient context capacity. We acknowledge that this selection was constrained by practical infrastructure compatibility rather than a comprehensive head-to-head accuracy comparison, and we have stated this explicitly in the revised manuscript.

To address the reviewers' broader concern about generalizability beyond a single model, the revised manuscript now reports results for three additional open-weight models that became available during revision: **GPT-OSS-120B, GLM-5 and Qwen3.5-397B-A17B** (Fig. 2, Table S1). **Gemma-4-31B** was screened but excluded because it frequently generated malformed JSON for structured tool arguments. Across the evaluated temperature settings, these additional models achieved comparable diagnostic accuracy (GPT-OSS: 85.3%; GLM-5: 89.65%;

Qwen3.5: 90.04%), demonstrating that competitive on-premise performance is not unique to GLM-4.5-Air.

Table S24 | Open-weight Model Screening

Model	Failure mode	Status
Llama-3.3-70B-Instruct	Skipped dialogue;<br>malformed JSON for structured arguments;	Excluded
Kimi K2.5	vLLM tool-parser incompatibility at time of experiments	Excluded
GLM-4.5V	Context-length limit exceeded in multi-turn encounters	Excluded
Gemma-4-31B	malformed JSON for structured arguments	Excluded
GLM-4.5-Air-FP8	–	Selected (primary)
GPT-OSS-120B*	–	Evaluated (revision)
GLM-5*	–	Evaluated (revision)
Qwen3.5-397B*	–	Evaluated (revision)

* Models added during manuscript revision to address reviewer concerns regarding generalizability across architectures.

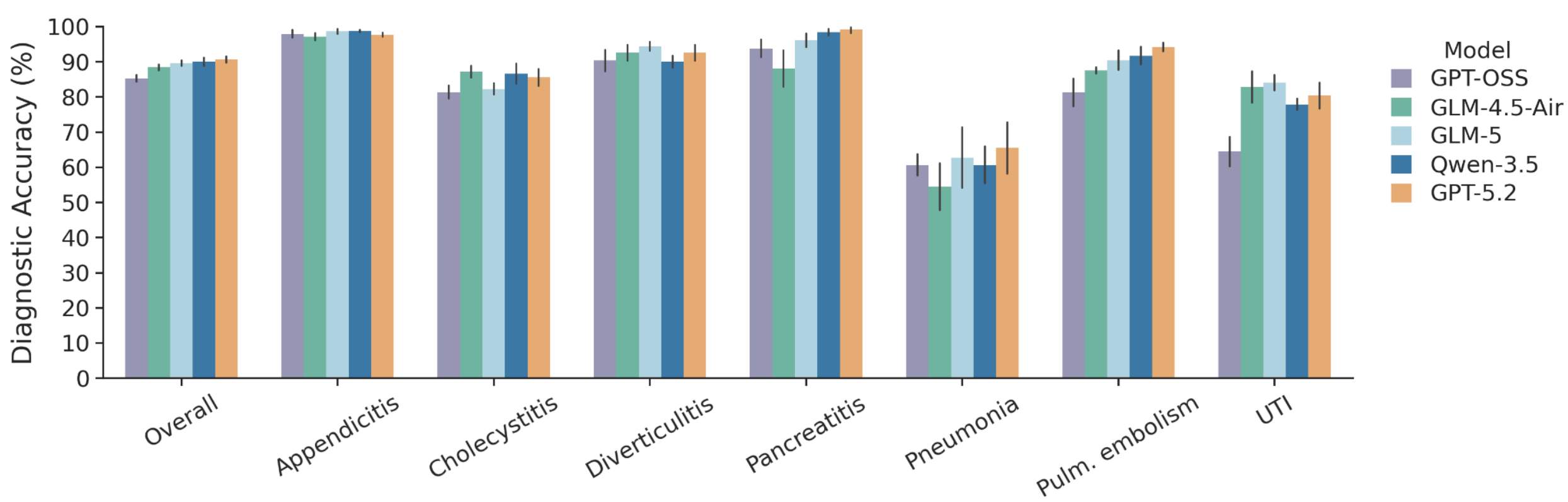

Fig. 2**a,** Diagnostic accuracy of four on-premise open-weight models (GLM-4.5-Air, GLM-5, Qwen-3.5, GPT-OSS) and a cloud baseline (GPT-5.2) across seven MIRA-v2 diseases. All systems used the same agent architecture and evaluation pipeline, differing only in the underlying LLM. For each model, accuracy is reported at the best-performing temperature setting; sensitivity to temperature is analyzed in the Implementation Sensitivity Analyses section.

Regarding the asymmetry of the GPT-5.2 comparison, we agree and have revised the manuscript to state this nuance explicitly. The cloud comparison is now presented as a matched benchmark reference for this specific model pairing and evaluation pipeline, rather than as evidence for a general property of on-premise deployment. We therefore describe the small gap between the best-performing on-premise model and GPT-5.2 as a setting-specific observation and avoid language implying broad equivalence between local and proprietary systems.

Changes made:

- Added a detailed description of model screening, exclusions, and failure modes (**Methods** (Lines 649-654)**; Supplementary Table S24**).
- Clarified that GLM-4.5-Air was selected for workflow compatibility and stable tool use, not through a comprehensive accuracy-only comparison (Lines 654-655).
- Added benchmark results for additional open-weight models (**Fig. 2; Supplementary Table S1**).
- Revised the GPT-5.2 comparison to make clear that it is a setting-specific matched reference rather than a general claim about on-premise deployment (Lines 432-435).

Point 1.8 *- Computational cost and infrastructure requirements*

Comment: Planning strategy. I agree that removing the explicit Plan tool to reduce prompt overhead is an acceptable step given context-budget constraints. But for practicality and transparency, I think the authors should report, at least in the Extended Data,, information about token usage per encounter, wall-clock time per case, and GPU resource requirements. This would help readers assess the real-world feasibility of both the baseline agent and especially the consistency analysis (which requires 5 independent runs per case).

Response:

Thank you for the comments. We agree and have added computational cost and infrastructure reporting in the revised manuscript. We now report full-benchmark token usage for single-run and five-run consistency inference (**Extended Data Fig. E5i**; **Supplementary Tables S20–S22**), controlled-subset wall-clock runtime (Supplementary Table S23), and deployment hardware requirements (**Extended Data Table E1**).

Across 44,077 single-run encounter-level observations, median doctor–agent token use was 125.0k tokens per encounter (IQR, 98.1–160.4k). Aggregating five runs at the case level

increased median doctor–agent token use to 636.4k tokens per case (IQR, 546.0–775.8k; n = 8,816), corresponding to an approximately 5.11-fold increase across model–temperature configurations. We also profiled wall-clock runtime in a controlled subset of 35 encounters per deployed model configuration, using the same disease-stratified subset for all local models. Median single-run encounter-level runtime ranged from 38.4 s to 125.6 s, and estimated serial five-run runtime ranged from 192.2 s to 627.8 s per case.

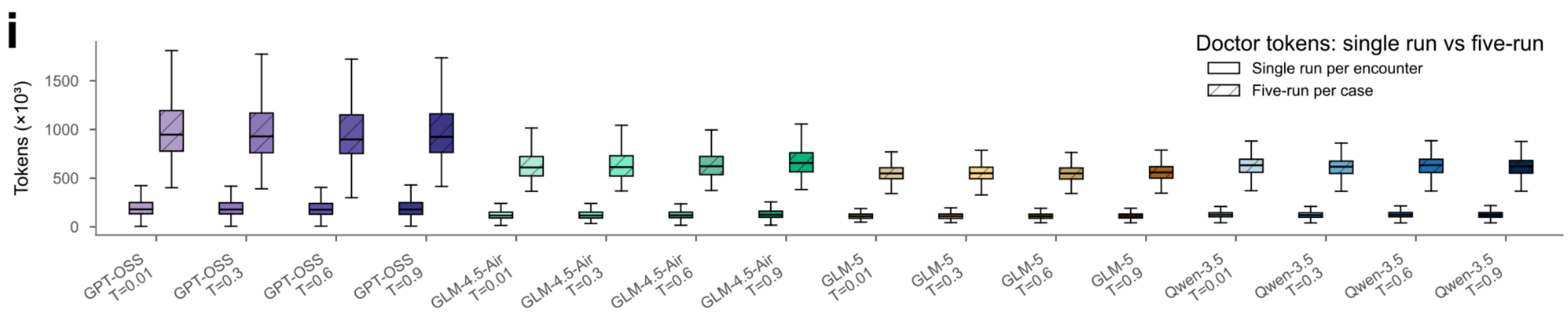

Fig. E5i, Box plots show doctor-agent token use per encounter for single-run inference and per case for five-run consistency inference across model families and decoding temperatures. Token counts are shown in thousands. For single-run inference, each point represents one encounter-level run; for five-run inference, each point represents the summed doctor–agent token used across five repeated runs for the same case. Colors denote model–temperature configurations, and hatched boxes indicate five-run inference. Across all configurations, median doctor–agent token use increased from 125.0k tokens for single-run inference to 636.4k tokens per case for five-run consistency inference, corresponding to an approximately 5.1-fold increase. GPT-OSS showed the highest token use, whereas GLM-5 showed the lowest.

Table S23 | Controlled subset wall-clock runtime by deployed model configuration.
Single-run encounter-level wall-clock runtime was measured in a controlled subset of 35 encounters per model, consisting of five randomly sampled cases from each of seven disease cohorts. The same subset was used for all deployed model configurations. Runtime was measured from simulation start to completion and includes the doctor agent, patient simulator, tool calls, and workflow overhead. Values are reported as mean, standard deviation (SD), median, interquartile range (IQR), minimum, and maximum. Estimated five-run consistency runtime was calculated as five times the measured single-run runtime under a serial execution assumption.

model	single_run_latency_mean_s	single_run_latency_sd_s	single_run_latency_median_s	single_run_latency_q1_s	single_run_latency_q3_s	single_run_latency_min_s	single_run_latency_max_s
GLM-4.5-Air	40.8	10.7	38.4	32.7	47.3	27.1	78.0
GLM-5	117.3	30.7	112.2	94.0	140.8	69.8	205.1
GPT-OSS	152.3	80.8	125.6	100.2	189.2	59.8	373.2
Qwen-3.5	117.2	60.7	96.0	70.3	149.5	55.3	295.3

We further revised **Extended Data Table E1** to report model identifiers, inference frameworks, generation settings, and GPU resources for the locally hosted vLLM deployments. In the revised manuscript, we clarify that **token use** is treated as the primary deployment-independent

measure of computational cost, whereas **wall-clock runtime** reflects the local deployment environment. We also discuss this trade-off explicitly in the revised Discussion.

Table E1 | Model sources, inference frameworks, and deployment settings.

**Model Name**	**Identifier**	**Source / access**	**Inference framework**	**Default T, top-p, top-k**	**GPU**
GLM-4.5-Air	zai-org/GLM-4.5-Air-FP8	Hugging Face weights; on-premise deployment	vLLM	1.0; 1.0; 0	2x H200
GLM-5	zai-org/GLM-5-FP8	Hugging Face weights; on-premise deployment	vLLM	1.0; 0.95;	8x H200
GPT-OSS	openai/gpt-oss-120b	Hugging Face weights; on-premise deployment	vLLM		1x H200
Qwen-3.5	Qwen/Qwen3.5-397B-A17B-FP8	Hugging Face weights; on-premise deployment	vLLM	0.6; 0.95; 20	4x H200
MedGemma	google/medgemma-27b-it	Hugging Face weights; on-premise deployment	vLLM		1x RTX PRO 6000 Blackwell
Gemini 3.1 Flash-Lite	google/gemini-3.1-flash-lite-preview	OpenRouter	OpenRouter API	1.0; 0.95; 64	-
GPT-5.2	gpt-5.2-2025-12-11	OpenAI	OpenAI API	-	-
all-MiniLM	sentence-transformers/all-MiniLM-L6-v2	Hugging Face	Sentence-Transformers	-	-
MPNet	sentence-transformers/all-mpnet-base-v2	Hugging Face	Sentence-Transformers	-	-
BGE	BAAI/bge-base-en-v1.5	Hugging Face	Sentence-Transformers	-	-
Gemma-3	google/gemma-3-27b-it	Not reported (leaderboard)	Not reported (leaderboard)	-	-

Note: H200 and RTX PRO 6000 Blackwell refer to NVIDIA GPUs. Hardware was reported only for locally hosted models; provider-hosted API models were listed as not reported.

Changes made:

- Added token-use profiling for single-run and five-run inference (**Extended Data Fig. E5i; Supplementary Tables S20–S22**).
- Added controlled-subset wall-clock runtime profiling (**Supplementary Table S23**).
- Added hardware and deployment details (**Extended Data Table E1**).
- Added corresponding discussion of the computational trade-off of consistency-based autonomy (Lines 505-513).

***Point 1.9** - ProbScore conflates linguistic fluency with diagnostic certainty*

Comment: (1) ProbScore is defined as the geometric mean of token-level probabilities. I think this conflates linguistic fluency with diagnostic certainty. A well-trained LLM will assign high token probabilities to syntactically fluent text regardless of factual grounding. The stress test results (ProbScore remains high when accuracy degrades) confirm this.

Response:

Thank you for the comments. We agree. In the revised manuscript, we no longer present ProbScore as a direct measure of diagnostic certainty, but rather as an internal likelihood proxy that may reflect local fluency or model preference without guaranteeing factual correctness. We now state this limitation explicitly in the **Methods** and **Discussion.** For example,

"Because this metric is derived from token-level generation likelihood within a single run, it may reflect local fluency or model preference without guaranteeing factual correctness or calibration. We therefore treated ProbScore as one component of a broader reliability framework rather than as a sufficient confidence measure on its own." (Lines 833-836)

***Point 1.10** - LingCert hedging lexicon is limited and lacks clinical granularity*

Comment: (2) The LingCert hedging lexicon contains only 15 terms (Extended Data Table E1). This is quite limited compared to established uncertainty language resources. More importantly, the metric is purely surface-level: it counts hedging words as a fraction of total words without distinguishing whether hedging applies to the primary diagnosis or secondary findings. A trace saying "The patient likely has appendicitis, though we cannot rule out a UTI" would be scored the same as one expressing uncertainty about the primary diagnosis itself. I think the metric lacks the granularity needed for clinical interpretability.

Response:

Thank you for this detailed and constructive critique of LingCert. We address each point in turn.

(1) Lexicon scope. We expanded the hedging lexicon from 15 to 35 terms, informed by the BioScope corpus annotation guidelines (Vincze et al., 2008) and the Hedging in Clinical Documents（Hanauer et al., 2012）. The refined lexicon covers modal verbs, hedging verbs, and probabilistic modifiers (full list in revised **Extended Data Table E3**). Initial experiments with a broadly inclusive lexicon revealed that terms with context-dependent meanings (e.g., frequency adverbs, certainty markers) introduced noise; we therefore retained only terms that unambiguously signal epistemic uncertainty. The curated lexicon improved LingCertDx AUC from 0.633 to 0.635, LingCertR AUC from 0.705 to 0.789 (full result in **Supplementary Table S33**).

Table E3 | Hedging Lexicon for the Linguistic Certainty Score (LingCert).

**Lexicon version**	**Count**	**Phrase / Word**
Original (compact)	15	possible, likely, suspected, cannot rule out, vs , might,

		probable, concern for, suggests, worrisome for, appears, indeterminate, question of, consider, suspicious for
Broad (inclusive, unfiltered)	48	Original (compact) + unlikely, certain, never, sometimes, rarely, often, almost, perhaps, occasionally, frequent, compatible with, improbable, always, probably, consistent with, possibly, not unreasonable, doubtful, not certain, low probability, usually, suggestive, not likely, low chance, no evidence of, possibility of, versus, uncertain, unclear, maybe, suspected, equivocal, not excluded
Refined (curated)	35	may, might, could, suggest, suggests, suggesting, indicate, indicates, appear, appears, seem, seems, suspect, suspected, possible, possibly, probable, probably, likely, unlikely, potential, potentially, presumed, uncertain, unclear, questionable, equivocal, improbable, doubtful, suggestive, approximate, perhaps, maybe, presumably, apparently

Table S33 | LingCertDx AUC across different versions of the hedging lexicon.

Lexicon version	LingCertDx AUC [95% CI]	LingCertR AUC [95% CI]
Original (compact)	0.633 [0.567, 0.702]	0.705 [0.629, 0.778]
Broad (inclusive, unfiltered)	0.632 [0.566, 0.701]	0.594 [0.515, 0.670]
Refined (curated)	0.635 [0.569, 0.705]	0.789 [0.727, 0.840]

(2) Granularity of hedging targets. The reviewer correctly notes that a sentence-level hedging count cannot distinguish whether uncertainty applies to the primary diagnosis or to secondary considerations. We acknowledge that within-sentence scope parsing remains beyond the current implementation. However, our dual-stream evaluation design provides a partial architectural solution to this concern. Because we compute LingCert separately on the diagnostic output (LingCertDx) and the reasoning trace (LingCertR), hedging in the short diagnostic output (e.g., "Likely acute cholecystitis") is more likely to target the primary diagnosis, whereas hedging in the longer reasoning trace often addresses differential considerations or secondary findings. Consistent with this interpretation, the two streams show substantially different discriminative performance: LingCertR achieved an AUC of 0.789, compared with 0.635 for LingCertDx, suggesting that hedging language carries different clinical significance depending on where it appears in the agent's output. We have added this observation to the revised Discussion.

(3) Surface-level limitations. We agree that lexicon-based hedging detection lacks the granularity needed for fine-grained clinical interpretability. Integrating scope-aware uncertainty parsing (e.g., leveraging dependency parsing or NLI-based methods to associate hedging cues with specific diagnostic entities, as explored in BioScope scope annotation) is an important direction for future work that could improve the clinical interpretability of expressed confidence signals. *(Lines 452-454)*

Point 1.11 *- ConceptDensity anti-correlates with correctness*

Comment: (3) ConceptDensityR showed an inverse association with correctness (AUC = 0.440) whic worse than chance. I think including a metric that anti-correlates with correctness as a "confidence signal" is misleading. I would suggest either reframing it explicitly as a "red flag" indicator (high density = elevated risk) or removing it from the primary framework and presenting it as a separate negative finding.

Response:

Thank you for the comments. We agree. In the revised manuscript, we no longer present ConceptDensityR as a positive confidence signal. Instead, we now describe it more cautiously as an **exploratory negative finding**: in the primary analysis, greater terminological density in the reasoning trace showed an inverse trend with correctness, but did not behave as a reliable confidence measure (AUC = 0.426; Holm-adjusted P = 0.08; **Fig. 3a,e**). We therefore revised the text, figure caption, and Discussion to make clear that ConceptDensityR should not be interpreted in the same way as ConsistencyDx or ProbScoreDx.

We also reduced its prominence in the main narrative. The revised Results now emphasize ConsistencyDx as the primary positive discriminator, while ConceptDensityR is described separately as a non-confirmatory inverse pattern rather than as a core confidence signal. In the Discussion, we interpret this result cautiously as raising the possibility that jargon-dense reasoning may project competence without corresponding factual grounding, while noting that this pattern should be regarded as exploratory.

Changes made:

- Reframed ConceptDensityR throughout the manuscript as an exploratory negative finding, not a positive confidence signal.

- Reduced its prominence in the primary framework narrative and separated it from the main positive discriminators in the Results.
- Revised the **Discussion** to interpret the finding cautiously as an **exploratory inverse trend**, rather than as a conventional confidence measure.

  “By contrast, in the primary MIRA-v2 analyses, concept density in reasoning did not behave as a confidence measure and instead showed an inverse trend with correctness, raising the possibility that jargon-rich rationales may project competence without corresponding factual grounding.”（*Lines 454-457*）

Point 1.12 *- Number of runs (N) for Consistency not stated; sensitivity to N not tested*

Comment: (4) Consistency is the strongest metric, and the cross-run semantic similarity approach is sound. However, the number of runs (N) is not clearly stated in the Methods. I assume N = 5 from the Extended Data, but this should be explicit. Have the authors tested sensitivity to N (e.g., N = 3 vs. N = 10)?

Response:

Thank you for the comments. We agree and have revised the manuscript accordingly. We now state explicitly in the **Methods / Experimental Configurations** that, unless otherwise noted, ConsistencyDx was estimated from **five** independent stochastic runs per case. We also added a dedicated implementation-sensitivity analysis comparing N = 3, N = 5, and N = 10.

Across these settings, the overall behavior of the consistency metric was stable. Increasing N produced only modest gains in retained-set accuracy but progressively reduced coverage, indicating a more conservative autonomy stream at larger N. For example, at ConsistencyDx ≥ 0.90, retained-set accuracy increased from 97.5% (N = 3) to 98.9% (N = 5) and 99.6% (N = 10), while coverage decreased from 51.7% to 49.4% and 46.3%, respectively. Paired analyses showed no detectable global shift in per-case consistency distributions across N (Friedman test, all evaluated thresholds: P = 0.123), although coverage differences became significant at more stringent thresholds (for example, Cochran’s Q: P = 0.002 at 0.90; P = 1.60 × 10^-8 at 0.95). We therefore retained N = 5 for the main analyses as a practical balance between retained-set accuracy, coverage, and computational cost.

These additions are now reported in the **Results** under “Implementation sensitivity analyses of behavioral consistency”, with full threshold-wise operating characteristics in **Supplementary**

Table S14, paired statistical comparisons in **Supplementary Table S15**, and corresponding visualizations in **Extended Data Fig. E5a–d**.

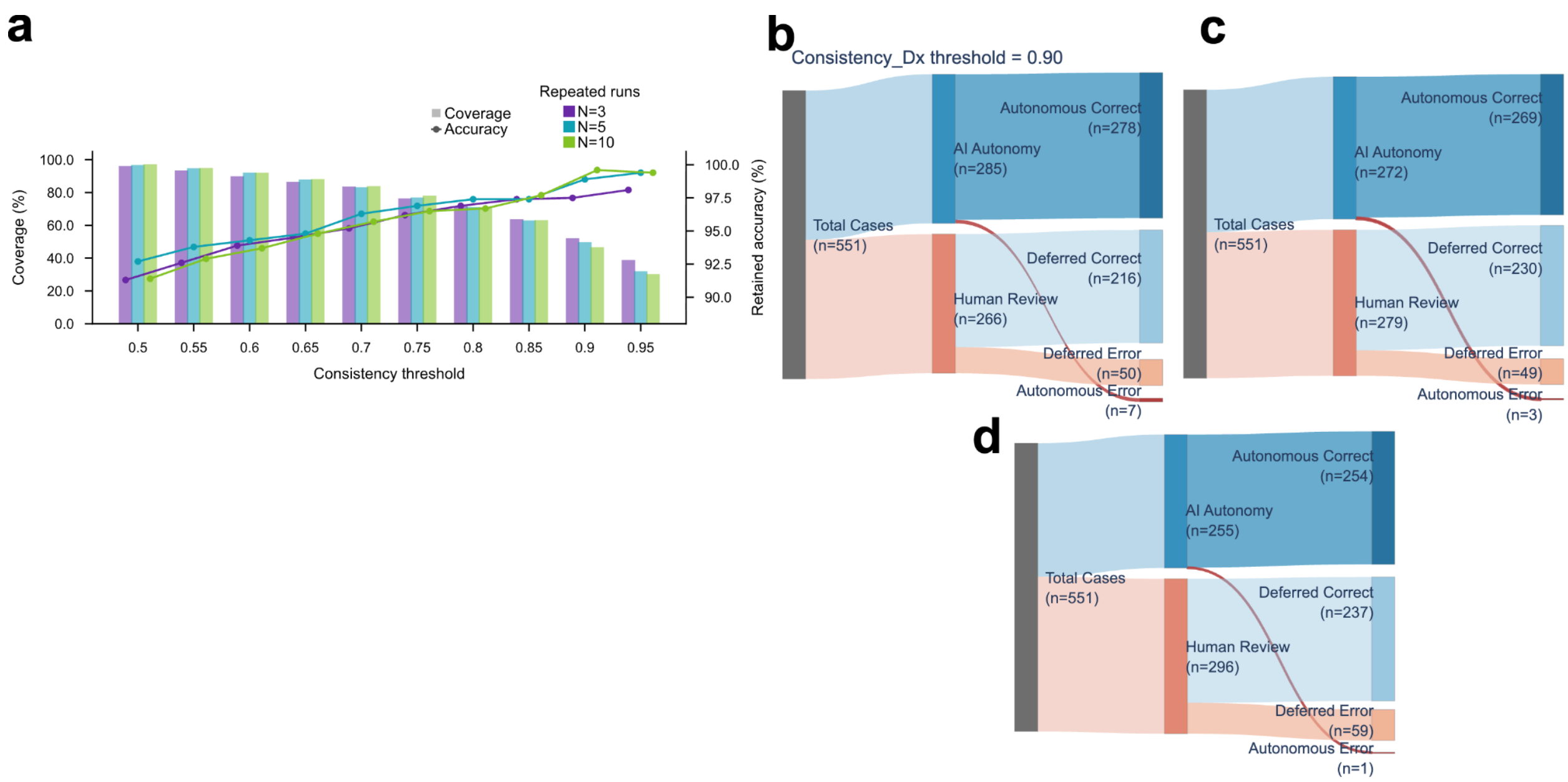

Fig. E5 | Implementation sensitivity analyses of behavioral consistency
a, Bars show coverage, defined as the percentage of cases retained at each ConsistencyDx threshold, and lines show retained accuracy, defined as diagnostic accuracy among retained cases. Results are shown for N=3, N=5, and N=10 repeated runs. Coverage and retained accuracy were broadly similar across sampling counts, with limited threshold-specific differences; coverage diverged mainly at higher thresholds, where N=10 retained fewer cases than N=3 and N=5. **b–d,** Representative clinical triage workflows at a ConsistencyDx threshold of 0.90 for N = 3, N = 5, and N = 10, respectively. As the number of runs increased, the autonomy stream became smaller and more selective, with fewer missed errors but greater review burden.

Point 1.13 *- AUC values without confidence intervals; formal comparison needed*

Comment: Regarding the statistical analysis, some points of thought: AUC values are reported throughout without confidence intervals. For n = 551, bootstrapped 95% CIs should be reported. Without them, the reader cannot tell whether the difference between ConsistencyDx (AUC = 0.852) and ProbScoreDx (AUC = 0.745) is statistically significant. I suggest a formal comparison (Im not an expeert in this specific area, but maybe DeLong test?).

Additionally, AUC values are reported throughout without confidence intervals. For n = 551, bootstrapped 95% CIs should be reported. Without them, the reader cannot tell whether the

difference between ConsistencyDx (AUC = 0.852) and ProbScoreDx (AUC = 0.745) is statistically significant. I suggest a formal comparison (e.g., DeLong test).

Response:

Thank you for the comments. We now report bootstrapped 95% confidence intervals for all AUC estimates and provide them in the supplementary tables associated with each analysis (Supplementary Tables S6, S8, S11 and S33). We also performed a formal comparison between the two primary diagnostic ROC curves using **DeLong's test**. ConsistencyDx showed significantly higher discriminative performance than ProbScoreDx (AUC = 0.860 versus 0.747; difference = 0.114, z = 3.256, P = 0.001131; FDR-adjusted P = 0.003394). This comparison is now reported in the main Results section. The Methods have also been revised to state explicitly that AUC confidence intervals were obtained by bootstrap resampling and that DeLong's test was used for formal comparison of correlated ROC curves where appropriate.

Changes made:

- Added bootstrapped 95% confidence intervals for all AUC estimates (**Supplementary Tables S6, S8, S11 and S33**).
- Added **DeLong** comparison for ConsistencyDx versus ProbScoreDx in the main Results section.
- Revised the Statistical Analysis subsection accordingly.

Results

Point 1.14 *- Interpretive statements in the Results section*

Comment: I think the authors should avoid interpretive statements in the Results section. For example, "exceeding prior open-weight baselines including Gemma-3 (70.5%)" introduces a comparison framing that belongs in the Discussion. I suggest keeping the Results clean and factual, reporting numbers without editorializing.

Response:

Thank you for the comments. We agree and have revised the Results to reduce interpretive framing. Specifically, we removed the comparative phrase "exceeding prior open-weight baselines including Gemma-3 (70.5%)," which was unnecessarily evaluative in tone. In the revised Results, we retain the factual benchmark values for the models evaluated in this study

and the previously reported open-weight reference value on CDM ("The highest previously reported open-weight result on this benchmark is 70.5% (Gemma-3)").

Changes made:

- Removed interpretive comparative wording from the Results section.
- Retained the prior published CDM reference value in factual form in Results (Lines 150-153).

***Point 1.15** - Confidence framework section is dense; clearer hierarchy needed*

Comment: I feel the section "Deconstructing Decisional Trust with a Multi-Perspective Confidence Framework" is somewhat dense and hard to read. There is a lot of information packed into a few paragraphs- eight confidence metrics across two streams, AUC values, distributional patterns, condition-level variation- all reported sequentially without a clear hierarchy. I suggest restructuring: lead with the main finding (ConsistencyDx is the strongest signal), then briefly describe the others, moving detailed per-condition results to the Extended Data.

The case studies in Figure 4b are well done and clinically informative. think this is one of the strongest parts of the paper.

Response:

Thank you for this helpful comment, and also for the positive assessment of the case studies in **Fig. 4b**. We are pleased that you found these analyses clinically informative, as we also view them as an important part of the manuscript.

We agree and have restructured this section for clarity. In the revised **Results**, the section now leads with the primary finding, namely that ConsistencyDx is the strongest discriminator of diagnostic correctness, followed by a shorter summary of the remaining metrics. We also separated the ROC-based ranking of metrics from the subsequent distributional analyses to reduce density and improve hierarchy. Detailed condition-level variation has been moved out of the main narrative and is now reported in **Extended Data Fig. E3** and the associated supplementary tables.

Changes made:

- Rewrote the section to lead with ConsistencyDx as the main result.

- Condensed the discussion of secondary metrics in the main text.
- Separated ROC/AUC comparisons from the description of score distributions.
- Moved detailed per-condition results to **Extended Data Fig. E3** and supplementary materials.

Point 1.16 *- Small sample sizes per disease in physician adjudication*

Comment: minor point: The physician adjudication (Fig. 2e) has very small sample sizes per disease (pneumonia: n = 6). For the condition with the worst model performance (55.2%), 6 cases is not enough to draw reliable conclusions about clinical validity. I think this should be acknowledged.

Response:

Thank you for this important point. We agree that the original physician-reviewed subset contained too few pneumonia cases (n = 6) to support reliable disease-level conclusions. In response, we conducted a second round of physician adjudication to expand coverage of underrepresented and clinically challenging conditions. Pneumonia was reviewed as a full census (n = 29); diverticulitis, pancreatitis, and UTI were each expanded by 15–16 cases to approximately 50% coverage. The total adjudicated sample increased from 111 to 181 cases (**33%** of the MIRA-v2 benchmark; **Supplementary Table S2**). Inter-rater agreement for both rounds is reported in Supplementary Table S4. The revised manuscript describes the two-round adjudication protocol in Methods and updates all corresponding analyses (**Fig. 2e,f; Supplementary Tables S2–S5**). The raw Agreement between the automated LLM-based judge and physician consensus changed **from 95.5% to 92.3%**.

Table S2 | Distribution of physician-reviewed cases across diagnostic categories.
Round 1 used stratified random sampling at approximately 20% coverage. Round 2 expanded coverage in low-prevalence categories: diverticulitis, pancreatitis, and UTI to ~50%, and pneumonia to 100% (full census).

Disease	Total N	Round 1 (n)	Round 2 (n)	Total reviewed (n)	% of total
Appendicitis	148	30	0	30	20
Cholecystitis	129	26	0	26	20
Pulmonary embolism	90	18	0	18	20
Diverticulitis	54	11	16	27	50

Pancreatitis	52	10	16	26	50
UTI	49	10	15	25	51
Pneumonia	29	6	23	29	100
Total	551	111	70	181	33

Discussion

Point 1.17 *- ConceptDensity finding deserves deeper analysis*

Comment: The finding that ConceptDensity inversely correlates with correctness is actually clinically important: it suggests that terminologically dense reasoning may signal confabulation rather than competence. I think this deserves more attention and, ideally, a post-hoc analysis comparing concept density in correct vs. incorrect traces against the number of tool calls or evidence retrieved.

Response:

Thank you for the comments. We agree that ConceptDensityR should not be interpreted as a conventional confidence signal. We have revised the Results to state explicitly that ConceptDensityR showed below-chance discrimination (AUC = 0.426) and no significant separation between correct and incorrect cases (Holm-adjusted P = 0.08; **Fig. 3a,e**), indicating that reasoning-trace concept density did not behave as a confidence signal.

In response to the suggestion, we also performed a post-hoc analysis comparing ConceptDensityR with diagnostic process variables, including the mean number of tool calls and the mean number of retrieved evidence items across runs. ConceptDensityR showed positive but limited associations with ToolCallCount_mean (Spearman $\rho = 0.131$, $P = 0.002$) and EvidenceRetrievedCount_mean ($\rho = 0.266$, $P = 2.14 \times 10^{-10}$; **Supplementary Fig. S1**). These analyses indicate that ConceptDensityR covaries with aspects of the diagnostic workup, particularly retrieved evidence count. In an adjusted logistic regression model including ConceptDensity_R, ToolCallCount_mean, EvidenceRetrievedCount_mean and dataset, ConceptDensityR was not independently associated with incorrectness (OR = 0.81 per 1 s.d., 95% CI 0.56-1.17, P = 0.263). We therefore do not present ConceptDensityR as a confidence score, but report it as an exploratory negative finding.

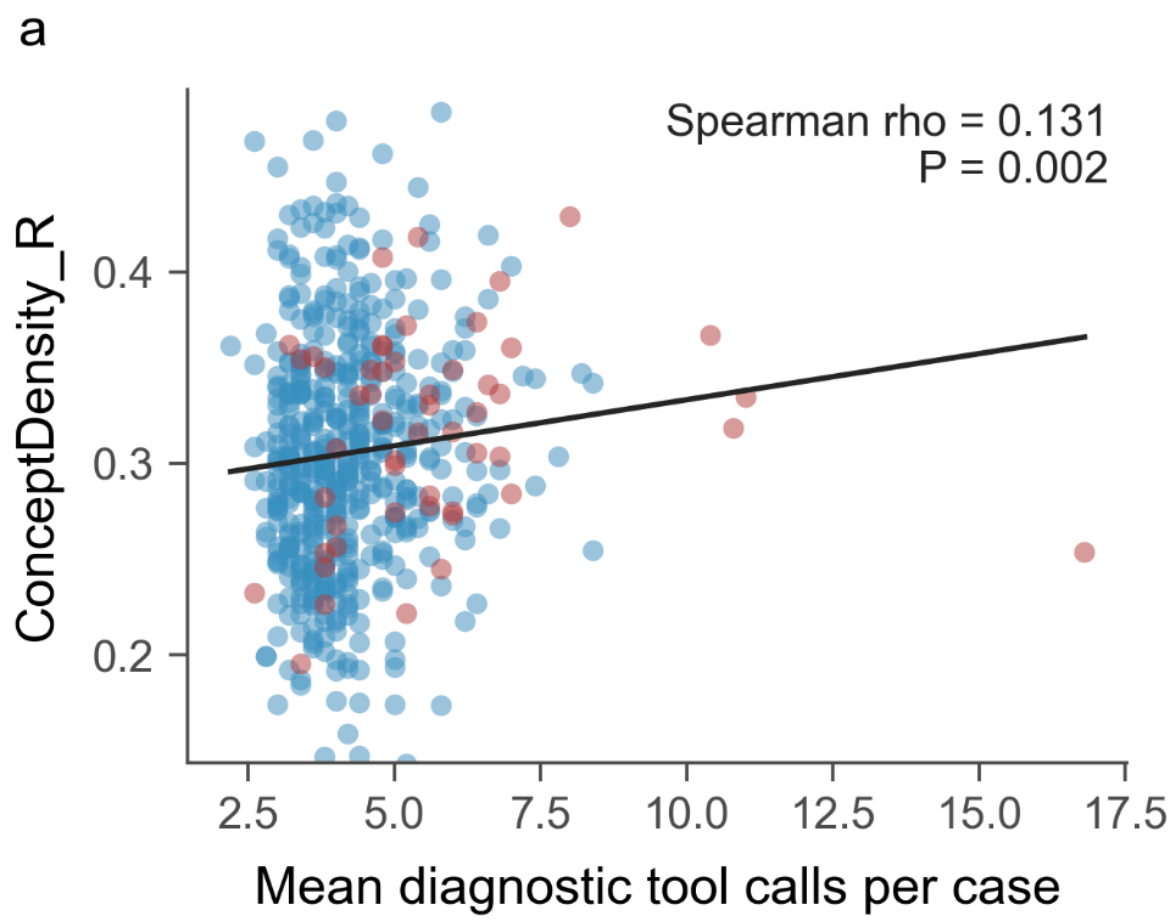

Supplementary Fig. S1 | Post-hoc analysis of reasoning-trace concept density against diagnostic process variables.
ConceptDensityR was compared with the mean number of diagnostic tool calls and retrieved evidence items across repeated runs. ConceptDensityR showed positive but limited associations with ToolCallCount_mean (Spearman $\rho = 0.131$, $P = 0.002$) and EvidenceRetrievedCount_mean ($\rho = 0.266$, $P = 2.14 \times 10$^-10). Correct-versus-incorrect separation is shown in Fig. 3e of the revised manuscript.

Point 1.18 *- Computational cost of the consistency metric*

Comment: I think the Discussion should address the computational cost of the consistency metric. Running 5 independent multi-turn agent encounters per case is expensive and slow. This is a practical barrier to deployment, especially in the on-premise setting where compute is more constrained than cloud.

Response:

Thank you for the comments. We agree and have added this point explicitly to the **Discussion**. In the revised manuscript, we now note that consistency-based autonomy introduces a practical deployment trade-off: estimating behavioral stability from five independent runs increased token use by approximately fivefold relative to single-pass inference and would also increase serial wall-clock latency. We further clarify that, although repeated runs are in principle parallelizable when sufficient serving capacity is available, total token consumption and GPU compute remain substantially higher. We therefore frame consistency as a useful but computationally costly reliability signal whose deployment value must be balanced against infrastructure constraints. (same with **Point 1.7**)

Changes made:

- Added explicit discussion of the computational cost of five-run consistency estimation in the **Discussion** (Lines 505-513).
- Linked this discussion to the new token-use and runtime profiling in **Extended Data Fig. E5i** and **Supplementary Table S20-S23**.

***Point 1.19** - Decoding parameters not reported; temperature is a confounder for Consistency*

Comment: The Discussion also does not address the decoding parameters (temperature, top-p) used for the stochastic runs. This directly affects consistency: low temperature inflates it, high temperature deflates it. The choice of sampling parameters is a confounder for the core metric of the paper and must be reported transparently and discussed as a limitation.

Response:

We now report the sampling parameters used for the stochastic runs explicitly in the **Methods / Experimental Configurations**, including the temperature setting for each model, and the model-specific default top-p and top-k settings (**Extended Data Table E1**). We also added a dedicated **implementation-sensitivity analysis** showing that decoding temperature shifts the absolute scale and operating point of consistency-based triage without overturning its qualitative discriminative behavior (**Extended Data Fig. E5e–h; Supplementary Tables S16**). In particular, higher temperatures reduced coverage at fixed ConsistencyDx thresholds while retained-set accuracy remained high. We now discuss this explicitly as a limitation in the **Discussion**, noting that absolute consistency thresholds are not universal and require calibration to the decoding configuration and deployment context.

Changes made:

- Added explicit reporting of temperature, top-p, and top-k settings in the **Methods** and Extended Data Table E2.
- Added temperature-sensitivity analyses in **Results** (Extended Data Fig. E5e–h; Supplementary Tables S16).
- Added a corresponding limitation in the **Discussion** on implementation-dependent threshold calibration.

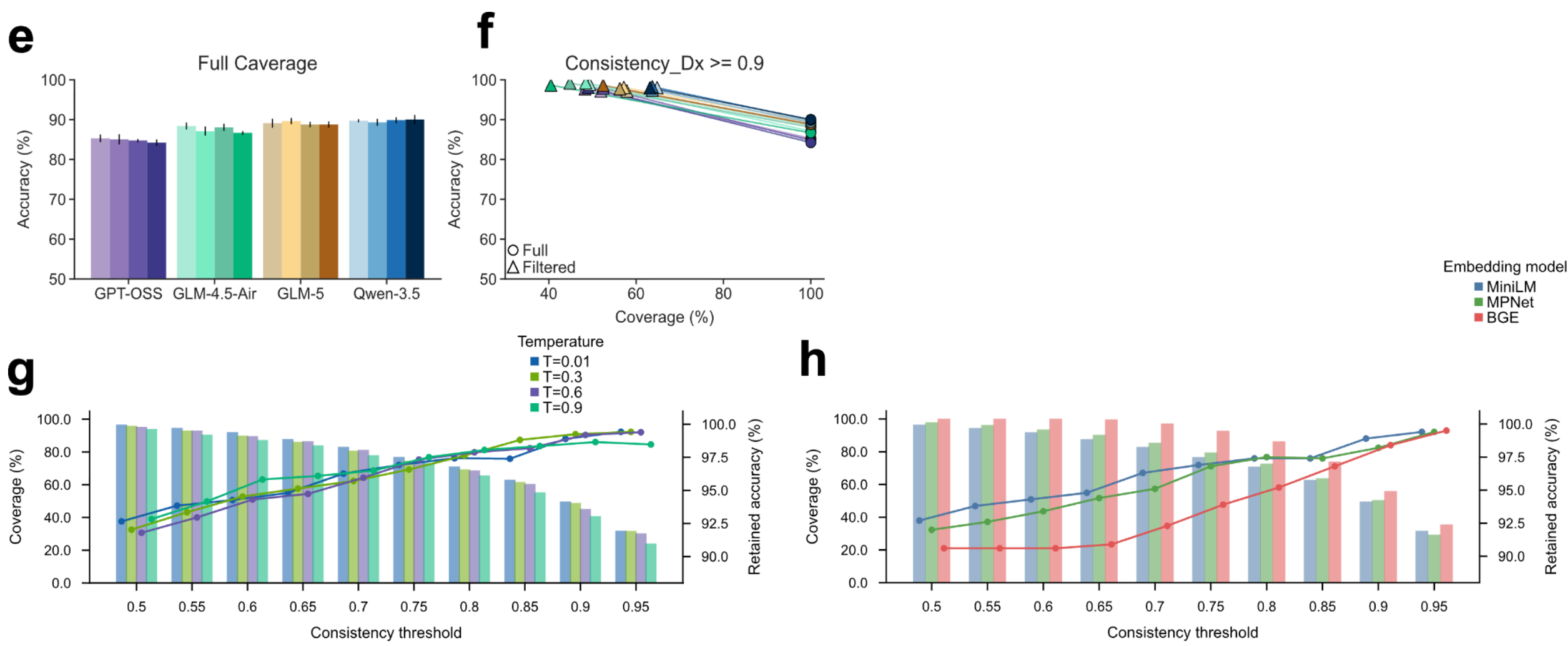

Fig. E5 | Implementation sensitivity analyses of behavioral consistency

e, Diagnostic accuracy at full coverage across decoding temperatures for the evaluated on-premise models. Within each model, accuracy varied only modestly across temperatures, indicating that decoding temperature did not materially change the overall ranking of model performance. Colors from light to dark correspond to temperatures of 0.01, 0.3, 0.6 and 0.9, respectively. **f,** Coverage–accuracy operating points for all evaluated models at full coverage (circles) and after consistency-based retention with ConsistencyDx ≥ 0.9 (triangles) across decoding temperatures. Increasing temperature produced a modest reduction in coverage at the retained-set operating point while retained-case accuracy remained high, with the largest shift observed for GLM-4.5-Air. **g,** Bars show coverage and lines show retained accuracy across ConsistencyDx thresholds for T=0.01, 0.3, 0.6, and 0.9 using GLM-4.5-Air. Coverage denotes the percentage of cases retained above each threshold, while retained accuracy denotes diagnostic accuracy among retained cases. Higher temperatures generally reduced coverage at fixed thresholds, particularly at thresholds ≥0.80, whereas retained accuracy remained high with comparatively small differences across temperatures. **h,** Bars show coverage and lines show retained accuracy across ConsistencyDx thresholds for MiniLM, MPNet, and BGE embeddings. Coverage denotes the percentage of cases retained above each threshold, while retained accuracy denotes diagnostic accuracy among retained cases. Embedding choice substantially changed coverage at fixed thresholds, with BGE retaining more cases than MiniLM and MPNet across most thresholds, indicating that absolute consistency thresholds are not directly interchangeable across embedding models.

Point 1.20 *- Claim about on-premise performance penalty should be tempered*

Comment: The opening claim that on-premise deployment "need not incur a large performance penalty" is based on one model pairing on one benchmark. I suggest tempering this to reflect that it is a specific finding, not a general property.

Response:

Thank you for this helpful comment. We agree and have tempered this claim in the revised manuscript. The **Discussion** now makes clear that the observed small on-premise/cloud gap is a setting-specific finding under a matched agent architecture and evaluation pipeline, rather than a general property of on-premise deployment. Specifically, we revised the text to state that on-premise performance on MIRA-v2 remained close to the cloud baseline for the top-performing models in this study, and we added the qualifier “in this setting” to avoid overgeneralization.

Changes made:

- Revised the corresponding statement in the **Discussion** to frame the on-premise/cloud comparison as a specific finding in the evaluated setting rather than a general claim.
- Added wording that limits the observation to the top-performing models and the matched architecture/evaluation pipeline used here.

  “Under a matched agent architecture and evaluation pipeline, on-premise performance on MIRA-v2 remained close to the cloud baseline for the top-performing models, indicating that governance-oriented local deployment did not impose a large performance penalty in this setting.” (Lines 432-435)

Point 1.21 *- Minor: 'matching GPT-5.2' overstates equivalence*

Comment: - The abstract states the agent achieved accuracy "matching GPT-5.2." On MIRA-v2, the gap is 88.3% vs. 90.2%—a 1.9-point difference. "Matching" implies equivalence. I suggest "approaching" or "comparable to."

Response:

Thank you for the comments. We agree and have revised the wording accordingly. In the original abstract, “matching GPT-5.2” overstated the relationship between the best on-premise result and the cloud baseline. We now use more precise language (“**approaching**”) to reflect

the observed difference on MIRA-v2. In the revised **Results**, the best-performing on-premise model (Qwen3.5, 90.04%) is described as performing within 0.7 percentage points of GPT-5.2 (90.7%), rather than as equivalent.

Changes made:

- Replaced “matching GPT-5.2” with more precise wording in the **Abstract.**

  *“Across two MIMIC-IV-derived benchmarks, the agent achieved 90.04% accuracy on a 7-disease task and 83.8% on a 4-disease task, **approaching** a cloud baseline on the primary benchmark.” (Lines 32-34)*

- Revised related phrasing in the **Results** and **Discussion** to avoid implying equivalence.

  “GPT-5.2 achieved 90.7%, placing the best on-premise model within 0.7 percentage points of the cloud baseline.” (Lines 134-136)

***Point 1.22** - Minor: 'validated blueprint for the next generation of clinical AI' is overstated*

Comment: - I honestly feel that he phrase "validated blueprint for the next generation of clinical AI" is overstated for a retrospective simulation study on a single data source with 7 conditions. The blueprint is promising but not yet validated in the sense required for clinical deployment.

Response:

Thank you for the comments. We agree and have removed this phrase from the revised manuscript. The original wording overstated the level of validation supported by a retrospective benchmark study with limited diagnostic scope. In the revision, we replaced it with more restrained language that presents the framework as a practical and promising approach for trustworthy clinical agents, rather than as a validated blueprint for clinical deployment.

Changes made:

- Removed the phrase “validated blueprint for the next generation of clinical AI.”
- Replaced it with more cautious wording in the Abstract and related summary statements.

  “These findings support a practical framework for institutionally governed clinical agents in which decision-time reliability signals identify a lower-risk subset for autonomous handling and defer the remainder for review.” (Lines 38-41)

Point 1.23 *- Minor: Duplicate references (Refs 3/29 and Refs 17/30)*

Comment: - References 3 and 29 appear to cite the same document (NIST AI 600-1). References 17 and 30 both cite Vasey et al. DECIDE-AI with different journal attributions. These duplications should be resolved.

Response:

Thank you for the comments. We agree and have corrected the reference list accordingly. The duplicate citation to NIST AI 600-1 has been removed, and the duplicated DECIDE-AI reference has been consolidated with the correct journal attribution. We also rechecked the full reference list for consistency after revision.

Changes made:

- Removed the duplicate citation to NIST AI 600-1.
- Consolidated the duplicated DECIDE-AI reference and corrected its journal attribution.
- Reviewed the revised reference list for accuracy and consistency, and replaced several in-text citations with more appropriate supporting references, including the introductory sentence on autonomous medical AI-agent capabilities:

  "Autonomous artificial intelligence (AI) agents powered by large language models (LLMs) can now conduct multi-turn clinical dialogues, gather diagnostic evidence and produce diagnoses with accompanying rationales.(Schmidgall et al., 2024; Moor, M. et al, npj Digital Medicine, 2024; Ke, Y. et al., npj Digital Medicine, 2025; Zhou et al., npj AI, 2025)." (Lines 43-45)

Reviewer #2 - Bioinformatics

Point 2.1 *- Overall assessment of the framework*

Comment: - The authors present an autonomous clinical AI agent framework evaluated on two diagnostic benchmarks derived from MIMIC. In addition to reporting diagnostic accuracy, the study proposes several confidence signals—behavioral consistency, linguistic certainty, and concept density—to estimate prediction reliability. Behavioral consistency across repeated stochastic runs is found to be the strongest indicator of correctness and is used to implement a selective autonomy triage workflow. The authors also perform physician adjudication on a

subset of cases and conduct perturbation experiments simulating incomplete clinical information.

Response:

Thank you very much for this accurate summary of the manuscript and its main components. We are pleased that you identified the central contributions of the study, including the confidence framework, physician adjudication, and perturbation-based evaluation. In the revised manuscript, we have further clarified the scope and interpretation of these analyses in response to your subsequent suggestions.

Point 2.2 *- Originality and significance; novelty mainly in application*

Comment: - Originality and significance: The manuscript addresses an important problem in clinical AI deployment: determining when automated systems can safely operate without human oversight. The proposed selective autonomy framework is conceptually interesting, and the use of behavioral consistency as a confidence signal is a potentially useful methodological contribution. However, similar concepts exist in machine learning reliability and self-consistency reasoning methods. The novelty therefore lies mainly in applying these ideas to clinical agent workflows rather than introducing entirely new methodological principles.

Response:

Thank you for this thoughtful comment and for recognizing the importance of the deployment problem addressed here, as well as the conceptual interest of the selective-autonomy framework. We agree with this characterization and have revised the manuscript to position the contribution more precisely. The revised text now makes clearer that the novelty lies primarily in the clinical operationalization and systematic evaluation of these ideas within an autonomous, on-premise diagnostic agent workflow, rather than in proposing an entirely new uncertainty-estimation or self-consistency principle. Specifically, we now emphasize the integration of dual-stream confidence analysis, stress testing under reduced evidentiary grounding, and selective-autonomy triage in a multi-step clinical agent setting.

Changes made:

- Revised the **Introduction** and **Discussion** to position the contribution more explicitly as application and operationalization in clinical agent workflows (Lines 116-120; 536-541).

- Expanded the **Introduction** and **Methods** to clarify how our framework relates to prior work on self-consistency and uncertainty estimation (Lines 93-102; 810-814).

  e.g "This design was informed by prior work on self-consistency reasoning, semantic uncertainty estimation, and uncertainty-aware diagnostic systems[26,28,41], but differs in that the present study evaluates these signals jointly within an autonomous clinical agent workflow and computes them separately for both the final diagnostic output (Dx) and the accompanying reasoning trace (R)." (Lines 810-814)

Point 2.3 *- Dataset limitations and missing methodological clarifications*

Comment: - Data & methodology: The overall experimental design—benchmark evaluation combined with confidence analysis and perturbation experiments—is reasonable. However, several limitations affect the strength of the conclusions. First, both datasets derive from the MIMIC ecosystem, limiting external validity. Second, the main confidence signal relies on repeated stochastic inference, yet the manuscript does not quantify the computational cost or latency implications of this approach in real clinical settings. Additional methodological clarification is needed regarding inference parameters, prompt design, number of repeated runs used for consistency estimation, and sensitivity of results to embedding models used in similarity calculations.

Response:

Thank you for the comments. We agree and have strengthened the manuscript accordingly. First, to extend the evaluation beyond the MIMIC-derived benchmarks, we added an external benchmark analysis on the independent PubMed-derived VivaBench dataset and now report both benchmark-level performance and selective-autonomy behavior in a new Results subsection (**Extended Data Fig. E4; Supplementary Tables S10–S13**). We make clear, however, that this broadens the evaluation setting but does not replace validation on independent clinical datasets or in prospective workflows.

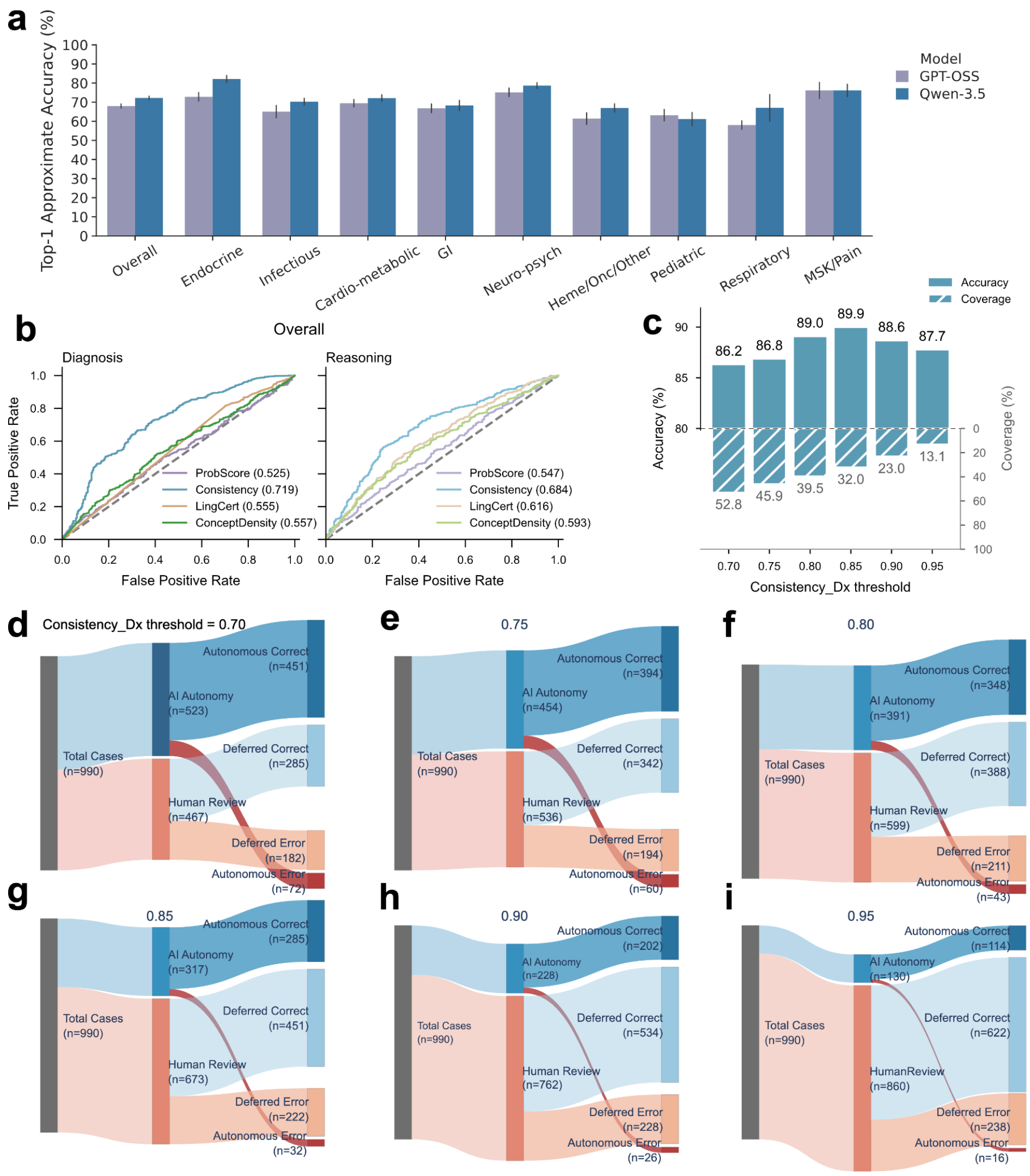

Fig. E4 | External benchmark evaluation and triage simulation on ViviaBench.

a, Because VivaBench is derived from physician-curated PubMed case reports and was designed to evaluate sequential reasoning under uncertainty, absolute performance is not directly comparable to the MIMIC-derived benchmarks. Top-1 approximate accuracy on the ViviaBench benchmark, shown overall and across 10 specialty groups for the evaluated open-weight models. Overall accuracy was 67.96 ± 1.09 for GPT-OSS and 72.22 ± 0.92 for Qwen-3.5. **b,** ROC analysis of reliability-related metrics on ViviaBench. ConsistencyDx showed the highest discrimination of correctness among the evaluated metrics (AUC = 0.719), exceeding ConsistencyR (AUC = 0.684), whereas probability-based and linguistic/concept-density metrics showed weaker discrimination. **c,** Accuracy–coverage analysis for consistency-based case retention on ViviaBench. Accuracy was computed among retained cases, and coverage was defined as the proportion of all cases retained at each ConsistencyDx threshold. Within the evaluated threshold range, the highest retained-set accuracy was observed at a threshold of 0.85 (89.9% accuracy at 32.0% coverage). **d–i,** Selective-autonomy simulations on ViviaBench across increasing ConsistencyDx thresholds (0.70, 0.75, 0.80, 0.85, 0.90 and 0.95). Cases meeting the threshold were routed to AI autonomy and the remaining cases to human review. Flows show autonomous correct, deferred correct,

deferred error, autonomous error, illustrating the trade-off between automation coverage and residual risk as the threshold becomes more stringent.

Second, we now quantify the computational implications of repeated-run consistency estimation. The revised manuscript reports token usage for single-run and five-run inference, controlled-subset wall-clock runtime, and deployment hardware requirements (**Extended Data Fig. E5i; Supplementary Tables S20–S23; Extended Data Table E1**). We also discuss this trade-off explicitly in the Discussion.

Third, we added the requested methodological clarifications. The **Methods / Experimental Configurations** now state the decoding parameters used in the main analyses and in the temperature-sensitivity analyses, specify that consistency was estimated from **five** independent stochastic runs per case unless otherwise noted, and describe the benchmark-specific Patient and Physician prompts. We also added implementation-sensitivity analyses for run count (N = 3, 5, 10), decoding temperature, and embedding model choice (MiniLM, MPNet, BGE) (**Extended Data Fig. E5a–h; Supplementary Tables S14–S19**). These analyses show that the main qualitative finding, namely the strong discriminative value of ConsistencyDx, is preserved, although absolute operating thresholds depend on implementation choices.

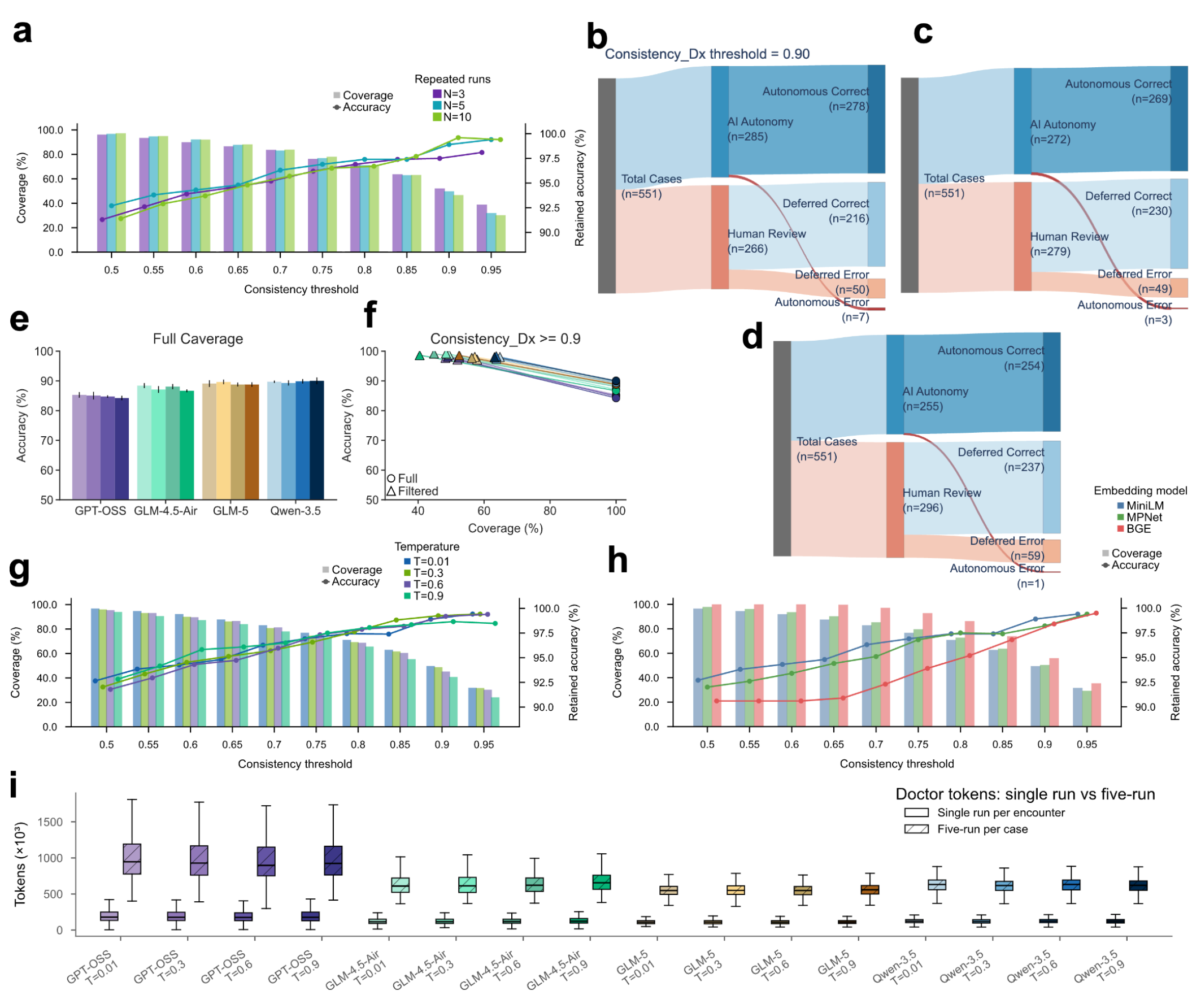

Extended Data Fig. E5 **|** Implementation sensitivity analyses of behavioral consistency

a, Bars show coverage, defined as the percentage of cases retained at each ConsistencyDx threshold, and lines show retained accuracy, defined as diagnostic accuracy among retained cases. Results are shown for N=3, N=5, and N=10 repeated runs. Coverage and retained accuracy were broadly similar across sampling counts, with limited threshold-specific differences; coverage diverged mainly at higher thresholds, where N=10 retained fewer cases than N=3 and N=5. **b–d,** Representative clinical triage workflows at a ConsistencyDx threshold of 0.90 for N = 3, N = 5, and N = 10, respectively. As the number of runs increased, the autonomy stream became smaller and more selective, with fewer missed errors but greater review burden. **e,** Diagnostic accuracy at full coverage across decoding temperatures for the evaluated on-premise models. Within each model, accuracy varied only modestly across temperatures, indicating that decoding temperature did not materially change the overall ranking of model performance. Colors from light to dark correspond to temperatures of 0.01, 0.3, 0.6 and 0.9, respectively. **f,** Coverage–accuracy operating points for all evaluated models at full coverage (circles) and after consistency-based retention with ConsistencyDx ≥ 0.9 (triangles) across decoding temperatures. Increasing temperature produced a modest reduction in coverage at the retained-set operating point while retained-case accuracy remained high, with the largest shift observed for GLM-4.5-Air. **g,** Bars show coverage and lines show retained accuracy across ConsistencyDx thresholds for T=0.01, 0.3, 0.6, and 0.9 using GLM-4.5-Air. Coverage denotes the percentage of cases retained above each threshold, while retained accuracy denotes diagnostic accuracy among retained cases. Higher temperatures generally reduced coverage at fixed thresholds, particularly at thresholds ≥0.80, whereas retained accuracy remained high with comparatively small differences across temperatures. **h,** Bars show coverage and lines show retained accuracy across ConsistencyDx thresholds for MiniLM, MPNet, and BGE embeddings. Coverage denotes the percentage of cases retained above each threshold, while retained accuracy denotes diagnostic accuracy among retained cases. Embedding choice substantially changed coverage at fixed thresholds, with BGE retaining more cases than MiniLM and MPNet across most thresholds, indicating that absolute consistency thresholds are not directly interchangeable across embedding models. **i,** Box plots show doctor-agent token use per encounter for single-run inference and per case for five-run consistency inference across model families and decoding temperatures. Token counts are shown in thousands. For single-run inference, each point represents one encounter-level run; for five-run inference, each point represents the summed doctor–agent token used across five repeated runs for the same case. Colors denote model–temperature configurations, and hatched boxes indicate five-run inference. Across all configurations, median doctor–agent token use increased from 125.0k tokens for single-run inference to 636.4k tokens per case for five-run consistency inference, corresponding to an approximately 5.1-fold increase. GPT-OSS showed the highest token use, whereas GLM-5 showed the lowest.

Changes made:

- Added external benchmark evaluation on **VivaBench**.
- Added **token-use**, **runtime**, and **hardware** profiling for consistency-based inference.
- Expanded the Methods to clarify inference **parameters**, **prompt** design, and default **run count**.
- Added **sensitivity analyses** for repeated-run count, decoding temperature, and **embedding model** choice.

Point 2.4 *- Statistical rigor: confidence intervals and threshold selection*

Comment: - Statistics and treatment of uncertainties: Performance metrics are reported clearly, but statistical rigor could be improved. Confidence intervals for key metrics (e.g., AUC and coverage estimates) should be provided, and the process for selecting confidence thresholds should be clarified to avoid potential overfitting.

Response:

Thank you for this suggestion. We now provide confidence intervals for the key performance metrics throughout the revised manuscript. Specifically, bootstrapped 95% confidence intervals are reported for all ROC AUC estimates (**Supplementary Tables S6, S8 and S11**), and 95% Wilson confidence intervals are reported for threshold-based retained-set accuracy and coverage estimates in the selective-autonomy, VivaBench, and implementation-sensitivity analyses (**Supplementary Tables S9, S12 and S14–S19**).

We also clarified the role of the threshold analysis. Because this was a **retrospective** benchmark study, the threshold analyses were intended as descriptive operating-characteristic analyses over predefined threshold grids rather than as prospective tuning of a deployment threshold. We therefore do not interpret the highlighted operating points as optimized thresholds learned from the evaluation cohort. In the revised Methods and Results, we now state explicitly that thresholds were evaluated across a prespecified range to illustrate the accuracy–coverage trade-off, and that values such as ConsistencyDx ≥ 0.90 are presented as representative conservative operating points rather than fitted optima.

Changes made:

- Added **confidence intervals** for key AUC, accuracy, and coverage metrics.

- Clarified in the **Results** and **Discussion** that threshold analyses were **descriptive** operating-characteristic analyses over prespecified threshold grids, not threshold fitting procedures (e.g Lines 531-532).

***Point 2.5** - Claims regarding safe autonomous deployment overreach the evidence*

Comment: Conclusions: robustness and reliability: The results suggest that behavioral consistency correlates with prediction correctness and may help identify reliable AI outputs. However, the manuscript's broader claims regarding safe autonomous clinical deployment appear stronger than the evidence presented. The study remains a retrospective benchmark analysis, and additional validation would be required to support claims about real-world clinical reliability.

Response:

Thank you for the comments. We agree and have moderated these claims throughout the revised manuscript. In the revision, we no longer present the study as establishing safe autonomous clinical deployment in real-world practice. Instead, we frame the findings more precisely as evidence that behavioral consistency is a useful decision-time reliability signal in a retrospective benchmark setting and that it can support selective-autonomy simulations under controlled evaluation conditions.

We now state more explicitly that the present study is a retrospective benchmark analysis, and that additional validation on independent clinical datasets, prospective workflows, and real-world human oversight settings will be required before making claims about clinical deployment safety or reliability in practice.

Changes made:

- Moderated deployment-related claims in the **Abstract**, **Introduction**, **Results**, and **Discussion**.
- Revised the text to refer to selective-autonomy simulations and decision-time reliability assessment, rather than safe autonomous deployment in practice.

  "In summary, our findings support a practical framework for more trustworthy clinical agents in which institutionally governed deployment is paired with decision-time reliability estimation to support selective autonomy with explicit human escalation." (Lines 536-538)

- Expanded the **Discussion** to state explicitly that prospective and real-world validation are still required (e.g Lines 531-535).

Point 2.6 *- Suggested improvements*

Comment: How to strengthen the manuscript: -External or temporal validation using data outside the MIMIC ecosystem; -Quantification of computational cost and latency for repeated inference used in behavioral consistency; -Expanded physician adjudication with reporting of inter-rater agreement; -Analysis of the clinical severity of residual errors in the autonomous stream; -Additional robustness tests reflecting realistic clinical data shifts.

Suggested improvements: -Several additions could strengthen the manuscript: -External or temporal validation using data outside the MIMIC ecosystem; -Quantification of computational cost and latency for repeated inference used in behavioral consistency; -Expanded physician adjudication with reporting of inter-rater agreement; -Analysis of the clinical severity of residual errors in the autonomous stream; -Subgroup analyses to evaluate performance across patient demographics; -Additional robustness tests reflecting realistic clinical data shifts

Response:

Thank you for these constructive suggestions. We addressed several of these points directly in the revised manuscript.

First, to extend the evaluation beyond the MIMIC-derived benchmarks, we added an external benchmark analysis on the independent PubMed-derived **VivaBench** dataset and now report both benchmark-level performance and selective-autonomy behavior in a new Results subsection (**Extended Data Fig. E4; Supplementary Tables S10–S13**). We make clear, however, that this broadens the evaluation setting but does not replace validation on independent clinical datasets or in prospective workflows.

Second, we now quantify the computational implications of repeated-run consistency estimation. The revised manuscript reports full-benchmark **token usage** for single-run and five-run inference, controlled-subset **wall-clock runtime**, and deployment hardware requirements (**Extended Data Fig. E5i; Supplementary Tables S20–S23; Extended Data Table E1**). We also discuss this deployment trade-off explicitly in the Discussion.

Third, we expanded physician-based evaluation. In the primary MIRA-v2 analyses, the revised manuscript reports blinded physician adjudication with **inter-rater agreement** metrics, including

Gwet's AC1, with full protocol details and per-round agreement in the **Methods** and **Supplementary Tables S2–S5**.

Fourth, the main text now includes representative case review of **high-consistency discordant cases** in the confidence-state analysis (Fig. 4). To address the clinical severity and interpretation of residual errors in the autonomous stream beyond the primary benchmark, we added physician review of **high-consistency missed (autonomous) errors** on **VivaBench** (**Supplementary Table S13**). This review showed that these residual errors were heterogeneous. Some reflected timepoint mismatch between presentation-level reasoning and benchmark endpoints that were established only after later investigations or inpatient evolution. Others represented genuinely stable misdiagnoses with potential for harmful misdirection. In addition, at least some difficult residual errors appeared to depend on concealed or weakly observable clinical cues, which would be challenging even for human clinicians and are especially difficult for a conversational agent without access to non-verbal observation. We now summarize these patterns in the **Results** and discuss their implications more explicitly in the **Discussion**.

Table S13 | Physician qualitative review of high-consistency missed-error cases on ViviaBench.
Cases were selected from the Qwen-3.5 baseline configuration with ConsistencyDx ≥ 0.85 and LLM evaluator = false, corresponding to missed errors in the selective-autonomy simulations.

**Case ID**	**Model output**	**Benchmark endpoint**	**physician**	**Clinical assessment**	**Likely error type**	**Why high confidence?**	**Missing or later-available evidence**	**Short note**
1	Multiple myeloma	Primary hyperparathyroidism due to parathyroid adenoma	A	Clinically meaningful error; no partial credit	Stable but incorrect reasoning	Anchoring on a familiar high-calcium/CRAB-like syndrome	PTH testing	Model converged on a plausible but incorrect common diagnosis and failed to exclude hyperparathyroidism.
2	Supracondylar fracture of left humerus	Reset osmostat	A	Partially correct but missed main diagnostic problem	Timepoint mismatch / benchmark ambiguity	Locked onto the presenting complaint	Broader work-up for hyponatremia	The fracture was real, but the clinically relevant diagnostic problem was the cause of hyponatremia.
3	POEMS syndrome	Metallosis with systemic cobalt/chromium toxicity	A	Clinically meaningful error; no partial credit	Stable but incorrect reasoning	Syndrome-pattern matching to POEMS features	Metal ion testing	Model overcommitted to a rare but superficially matching syndrome without excluding metallosis.

4	Multiple myeloma	Microscopic polyangiitis	A	Clinically meaningful error; no partial credit	Stable but incorrect reasoning	Anchoring on CRAB-like features suggestive of myeloma	Broader biological testing and ANCA-focused work-up	Model favored a common myeloma pattern instead of identifying ANCA-associated vasculitis.
5	Community-acquired pneumonia	Granulomatosis with polyangiitis	A	Clinically meaningful error; no partial credit	Timepoint mismatch / benchmark ambiguity	Bias toward common first-presentation etiology	ANCA testing and fuller systemic evaluation	Model treated the presentation as CAP and did not escalate the vasculitis differential.
6	Thyrotoxic periodic paralysis	Factitious disorder imposed on self / salbutamol overdose	A	Clinically meaningful error; no partial credit	Stable but incorrect reasoning	Classical syndrome fit despite incomplete confirmation	Thyroid-function testing; recognition of concealed self-induced cause	Model committed to a plausible syndrome, but the true diagnosis required recognizing concealed self-harm behavior.
7	IgA nephropathy	Hemolytic anemia secondary to SARS-CoV-2 infection	A	Clinically meaningful error; no partial credit	Stable but incorrect reasoning	Anchoring on apparent hematuria in a young patient	Recognition that pigment represented hemoglobinuria from hemolysis	Model interpreted the urinary finding as renal bleeding rather than hemolysis-related pigmenturia.
8	Acute pericarditis (model suspicion)	Tuberculosis	B	Reasonable initial diagnosis; partial credit likely warranted	Timepoint mismatch / benchmark ambiguity	Model identified a pathognomonic acute syndrome relevant at presentation	Delayed tuberculosis testing and later etiologic confirmation	In an acute setting, flagging pericarditis was judged more important than inferring tuberculosis before delayed test results returned.
9	STEMI constellation	Takotsubo syndrome	B	Reasonable initial diagnosis; partial credit likely warranted	Timepoint mismatch / benchmark ambiguity	Model captured the actionable presenting syndrome	Coronary angiography and later identification of apical ballooning	STEMI was judged the correct presentation-level diagnosis; Takotsubo became diagnosable only after CAG.
10	Lung abscess / severe pneumonia-type process	Autoimmune disease diagnosed later in admission	B	Reasonable initial diagnosis; partial credit likely warranted	Timepoint mismatch / benchmark ambiguity	Model prioritized the admission-worthy acute process	Later history (epistaxis) and delayed autoimmune synthesis	Model appropriately flagged an admission-level pulmonary process; the autoimmune diagnosis emerged only after later clinical evolution.

Fifth, we have added subgroup analyses stratified by patient demographics. Specifically, the revised figure now reports the age and sex composition of each benchmark cohort (Extended Data Fig. E1a,b) and model performance across age and sex subgroups (Extended Data Fig. E1c). These analyses show that performance heterogeneity was more apparent across age groups than across sex groups. In MIRA-v2, accuracy decreased from 91.8% in the 18-39 age group to 79.7% in the >=65 group. In CDM, accuracy similarly decreased from 88.2% in the 18-39 group to 72.5% in the >=65 group. In VivaBench, the pediatric subgroup had lower accuracy than the adult subgroups. Sex-stratified performance was comparatively similar in MIRA-v2 and VivaBench, while CDM showed a modest difference between female and male cases.

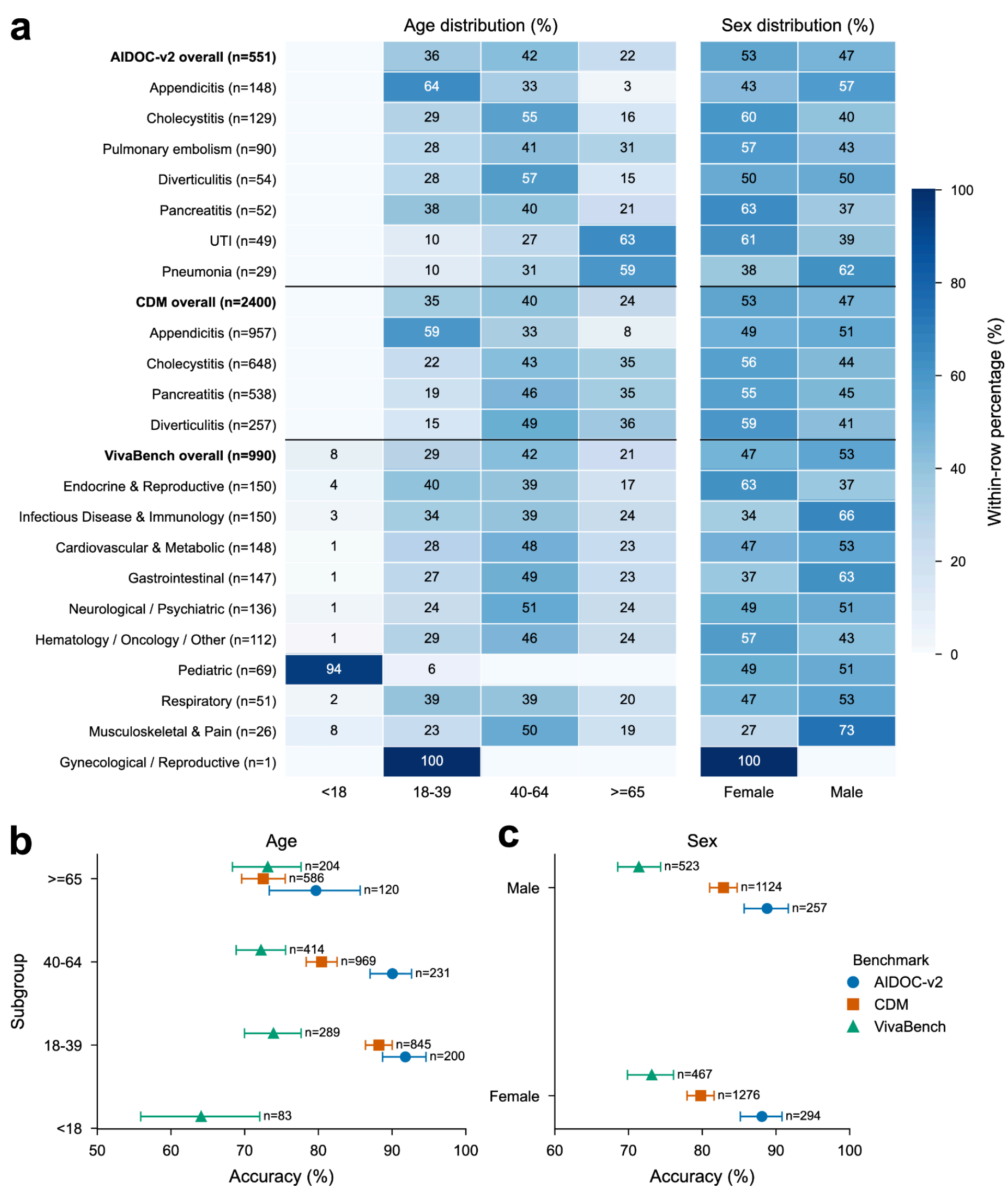

Fig. E1 | Benchmark demographic composition and subgroup diagnostic accuracy.
a, Heatmaps showing age-group (a) and sex (b) distributions for each benchmark overall and by disease category or specialty group. Rows indicate benchmark-level or subgroup-level cohorts, with case counts shown in parentheses. Cell values denote within-row percentages. Age groups were defined as <18, 18-39, 40-64 and >=65 years; sex labels were harmonized across datasets. **(b,c)** Diagnostic accuracy stratified by age group (b) and sex (c). Points show mean accuracy across repeated runs and horizontal bars show case-level bootstrap 95% confidence intervals; labels indicate subgroup sample sizes. Performance varied more by age than by sex, with lower accuracy in older patients in MIRA-v2 and CDM and lower accuracy in pediatric cases in VivaBench.

Sixth, the manuscript already included an **induced informational-scarcity perturbation test**, which we retain as a robustness analysis. In the revision, we further strengthened the robustness evaluation by adding sensitivity analyses for repeated-run count (N = 3, 5, 10), decoding temperature, and embedding model choice (**Extended Data Fig. E5; Supplementary Tables S14–S19**).

Changes made:

- Added external benchmark evaluation on **VivaBench**.
- Added **token-use, runtime, and hardware profiling** for consistency-based inference.
- Expanded physician adjudication reporting with **inter-rater agreement** metrics (**Supplementary Table S4**).
- Retained and clarified representative **MIRA-v2** case review of high-consistency discordant cases (**Fig. 4**).
- Added physician analysis of **VivaBench** residual missed errors in the autonomous stream.
- Added sensitivity analyses for **run count**, **decoding temperature**, and **embedding model** choice, while retaining the original informational-scarcity perturbation analysis.
- Added subgroup analyses stratified by patient demographics (**Extended Data Fig. E1**).

Point 2.7 *- References and context*

Comment: References and credit to previous work: The manuscript generally cites relevant literature. However, discussion of uncertainty estimation could reference related work on self-consistency reasoning in language models.

Context: This manuscript addresses an important and timely problem in clinical AI: how to deploy autonomous or semi-autonomous diagnostic agents in healthcare settings that require strong governance, reliability, and human oversight. The focus on on-premise deployment and confidence-based selective autonomy is clinically relevant, as institutions increasingly seek AI systems that are both high-performing and operationally trustworthy. The manuscript is generally well-positioned within the growing literature on clinical LLM agents, although the claims would benefit from clearer positioning as a retrospective benchmark study rather than as a validated real-world deployment framework.

Response:

Thank you for the comments. We agree and have revised the manuscript accordingly. In the revised text, we expanded the discussion of uncertainty estimation to better situate our framework relative to prior work on self-consistency reasoning in language models, semantic uncertainty estimation, and uncertainty-aware diagnosis. Specifically, we now discuss the relationship of our approach to self-consistency-style reasoning and semantic-entropy-based uncertainty estimation (**Wang et al. 2022**; **Farquhar et al., Nature 2024, and Zhou et al. 2025**), and we clarify that our contribution is not a new decoding or answer-selection rule, but the evaluation of cross-run semantic consistency as a decision-time reliability signal within a multi-step clinical agent workflow, alongside internal-likelihood and language-based measures.

Consistent with the broader point, we also moderated the framing of the manuscript to position it more clearly as a retrospective benchmark study rather than as a validated real-world deployment framework.

Changes made:

- Expanded the **Introduction** to discuss self-consistency reasoning, semantic uncertainty estimation, and uncertainty-aware diagnostic systems (Lines 93-102).
- Clarified in the **Methods** and **Discussion** how our consistency-based measure differs from prior self-consistency and uncertainty-estimation approaches.
- Moderated the overall framing of the manuscript to emphasize the **retrospective** benchmark setting.

Reviewer #3 - Machine Learning

***Point 3.1** - Limited new insight; text-only modality*

Comment: - The authors build an on-prem agent using open-source LLMs and an existing framework for diagnostic decision making. The Physician agent interacts with the patient agent to solicit information and eventually make a decision. The authors compare the discriminative performance of different 'confidence signals' across two benchamrks using MIMIC-IV. They then simulate clinician workflows that incorporate the agent when highly confident. Overall this was a significant effort, however the work falls short in many places and the data do not support the conclusions. This is not ready for publication.

Response:

Thank you for this candid overall assessment and for recognizing the substantial effort behind the study design. We agree that the original manuscript did not yet support some of its broader claims strongly enough. In response, we have substantially revised the manuscript to better align the conclusions with the evidence.

In particular, we have moderated the framing throughout to position the work as a retrospective benchmark study of decision-time reliability and selective-autonomy simulation, rather than as a validated real-world deployment framework. We also strengthened the evidence base by adding an external benchmark analysis on VivaBench, expanding methodological transparency and statistical reporting, adding computational and implementation-sensitivity analyses, and extending physician review of residual errors in the autonomous stream. Together, these revisions were made specifically to address the concern that the original data did not fully support the scope of the conclusions.

We hope the revised manuscript is now substantially stronger, both in evidentiary support and in precision of interpretation.

On-Premise vs. Cloud-Based Solutions

***Point 3.2** - Limited new insight; text-only modality*

Comment: The authors suggest they have demonstrated that an on-prem agent architecture can address trust gaps. As presented, though, the evidence mainly shows that locally run open-source models/tools can achieve similar performance to cloud-based proprietary models on two tasks drawn from a single dataset (MIMIC-IV).

a) *-Because there are already many benchmarks comparing open-source (that can be run locally) and proprietary models, it's not clear to me that this adds substantial new insight to that broader conversation.*
b) *-In addition, the evaluated models are entirely text-based. Since many diagnoses (e.g., pneumonia) often rely heavily on imaging data, this limits the clinical utility and generalizability of the conclusions.*

Response:

Thank you for the comments. We agree that the original framing was too broad. In the revised manuscript, we no longer present the study as showing that on-premise architecture, by itself, resolves the trust gap. Rather, we now frame the main contribution more precisely: the study provides a clinically motivated operational framework in which institutionally governed deployment, decision-time reliability estimation, and selective-autonomy routing can be evaluated jointly within an autonomous diagnostic workflow. The cloud comparison is therefore retained as a matched benchmark reference in this specific setting, not as the primary contribution or as a general statement about open versus proprietary models.

We also agree that the present study is limited to a **text-based diagnostic setting**, and we now state this more explicitly in the revised Discussion. At the same time, we clarify that this should be understood as a **scope limitation of the present evaluation**, not as a limitation of the framework in principle. The framework is modular and could in future incorporate modality-specific components, including multimodal or vision-language models, as these capabilities mature. However, native image interpretation addresses a distinct clinical and methodological problem from the text-mediated internal-medicine reasoning workflow evaluated here, and in many settings would overlap with specialist tasks such as radiology rather than simply extending the present task. We therefore interpret the current results as evidence for a **modular text-first clinical agent framework** for history-taking, tool-mediated evidence gathering, and decision-time reliability assessment, rather than as a claim that text-only agents are sufficient for all diagnostic tasks.

Changes made:

- Moderated the manuscript's framing to avoid implying that on-premise architecture alone resolves clinical trust gaps.
- Repositioned the main contribution as the joint evaluation of governed deployment, reliability estimation, and selective autonomy in a clinical agent workflow. *(Lines 536-541)*

- Expanded the **Discussion** to state explicitly that the present evaluation is text-based.
- Clarified that the framework is modular and could accommodate **future modality-specific or multimodal extensions**, while noting that native image interpretation raises a distinct clinical subproblem.

  "Second, the present evaluation was limited to a text-based diagnostic setting. While the framework is modular and could in future incorporate modality-specific components, including multimodal or vision-language models, native image interpretation represents a distinct clinical and methodological subproblem rather than a simple extension of the text-mediated internal-medicine reasoning workflow studied here. " (Lines 517-521)

Integrating Confidence Signals

***Point 3.3** - Generalizability across agents and datasets; risk of undetected failures*

Comment: The paper also argues that interpretable confidence signals help address trust gaps. I think this is a promising direction, but several aspects of the current evaluation make the claim feel insufficiently supported:

a) -The experiments rely on a single LLM/agent and datasets; how do the observations generalize across agents? across datasets?

Response:

Thank you for the comments. We agree that the original manuscript did not support broad cross-agent or cross-dataset generalization claims. In the revised manuscript, we have moderated this framing and now state explicitly that the principal confidence analyses were conducted on one primary agent configuration and are therefore not presented as universal across LLMs or agent architectures.

To strengthen the evidence, we added two forms of analysis. First, we now report benchmark results for additional open-weight models in the same agent framework, showing that competitive on-premise diagnostic performance is not unique to a single model (**Fig. 2a**). Second, we added an external benchmark evaluation on the independent PubMed-derived VivaBench dataset, where ConsistencyDx again emerged as the strongest evaluated signal and consistency-based triage again showed a graded accuracy–coverage trade-off (**Extended Data Fig. E4**). We also added implementation-sensitivity analyses showing that the main qualitative behavior of the consistency signal is preserved across variation in run count, decoding

temperature, and embedding model, although absolute operating thresholds are implementation-dependent (**Extended Data Fig. E5**).

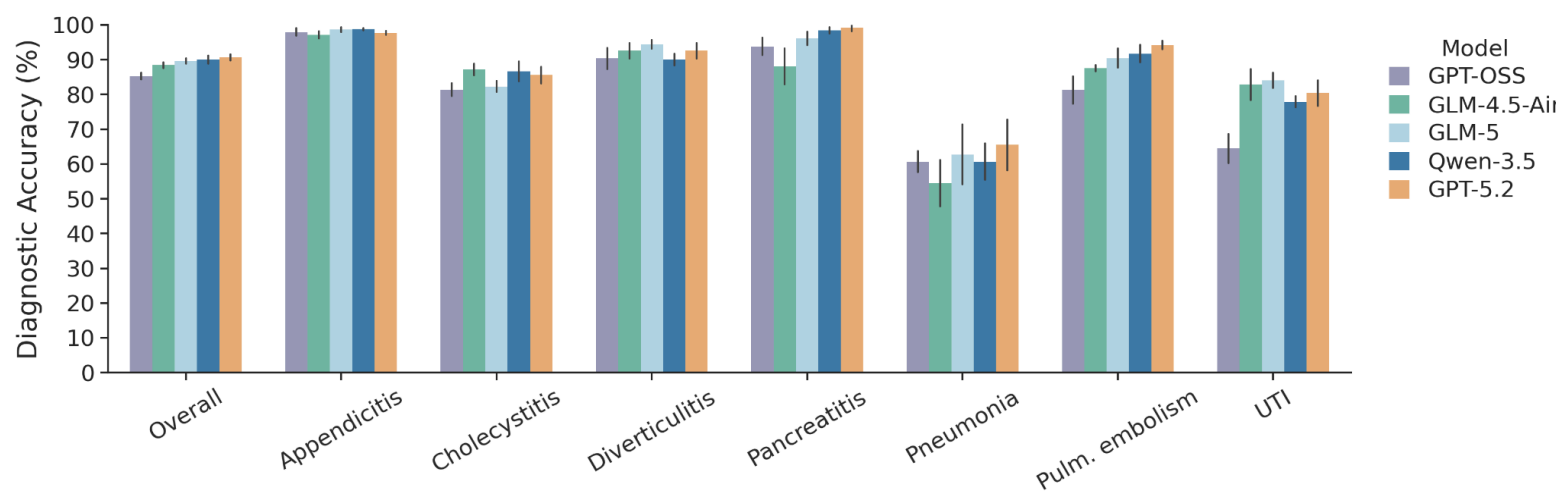

Fig. 2**a,** Diagnostic accuracy of four on-premise open-weight models (GLM-4.5-Air, GLM-5, Qwen-3.5, GPT-OSS) and a cloud baseline (GPT-5.2) across seven MIRA-v2 diseases. All systems used the same agent architecture and evaluation pipeline, differing only in the underlying LLM. For each model, accuracy is reported at the best-performing temperature setting; sensitivity to temperature is analyzed in the Implementation Sensitivity Analyses section.

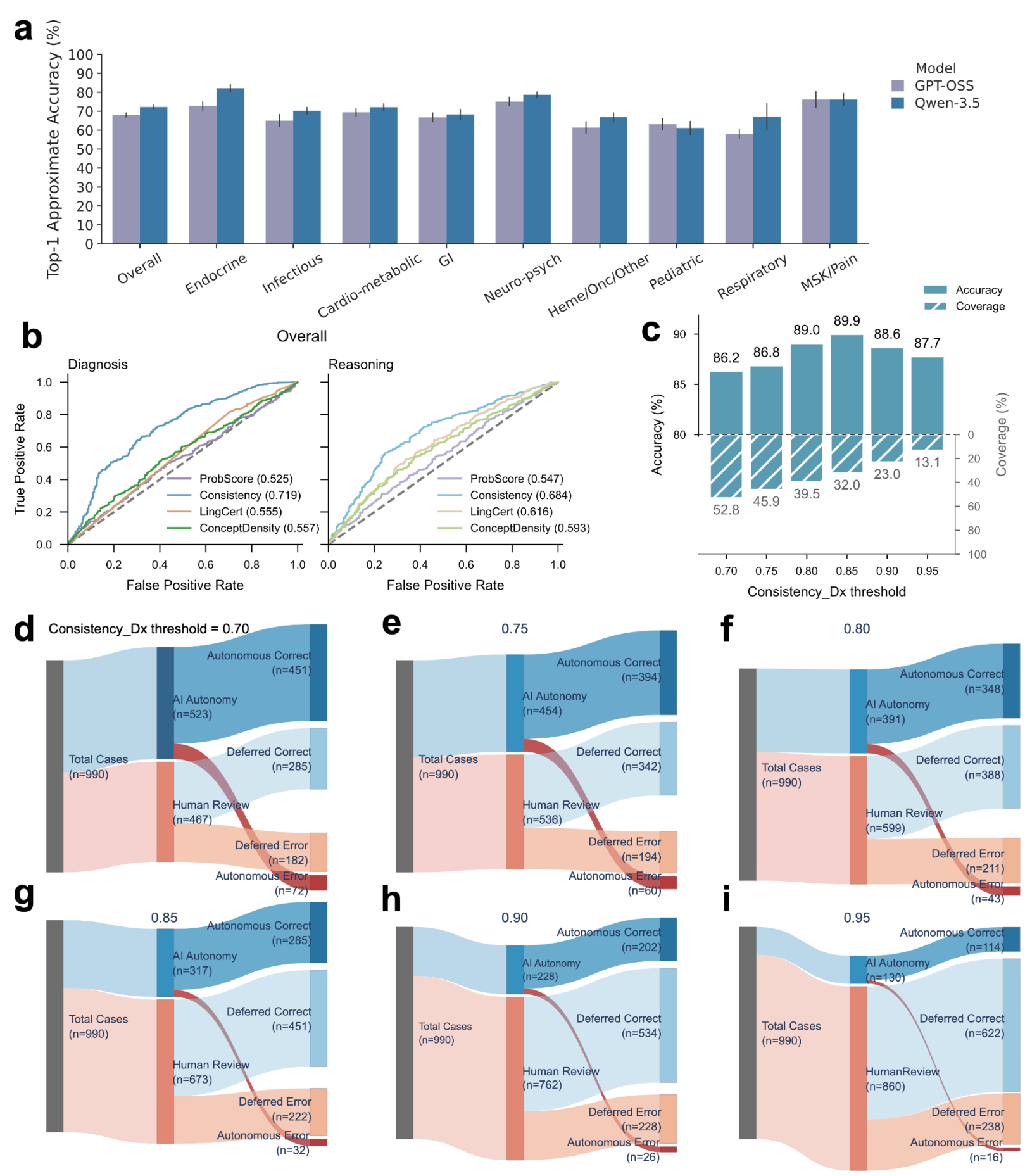

Fig. E4 | External benchmark evaluation and triage simulation on ViviaBench.

a, Because VivaBench is derived from physician-curated PubMed case reports and was designed to evaluate sequential reasoning under uncertainty, absolute performance is not directly comparable to the MIMIC-derived benchmarks. Top-1 approximate accuracy on the ViviaBench benchmark, shown overall and across 10 specialty groups for the evaluated open-weight models. Overall accuracy was 67.96 ± 1.09 for GPT-OSS and 72.22 ± 0.92 for Qwen-3.5. **b,** ROC analysis of reliability-related metrics on ViviaBench. ConsistencyDx showed the highest discrimination of correctness among the evaluated metrics (AUC = 0.719), exceeding ConsistencyR (AUC = 0.684), whereas probability-based and linguistic/concept-density metrics showed weaker discrimination. **c,** Accuracy–coverage analysis for consistency-based case retention on ViviaBench. Accuracy was computed among retained cases, and coverage was defined as the proportion of all cases retained at each ConsistencyDx threshold. Within the evaluated threshold range, the highest retained-set accuracy was observed at a threshold of 0.85 (89.9% accuracy at 32.0% coverage). **d–i,** Selective-autonomy simulations on ViviaBench across increasing ConsistencyDx thresholds (0.70, 0.75, 0.80, 0.85, 0.90 and 0.95). Cases meeting the threshold were routed to AI autonomy and the remaining cases to human review. Flows show autonomous correct, deferred correct, deferred error, autonomous error, illustrating the trade-off between automation coverage and residual risk as the threshold becomes more stringent.

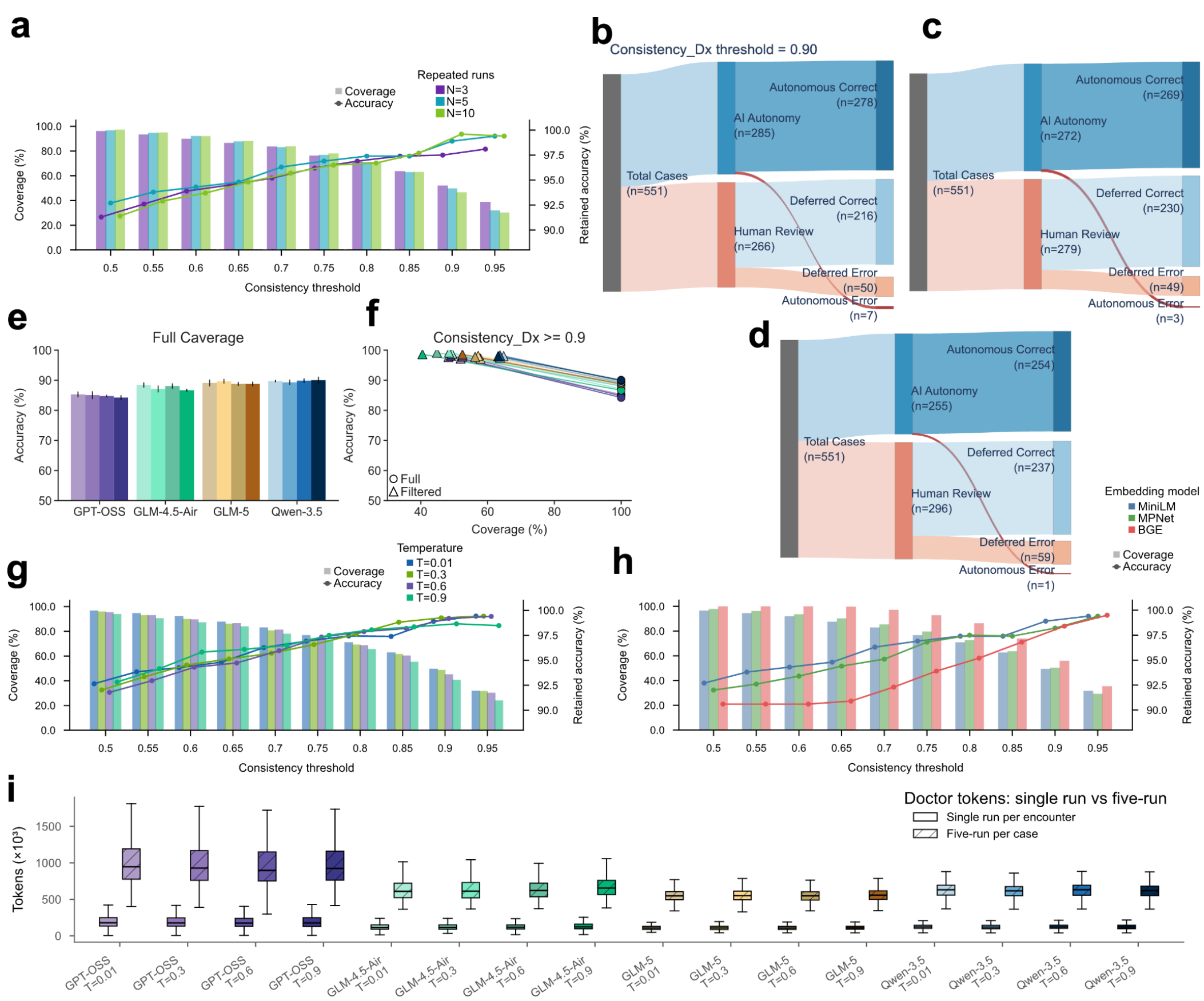

Extended Data Fig. E5 | Implementation sensitivity analyses of behavioral consistency

a, Bars show coverage, defined as the percentage of cases retained at each ConsistencyDx threshold, and lines show retained accuracy, defined as diagnostic accuracy among retained cases. Results are shown for N=3, N=5, and N=10 repeated runs. Coverage and retained accuracy were broadly similar across sampling counts, with limited threshold-specific differences; coverage diverged mainly at higher

thresholds, where N=10 retained fewer cases than N=3 and N=5. **b–d,** Representative clinical triage workflows at a ConsistencyDx threshold of 0.90 for N = 3, N = 5, and N = 10, respectively. As the number of runs increased, the autonomy stream became smaller and more selective, with fewer missed errors but greater review burden. **e,** Diagnostic accuracy at full coverage across decoding temperatures for the evaluated on-premise models. Within each model, accuracy varied only modestly across temperatures, indicating that decoding temperature did not materially change the overall ranking of model performance. Colors from light to dark correspond to temperatures of 0.01, 0.3, 0.6 and 0.9, respectively. **f,** Coverage–accuracy operating points for all evaluated models at full coverage (circles) and after consistency-based retention with ConsistencyDx ≥ 0.9 (triangles) across decoding temperatures. Increasing temperature produced a modest reduction in coverage at the retained-set operating point while retained-case accuracy remained high, with the largest shift observed for GLM-4.5-Air. **g,** Bars show coverage and lines show retained accuracy across ConsistencyDx thresholds for T=0.01, 0.3, 0.6, and 0.9 using GLM-4.5-Air. Coverage denotes the percentage of cases retained above each threshold, while retained accuracy denotes diagnostic accuracy among retained cases. Higher temperatures generally reduced coverage at fixed thresholds, particularly at thresholds ≥0.80, whereas retained accuracy remained high with comparatively small differences across temperatures. **h,** Bars show coverage and lines show retained accuracy across ConsistencyDx thresholds for MiniLM, MPNet, and BGE embeddings. Coverage denotes the percentage of cases retained above each threshold, while retained accuracy denotes diagnostic accuracy among retained cases. Embedding choice substantially changed coverage at fixed thresholds, with BGE retaining more cases than MiniLM and MPNet across most thresholds, indicating that absolute consistency thresholds are not directly interchangeable across embedding models. **i,** Box plots show doctor-agent token use per encounter for single-run inference and per case for five-run consistency inference across model families and decoding temperatures. Token counts are shown in thousands. For single-run inference, each point represents one encounter-level run; for five-run inference, each point represents the summed doctor–agent token used across five repeated runs for the same case. Colors denote model–temperature configurations, and hatched boxes indicate five-run inference. Across all configurations, median doctor–agent token use increased from 125.0k tokens for single-run inference to 636.4k tokens per case for five-run consistency inference, corresponding to an approximately 5.1-fold increase. GPT-OSS showed the highest token use, whereas GLM-5 showed the lowest.

We therefore revised the manuscript to frame these findings as evidence for the portability of the observed pattern within the evaluated settings, rather than as proof of full cross-agent or cross-dataset generalization.

Changes made:

- Moderated claims about **cross-agent** and **cross-dataset generalization**.
- Added additional open-weight **model** results within the same agent framework.
- Added external benchmark evaluation on **VivaBench**.
- Added **implementation-sensitivity analyses** for run count, decoding temperature, and embedding model choice.

---

b) *-The experiments rely on a single test set. Under distribution shift, models can be confident but wrong; communicating confidence while being wrong could harm trust. In the proposed design, agents act autonomously on "confident" cases, meaning clinicians might never notice this failure mode. That seems risky; some clinical oversight in all cases (even if only via random sampling) may be important.*

Response:

We agree that this is an important concern. In the revised manuscript, we have moderated the framing of selective autonomy accordingly. We no longer present confidence-based routing as eliminating the need for oversight, nor do we equate high-confidence retention with guaranteed safety under distribution shift. Instead, we frame the proposed approach as a **risk-stratification mechanism** in a retrospective benchmark setting, in which confidence signals may help identify a subset of cases with lower observed error rates while preserving explicit escalation pathways for the remainder.

Several analyses in the revised manuscript now speak directly to this concern. First, the induced informational-scarcity perturbation test shows that models can remain relatively confident while becoming less accurate, particularly for probability-based signals, reinforcing that confidence can fail under distribution shift. Second, the main text already included representative review of **high-consistency discordant cases** in the primary MIRA-v2 analysis (**Fig. 4**), illustrating that some apparently high-confidence errors reflected benchmark-label limitations rather than uniformly unsafe reasoning. Third, the external VivaBench evaluation and physician review of **high-consistency missed errors (Supplementary Table S13**) show that residual errors

persist even in the autonomous stream and are heterogeneous in clinical significance. Together, these analyses led us to emphasize more clearly that selective autonomy should be understood as **simulated confidence-based triage**, not as a substitute for ongoing clinical governance.

We agree that practical deployment would likely require additional safeguards beyond the benchmark setting, including some form of continuing oversight, audit, sampling-based review, or prospective monitoring of retained cases. We now state this more explicitly in the Discussion.

Changes made:

- Moderated the manuscript's framing of selective autonomy to avoid implying that high-confidence cases require no further oversight.
- Added emphasis in the **Discussion** that confidence-based routing does not eliminate residual risk, especially under distribution shift.

  "At the same time, confidence-based routing should be understood as a mechanism for risk stratification rather than as a substitute for continuing clinical governance, including oversight, audit, sampling-based review, or prospective monitoring of retained cases." (Lines 475-478)

- Linked this limitation to the stress-test results, the primary MIRA-v2 case review, and the physician review of missed errors in the autonomous stream on **VivaBench**.

---

c) *-There was little to no justification for the various confidence metrics. Where did these come from? Moreover, why do we expect them to measure different things? ProbScore and Consistency should be highly correlated based on their underlying formulation - the closer the probability is to 0.5 the more randomness/inconsistency I'll observe in the output*

Response:

We agree that the original manuscript did not justify the metric design clearly enough. In the revised manuscript, we therefore expanded the Methods and Introduction to explain both the provenance and the intended role of each metric family. Specifically, the framework is now motivated as a comparison of three complementary, reference-independent views of inference-time reliability: internal likelihood, expressed uncertainty in language, and behavioral stability across repeated stochastic runs. We also added discussion of the relevant prior

literature on self-consistency reasoning, semantic uncertainty estimation, and uncertainty-aware diagnosis to position the framework more explicitly.

We further clarified why these measures were not assumed to be interchangeable. ProbScore is a single-run token-level likelihood summary, whereas Consistency is a cross-run semantic stability measure computed over repeated stochastic executions. Although both are influenced by stochastic generation, they are not equivalent by construction. In multi-turn agent workflows, small generation differences can lead to different reasoning trajectories or tool-use paths without being fully captured by the average token-level likelihood of any single output. Second, as another reviewer correctly pointed out, token-level probability may remain high for syntactically fluent outputs even when they are not factually well grounded. We therefore no longer describe ProbScore as a direct measure of diagnostic certainty, but rather as an internal likelihood proxy. This distinction is also supported by our results: internal probability and diagnostic consistency were only moderately correlated ($r = 0.51$), showed low multicollinearity in joint analysis (all VIF < 5; **Supplementary Table S7**), and behaved differently under both perturbation and triage analyses. In particular, under induced informational scarcity, ProbScoreDx often remained relatively high despite degraded accuracy, whereas ConsistencyDx both preserved discrimination and shifted downward in absolute value. We therefore retain ProbScore not as a standalone confidence measure, but as a comparative internal signal against which behavioral and language-based measures can be evaluated.

Finally, we have revised the framing of ConceptDensityR as well. Because it showed an inverse association with correctness, it is now described explicitly as an inverse / risk-style indicator rather than as a positive confidence signal.

Changes made:

- Expanded the **Introduction** and **Methods** to justify the origin and intended interpretation of each metric family (Lines 93-102; 810-814).

  “This design was informed by prior work on self-consistency reasoning, semantic uncertainty estimation, and uncertainty-aware diagnostic systems[26,28,41], but differs in that the present study evaluates these signals jointly within an autonomous clinical agent workflow and computes them separately for both the final diagnostic output (Dx) and the accompanying reasoning trace (R).” (Lines 810-814)

- Clarified that **ProbScore** is an internal likelihood proxy, not a direct measure of diagnostic certainty.

“Because this metric is derived from token-level generation likelihood within a single run, it may reflect local fluency or model preference without guaranteeing factual correctness or calibration. We therefore treated ProbScore as one component of a broader reliability framework rather than as a sufficient confidence measure on its own.” (Lines 833-836)

- Added empirical support in the **Results** showing only moderate correlation and low multicollinearity between the retained metrics, together with distinct behavior under perturbation and triage analyses. (Lines 224-226)
- Reframed **ConceptDensityR** showed an inverse trend with correctness, but did not behave as a reliable confidence measure with higher values associated with incorrect reasoning traces. (Lines 454-457)

---

d) *-ProbScore normalizes for output length, but it still might disproportionately favor short outputs. Have the authors examined the relationship between output length and probabilistic score? (ConceptDensity may also be length-sensitive.)*

Response:

Thank you for these constructive suggestions. We performed a post-hoc length-sensitivity analysis. ProbScoreDx was negatively associated with diagnosis token count, indicating some length sensitivity, but it remained significantly associated with incorrectness after adjustment for diagnosis token count. ProbScoreR showed no meaningful association with reasoning token count and also remained significant after length adjustment. In contrast, ConceptDensityDx was strongly length-sensitive and lost its association with incorrectness after adjustment for diagnosis word count, whereas ConceptDensityR was not length-sensitive but remained non-significant after length adjustment. We therefore retained ProbScore as a probabilistic confidence metric but revised our interpretation of ConceptDensity metrics, treating them as exploratory content-structure measures rather than standalone confidence signals.

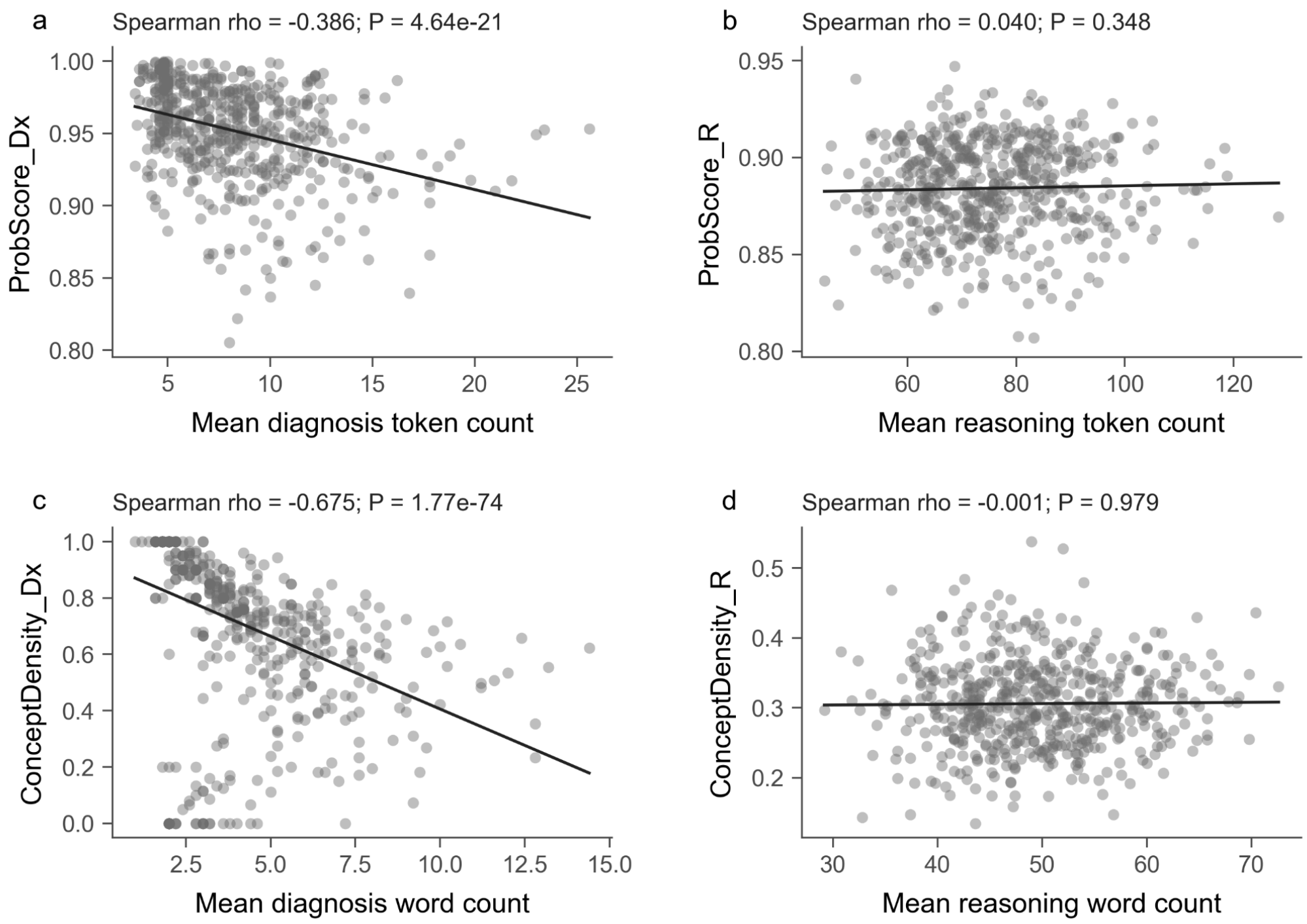

Response Fig. R1 | Post-hoc length-sensitivity analysis of probabilistic and concept-density metrics.

ProbScore was compared with the corresponding model-token count, whereas ConceptDensity was compared with the corresponding word count, reflecting each metric's native unit of calculation. ProbScoreDx showed a negative association with diagnosis token count (Spearman ρ = -0.386, P = 4.64 × 10^-21), whereas ProbScoreR showed no meaningful association with reasoning token count (ρ = 0.040, P = 0.348). ConceptDensityDx was strongly associated with diagnosis word count (ρ = -0.675, P = 1.77 × 10^-74), indicating substantial length sensitivity, whereas ConceptDensityR showed no association with reasoning word count (ρ = -0.001, P = 0.979). Lines indicate linear visual guides; statistical testing used Spearman correlation.

---

e) *-Discriminative performance of confidence signals is quantified using ROC analysis on a single test set per benchmark, which raises concerns about generalization. In Figure 3, are results combined across both benchmarks, and were patterns consistent across benchmarks? From the extended data, it appears relative discriminative performance varies substantially across diagnoses (e.g., pulmonary embolism vs. cholecystitis*

Response:

We agree that confidence-signal discrimination estimated on a single benchmark split should not be overinterpreted as evidence of broad generalization. We have clarified this point in the revised manuscript. In particular, Fig. 3 reports MIRA-v2 only (n = 551) under baseline

conditions; the results are not combined across benchmarks. We now state this explicitly in the Results and Methods.

We also agree that discriminative performance varied across diagnoses. In the revision, we therefore moved the detailed condition-level ROC analyses out of the main narrative and report them in **Extended Data Fig. E3**, where this heterogeneity can be inspected directly. The main text now presents the aggregate result more cautiously, namely that ConsistencyDx was the strongest overall signal on MIRA-v2, while the relative value of likelihood- and language-derived measures was context dependent across conditions.

To strengthen evidence beyond a single MIMIC-derived benchmark, we added an external benchmark analysis on **VivaBench**. Although absolute performance differed and heterogeneity remained, the main qualitative pattern was preserved: ConsistencyDx again showed the strongest discrimination among the evaluated metrics and supported a graded accuracy–coverage trade-off in selective-autonomy analyses. We have revised the manuscript to present this as support for portability across the evaluated settings, not as proof of generalization across benchmarks or diagnoses.

Changes made:

- Clarified that Fig. 3 is based on **MIRA-v2** only and does not combine benchmarks.
- Moved detailed disease-level ROC analyses to **Extended Data Fig. E3**.
- Added external benchmark evaluation on **VivaBench**.
- Moderated the text to emphasize overall patterns plus diagnosis-specific heterogeneity, rather than broad generalization claims.

---

f) -A key finding is that behavioral stability is a stronger indicator of diagnostic correctness than internal likelihood estimates. It's not clear whether this will generalize beyond this model and dataset.

Response:

We agree that this should not be presented as a universal claim beyond the evaluated settings. In the revised manuscript, we have moderated this point accordingly. The main finding is now framed more precisely as follows: within the primary MIRA-v2 agent configuration, behavioral

stability was a stronger indicator of diagnostic correctness than internal likelihood, and this pattern was supported, but not fully generalized, by the additional analyses.

To strengthen this point, we added two forms of support. First, we now report benchmark results for **additional open-weight models** within the same agent framework (**Fig. 2a**). Second, we added an external benchmark analysis on **VivaBench**, where ConsistencyDx again showed the strongest discrimination among the evaluated metrics and supported selective-autonomy triage (**Extended Data Fig. E4**). We also added **implementation-sensitivity analyses** showing that the qualitative behavior of consistency was preserved across variation in repeated-run count, decoding temperature, and embedding model choice, although absolute thresholds were implementation-dependent (**Extended Data Fig. E5**).

We therefore revised the text to present this result as evidence for the portability of the observed pattern across the evaluated settings, rather than as proof of broad cross-model or cross-dataset generalization.

Changes made:

- Moderated the corresponding claims in the **Results** and **Discussion**.
- Added supporting analyses across additional open-weight **models**, an external benchmark (**VivaBench**), and implementation-sensitivity settings.
- Clarified that the finding is setting-specific and does not establish universal generalization.

---

g) -All experiments are conducted in simulation, so it's hard to anticipate how deferring "easy" cases would affect real clinician workflow and decision-making. For example, only seeing difficult cases might increase fatigue and potentially errors.

Response:

We agree that the present study, being retrospective and simulation-based, cannot determine how selective-autonomy routing would affect real clinician workflow in practice. We therefore do not claim that deferring lower-risk cases would necessarily improve or worsen clinician performance, fatigue, or error rates. In the revised manuscript, we now state this limitation more explicitly.

At the same time, we note that the concern that reviewing a more concentrated set of difficult cases would necessarily increase burden is currently speculative. We therefore revised the Discussion to frame this as an open workflow question rather than as a directional conclusion. We also note that recent studies (van Buchem et al., 2024, Ma et al., 2025, You et al., 2025) of ambient AI documentation tools and clinical scribes have reported reductions in documentation time and subjective work burden, suggesting that AI systems can in some settings reduce, rather than increase, clinician workload. We mention this literature only as contextual support for the possibility that AI assistance may change burden in either direction; we do not treat it as direct evidence for the selective-autonomy workflow studied here.

Accordingly, the revised manuscript now emphasizes that prospective evaluation will be required to determine how confidence-based triage affects clinician workload, fatigue, review behavior, and safety in real deployment settings.

Changes made:

- Expanded the **Discussion** to state explicitly that workflow effects of selective autonomy cannot be inferred from retrospective simulation alone (Lines 475-478; 531-532).

  "At the same time, confidence-based routing should be understood as a mechanism for risk stratification rather than as a substitute for continuing clinical governance, including oversight, audit, sampling-based review, or prospective monitoring of retained cases." (Lines 475-478)

- Reframed clinician-burden effects as an open empirical question rather than a directional assumption.

  "Prospective studies and dedicated bias audits will be required to determine how these signals affect clinician reliance, review burden, safety outcomes and fairness across patient subgroups in real practice." (Lines 533-535)

---

h) -Since diagnosis is ultimately binary (disease vs. no disease), why not map the generated output to a set of binary labels so that "fuzzy" text matching isn't required?

Response:

We agree that structured label mapping is attractive when a task has a fixed closed label space. However, this was not appropriate for all benchmarks used here. In our setting, the agent produces free-text clinical diagnoses, and the relevant evaluation problem is not purely binary

"disease versus no disease," but whether the generated diagnosis is clinically equivalent, acceptably synonymous, or reasonably aligned with the benchmark endpoint.

For CDM, we retained the published fuzzy-matching protocol specifically to preserve comparability with the original benchmark and leaderboard. For MIRA-v2, we used the established LLM-as-judge protocol because rigid binary label mapping would under-credit clinically equivalent formulations and plausible alternative expressions. This concern is supported by our physician adjudication results, which showed that some automated "errors" reflected label limitations rather than clinically implausible outputs. For VivaBench, simple binary mapping was likewise inappropriate because the benchmark provides both a diagnosis list and an accepted differentials list, whereas the agent outputs a single free-text diagnosis. We therefore used a benchmark-adapted LLM-based adjudication procedure aligned to the benchmark's approximate top-1 evaluation principle rather than forcing all outputs into a rigid binary label space.

In short, structured binary mapping would be appropriate only if the benchmark task were defined as a closed-label classification problem; our evaluation instead targeted clinical equivalence and benchmark-consistent alignment of free-text diagnostic outputs under benchmark-specific scoring protocols.

Changes made:

- Stated more explicitly that CDM used published **fuzzy matching** for benchmark comparability, MIRA-v2 used **LLM-based** adjudication for clinical equivalence, and VivaBench used benchmark-adapted LLM adjudication because of its diagnosis-list / differential-list structure. **(Methods / Performance Evaluation / Automated Benchmark Scoring)**

Organization

Point 3.4 *- DDx Critic Agent buried; adversarial stress testing connection unclear; incomplete citations*

Comment: I found the paper difficult to follow in places.

a) *-Some results appear in the Methods section (e.g., performance with and without a critic agent). I actually found that comparison informative and interesting, but it felt buried in*

the methods/supplement and received little attention in the Introduction/Results/Discussion. I'd be interested to see these experiments expanded, including analysis of why the critic agent helps in some cases but not others.

Response:

Thank you for the comments. We agree that this component was under-emphasized in the original manuscript. In the revised version, we moved the DDx Critic Agent comparison out of the purely methodological background and now report it explicitly in the **Results** (On-Premise Agent Achieves Competitive Diagnostic Performance; **Extended Data Fig. E2**). We also added a brief explanation in the Discussion to clarify its role as a separate architectural extension rather than part of the main confidence-analysis pipeline.

At the same time, we chose not to expand this into a larger standalone analysis in the present revision, because the critic-agent configuration did not improve overall diagnostic accuracy and did not change the main conclusions regarding confidence estimation and selective autonomy. We therefore now present it as an informative secondary finding: the critic improved performance in some settings, most notably pneumonia, but did not yield a consistent net benefit across the benchmark. We agree that understanding when critique helps versus destabilizes otherwise strong reasoning is important, and we now identify this more explicitly as a direction for future work.

Changes made:

- Moved the critic-agent comparison into the **Results** and made it more visible in the manuscript (Lines 176-183).
- Added brief framing in the **Discussion**.

  "Third, a multi-agent critic extension showed heterogeneous condition-specific effects without improving overall performance, indicating that the circumstances under which critique is beneficial remain to be defined. The present study was not designed to determine when critique is beneficial versus destabilizing, and this remains an important question for future work." (Lines 522-526)

- Clarified that the critic-agent result is a secondary architectural finding, whereas the main manuscript focus remains confidence estimation and selective autonomy.

---

b) *-There are also a number of tangential experiments (relative to the central "trust gap" goal), such as adversarial stress testing. While interesting on their own, I wasn't fully convinced of their direct connection to the main research question or hypothesis as currently framed.*

Response:

We appreciate this point and have clarified the role of these analyses in the revised manuscript. We do not intend the stress-test experiments as tangential robustness exercises in isolation. Rather, they address a central part of the trust question: whether a proposed decision-time reliability signal continues to behave meaningfully when evidentiary grounding degrades. In this setting, the relevant issue is not only whether accuracy falls, but whether confidence signals also respond in a clinically sensible way or instead remain spuriously high.

We revised the Results and Discussion to make this connection more explicit. In particular, the perturbation analysis is now framed as a test of whether confidence signals preserve discriminative value and shift appropriately under reduced information, which directly informs their suitability for selective-autonomy triage. This distinction proved important in the revised results: ConsistencyDx remained discriminative and shifted downward in absolute value, whereas probability-based signals could remain relatively high despite degraded accuracy.

Changes made:

- Revised the framing of the **stress-test analyses** in the **Results** and **Discussion** to connect them more directly to the manuscript's central trust question. (Lines 262-265)
- Clarified that the perturbation experiments evaluate whether confidence signals remain meaningful under degraded evidentiary grounding, rather than serving as standalone robustness exercises. (Lines 262-265; 267-269; 274-275)

---

c) *-some of the citations are incomplete; despite the architecture being adapted from the MIRA framework, citations 28 points to a paper that doesn't seem to exist (at least a google search doesn't return anything); this is concerning given how much the paper relies on this framework*

Response:

Thank you for flagging this. The related benchmark/framework manuscript cited in the original submission was not publicly available at the time and was cited under its earlier name, MIRA.

We now refer to the related framework by its updated name, MIRA (Dyke Ferber et al., Nature, 2026, in press), and we make explicit that the present architecture was adapted from this related benchmark/framework manuscript rather than from an already established public reference. We also clarify in the Methods which architectural elements were inherited from the MIRA framework and which were modified in the current implementation. For editorial and reviewer context, we have included the most recent PDF version of the related manuscript.

TO-DO (important points across all Reviewers):

I. **Reanalysis of existing results (n=551)**
- [x] ~~Compute bootstrapped 95% confidence intervals for all reported AUC values~~
- [x] ~~Run DeLong tests for pairwise AUC comparisons, at minimum ConsistencyDx vs. ProbScoreDx~~
- [x] ~~Add N=3 and N=10 sensitivity analyses for the Consistency metric~~
- [x] ~~Add more metrics~~

II. **New reporting from existing infrastructure**
- [x] ~~Extract token usage per encounter from run logs~~
- [x] ~~Record wall-clock time per case for both single-run and 5-run consistency analyses~~
- [x] ~~Document GPU resource requirements for both the H200 and RTX A6000 setups~~

III. **Post-hoc analyses on existing data**
- [x] ~~Compare ConceptDensity scores in correct vs. incorrect traces against number of tool calls and evidence retrieved~~
- [x] ~~Examine the relationship between output length and ProbScore, and separately for ConceptDensity~~
- [x] ~~Report decoding parameters (temperature, top-p) used across all stochastic runs~~

IV. **Manuscript rewriting**
- [x] ~~Temper overreaching language in the abstract, introduction, and discussion~~
- [x] ~~Replace "matching GPT-5.2" with "approaching" or "comparable to"~~
- [x] ~~Remove or reframe "validated blueprint for the next generation of clinical AI"~~
- [x] ~~Tone down the generalizability statement in the Methods~~
- [x] ~~Restructure the confidence framework Results section to lead with ConsistencyDx as the primary finding~~
- [x] ~~Move interpretive statements from Results to Discussion~~
- [x] ~~Reframe ConceptDensity explicitly as a risk indicator rather than a confidence signal~~
- [x] ~~Shorten the regulatory discussion (HIPAA, GDPR, PIPEDA) to 3-4 sentences~~
- [x] ~~Resolve duplicate references (Refs 3/29 and Refs 17/30)~~

V. **Literature and citation work**
- [x] ~~Add and discuss Omar et al. (Nat Med, 2025) on sociodemographic bias amplification~~

- ☑ ~~Add and discuss Farquhar et al. (Nature, 2024) on semantic entropy~~
- ☑ ~~Add and discuss Taubenfeld et al. (ACL 2025) on confidence-weighted self-consistency~~
- ☑ ~~Add and discuss Zhou et al. (npj Digital Medicine, 2025) on ConfiDx~~
- ☑ ~~Add and discuss Dennstädt et al. (npj Digital Medicine, 2025) on on-premise LLM governance~~

VI. Dataset search

(running concurrently, does not block anything above)

- ☑ ~~Identify a suitable external or temporally held-out dataset outside the MIMIC ecosystem~~

Further Changes

1. In the course of revision, we corrected the description of the LLM-based evaluator used in the benchmark adjudication pipeline. The original submission referred to **GPT-4o**, whereas the evaluator actually used in the revised benchmark-scoring pipeline was **gemini-3.1-flash-lite-preview**. We selected this evaluator because, at the time of revision, it offered a favorable of both benchmarked capability and cost for large-scale adjudication relative to GPT-4o. The evaluator was run independently from the clinical agent and was used only for benchmark adjudication. This correction has now been made throughout the revised manuscript. It resulted in minor updates to some adjudication-based summary values, but did not change the main findings or conclusions of the study.

2. We also made a terminology update throughout the revised manuscript to maintain consistency with the latest version of the related benchmark/framework manuscript. Specifically, the benchmark/framework previously referred to as **AIDOC** is now referred to as **MIRA** (Dyke Ferber et al., Nature, 2026 (in press)). As this related manuscript remains under review and is not yet publicly available, we have included the most recent PDF version for editorial and reviewer reference. This naming update is terminological only and does not affect the experiments, results, or conclusions reported in the present manuscript.

Response to Reviewers

Manuscript NMED-A149668A: “On-Premise Medical AI Agents for Reliable Clinical Decision-Making”

We thank the editor and all reviewers for their careful, constructive, and encouraging comments. We are grateful that the revised manuscript was viewed as substantially strengthened and improved, and we appreciate the clear guidance on the remaining issues to be addressed before publication. We have revised the manuscript accordingly.

Reviewer #1

Point 1.1 *- Overall assessment of the revision*

Comment:

- Thank you for revising this paper. I think that the revision was clearly taken seriously - the additional analyses and expanded discussion are appreciated and genuinely improve the work.

- Overall, as said above, I think that this revision is a genuine improvement, and the authors engaged seriously with every point. The paper is - in fact - a novel and timely contribution to the field. Work on operational trust and decision-time reliability is needed at this stage of clinical AI deployment, and I think this paper makes a real contribution to that conversation. The four points above are specific and addressable, and I hope that the paper will be in very strong shape once they are considered. I appreciate the authors' work on this - it was a careful and substantive revision, and I thank them for taking the comments seriously.

Response:

Thank you very much for these encouraging comments. We appreciate your clear guidance on the four remaining points, and we have revised the manuscript further to address each of them directly.

Point 1.2 *- Age-gradient finding should be discussed directly*

Comment: - The demographic subgroup analyses added in revision (Extended Data Fig. E1) are a welcome addition. But I think the finding sitting in those panels is not a minor one. Accuracy drops from 91.8% in the 18-39 age group to 79.7% in the >=65 group on MIRA-v2, with a similar gradient in CDM. That is a 12-point performance gap in the patient population potentially carrying higher burden in ER . The limitation statement at lines 531-535 correctly acknowledges the absence of counterfactual demographic testing, and I appreciate that. But the Discussion does not engage with what the age gradient itself means for deployment safety and fairness. For a paper that explicitly frames a selective autonomy workflow, I think it is worth asking out loud: who

is most likely to be handled autonomously versus deferred, and does this gradient map onto a group already at higher clinical risk?

I suggest adding at least a paragraph in the Discussion that engages with the finding directly - not just scopes it as a limitation. The data are already there. This would be a Discussion revision only.

Response:

Thank you for this important comment. We agree that the age gradient should be discussed directly rather than treated only as a limitation. In the revised manuscript, we now address this finding explicitly in the **Discussion** as a deployment-relevant issue for both **safety** and **fairness**. Specifically, we now note that this age gradient is especially important in a selective-autonomy setting. A confidence-based routing policy must be evaluated not only by average retained accuracy, but also by which patients are more likely to be handled autonomously versus deferred.

To address this more directly, we also performed an **exploratory age-stratified post-hoc routing analysis** on **MIRA-v2** at the main ConsistencyDx ≥ 0.90 operating threshold. Older patients were retained substantially less often than younger adults (coverage 25.8% in ages >=65 versus 65.5% in ages 18–39), and were correspondingly more likely to be deferred for clinician review (review fraction 74.2% versus 34.5%; **Supplementary Table S38**). We do not interpret this as a causal or generalizable fairness analysis. This analysis was limited to one benchmark, and we did not perform counterfactual demographic perturbation or prospective workflow evaluation. Instead, this analysis supports the point made in the **Discussion**, that subgroup performance gradients may translate into differential autonomous-versus-deferred routing in practice.

Accordingly, the revised **Discussion** now frames the age gradient as an important deployment consideration: if performance is systematically reduced in older patients, this could map onto a population already carrying higher clinical risk, with implications for both **safety** and **fairness**. At the same time, we make clear that the present subgroup and routing analyses remain **descriptive**, and that dedicated prospective validation and bias auditing are still required.

Changes made:

- Added **Supplementary Table S38**, reporting an exploratory age-stratified post-hoc routing analysis on **MIRA-v2** at the main ConsistencyDx ≥ 0.90 threshold.

  Table S38 | Age-stratified routing outcomes at ConsistencyDx ≥ 0.9 on MIRA-v2. Exploratory post-hoc analysis of selective-autonomy routing at the main ConsistencyDx operating threshold used in the primary MIRA-v2 analysis. For each age group, the table reports retained cases, coverage, deferred cases, review fraction, residual autonomous-stream errors, deferred-stream errors, and retained accuracy. This analysis is descriptive and was not used for threshold optimization or causal interpretation of subgroup differences.

Age Group	Retained n	Coverage	Deferred n	Review fraction	Autonomous errors	Deferred errors	Retained accuracy

18-39	131	0.655	69	0.345	1	10	0.992
40-64	110	0.476	121	0.524	2	18	0.982
≥65	31	0.258	89	0.742	0	21	1.000

- Added a new **Discussion paragraph** interpreting the observed age gradient as a deployment-relevant issue for **safety** and **fairness**, and noting that exploratory age-stratified routing results on **MIRA-v2** were directionally consistent with differential autonomous-versus-deferred routing across age groups.

  "An additional deployment-relevant finding was the age gradient observed in the subgroup analyses. On both MIRA-v2 and CDM, diagnostic accuracy was lower in older age groups than in younger adults, whereas sex-stratified differences were smaller. This matters for selective autonomy because a routing concerns not only average retained accuracy, but also which patients are more likely to be handled autonomously versus deferred. Exploratory age-stratified analyses were directionally consistent with this concern under a fixed consistency threshold (Supplementary Table S38). If such reductions affect older patients, they could map onto a population already carrying higher clinical risk, with implications for both safety and fairness. These analyses remain descriptive: without counterfactual demographic perturbation or prospective workflow evaluation, we cannot determine whether the observed gradients arise from benchmark composition, case complexity, documentation effects or model bias. We therefore interpret this as an important deployment consideration requiring dedicated bias auditing rather than as a resolved finding." (Lines 398-410)

Point 1.3 *- Semantic entropy analysis - transparency*

Comment: - In response to my comment on the confidence estimation literature, the authors ran a semantic entropy-inspired analysis (SemanticCertaintyDx: AUC = 0.832 vs ConsistencyDx AUC = 0.860, DeLong p = 0.024, r = 0.86). I respect the decision not to incorporate this into the main text given the high correlation. But the analysis currently exists only in the response letter? it does not appear anywhere in the revised paper, nor the supplementary, at least from what I see. A reader has no way to know it was done or what it found. The result itself is actually reassuring: the two measures converge, and Consistency is statistically superior. I would suggest adding a brief supplementary table or note - not as a primary result, but for transparency. It would also be useful to others working in this space.

Response:

Thank you for this helpful suggestion. We have now reported this analysis in **Supplementary Table S37** for transparency. Specifically, we include the discrimination performance of SemanticCertaintyDx on MIRA-v2, its comparison with ConsistencyDx, and the correlation between the two measures.

As you noted, SemanticCertaintyDx showed strong discrimination (AUC = 0.832), but ConsistencyDx remained superior (AUC = 0.860; DeLong P = 0.024) and the two measures were highly correlated (r = 0.865). We therefore continue not to treat semantic entropy as a secondary, transparently reported measure rather than a primary result in the main framework.

Changes made:

- Added **Supplementary Table S37** reporting the SemanticCertaintyDx analysis on MIRA-v2.

  Table S37 | SemanticCertaintyDx supplementary comparison with ConsistencyDx on MIRA-v2.

<table>
<tr><th>Metric</th><th>AUC</th><th>95% CI</th><th>Diff</th><th>DeLong P</th><th>Pearson Correlation</th></tr>
<tr><td>ConsistencyDx</td><td>0.860</td><td>[0.804,0.908]</td><td rowspan="2">0.029</td><td rowspan="2">0.024</td><td rowspan="2">0.865</td></tr>
<tr><td>SemanticCertaintyDx</td><td>0.832</td><td>[0.771,0.884]</td></tr>
</table>

- Added a brief cross-reference in the **Discussion** noting that this semantic-entropy-inspired comparison was performed.

  "For transparency, we also report a supplementary semantic-entropy-inspired comparison (SemanticCertaintyDx) on MIRA-v2; this metric showed strong discrimination but remained highly correlated with ConsistencyDx, which retained superior performance (Supplementary Table S37)." (Lines 381-384)

Point 1.4 *- Cross-model support for the confidence framework*

Comment: - This is, for me, the one remaining somewhat substantive point: the revision added three new models and demonstrated that competitive diagnostic performance extends across architectures. That is a real and meaningful addition! BUT the confidence framework itself - all ConsistencyDx analyses, all ProbScore and LingCert ROC curves, selective autonomy simulations, the stress test, and the physician adjudication analyses - remains evaluated exclusively on GLM-4.5-Air. Qwen-3.5, which achieves 90.04% accuracy (slightly above the confidence model's 88.42%), was available during revision, yet no confidence metric analyses are reported for it.

The paper's central claim is that consistency-based gating is a deployable reliability framework. For that claim to hold at the level of this journal, I think it needs to be shown - at least partially - that ConsistencyDx behaves comparably as a reliability signal on models other than the one it was developed on. I would not suggest this requires a full replication across all four models. But running ConsistencyDx and the AUC analysis on at least one additional model - Qwen-3.5 seems the natural choice given its accuracy and availability - would meaningfully strengthen the

generalizability argument that currently rests on a single model for the paper's primary contribution. A supplementary table would be sufficient.

Response:

Thank you for this important comment. We agree that although diagnostic performance generalized across model architectures, the primary reliability analyses remained too concentrated on a single model to support the framework-level generalizability claim.

Our original intent in using GLM-4.5-Air for the primary confidence analyses was to keep the agent workflow fixed across experiments and avoid conflating reliability behavior with model-specific implementation differences. However, we agree that presenting only one model in the main reliability framework left this point under-supported. In response, we have now added supplementary cross-model confidence analyses on the three additional open-weight models evaluated in revision: Qwen-3.5, GLM-5, and GPT-OSS.

Specifically, we now report a new **cross-model ROC AUC summary** (**Supplementary Table S34**) showing that the main qualitative pattern was preserved across all three additional models: ConsistencyDx remained the strongest evaluated diagnostic-stream metric in each case. We also added a new cross-model **operating-point analysis** (**Supplementary Table S36**) showing that the retained-accuracy/coverage trade-off for ConsistencyDx was likewise preserved across models, although the precise thresholds associated with a given accuracy–coverage balance varied across models. To make this transparent in the main text, we now state in the Results that the primary reliability analyses used the GLM-4.5-Air baseline configuration to keep the workflow fixed across experiments, and we added explicit cross-references noting that the central ConsistencyDx finding was not unique to that model.

Changes made:

- Added **Supplementary Table S34**, reporting cross-model ROC AUC summaries for Qwen-3.5, GLM-5, and GPT-OSS on MIRA-v2.
- Added **Supplementary Table S36**, reporting cross-model ConsistencyDx accuracy–coverage operating points for the same three models.
- Added an explicit reminder in the **Results** that the primary reliability analyses used the GLM-4.5-Air baseline configuration, consistent with the Methods, to keep the workflow fixed across experiments.

  "*Primary reliability analyses used the GLM-4.5-Air baseline to keep the workflow fixed across experiments.*" (Lines 147-148)

- Added sentences in the **Results** noting that the central **ConsistencyDx** pattern was preserved across the three additional models.

  "A supplementary cross-model analysis on Qwen-3.5, GLM-5 and GPT-OSS further showed that this pattern was not unique to GLM-4.5-Air: across all three additional models,

ConsistencyDx remained the strongest evaluated diagnostic-stream metric (Supplementary Table S34).” (Lines 170-173)

“Across additional models, the retained-accuracy/coverage trade-off for ConsistencyDx was also preserved, although the threshold required to achieve a given balance varied across architectures (Supplementary Table S36).” (Lines 261-264)

Point 1.5 *- ProbScore framing should be made more explicit in the Results*

Comment: - ProbScore presentation is somewhat inconsistent with its revised framing, asthe Methods caveat at lines 833-836 is the right framing, and I appreciate it. But ProbScore still appears prominently in Fig. 3, the correlation matrix (Fig. 4a), and the selective autonomy comparison (Fig. 6b), presented alongside ConsistencyDx as a parallel reliability candidate. The stress-test result - ProbScore remains high, and in pneumonia actually increases, when accuracy degrades - is reported correctly. But the full implication of that finding is not drawn out in the Results narrative.

A reader working through Fig. 3 and Fig. 6 could reasonably conclude that ProbScore is a viable alternative to Consistency for triage gating, which the data do not support. I think a brief sentence in the Results when the stress-test findings are presented - explicitly noting that the behavior of ProbScore under evidence removal (stable or increasing despite accuracy collapse) is inconsistent with what a calibrated confidence signal should do - would bring the presentation into alignment with the Methods framing already established. This is a small addition but would make the message cleaner and more consistent.

Response:

Thank you for this helpful suggestion. We agree. Although the revised Methods already clarified that ProbScore should be interpreted as an internal likelihood proxy rather than as a direct measure of diagnostic certainty, the Results did not yet draw out the full implication of the stress-test findings clearly enough.

In response, we have now added an explicit interpretive sentence in the **stress-test Results** stating that the observed behavior of ProbScore under degraded evidentiary grounding is inconsistent with what would be expected of a calibrated confidence signal: in the perturbed setting, ProbScore could remain stable or even increase despite declining diagnostic accuracy. This addition was intended to make clearer that high absolute score values alone did not reliably track reduced evidentiary support.

We also retained the selective-autonomy comparison with ProbScoreDx for contrast, but now clarify that this comparison is included to show that internal likelihood alone did not provide an equally reliable basis for selective-autonomy routing. Together, these changes bring the **Results** into closer alignment with the more cautious framing already established in the **Methods**.

Changes made:

- Added an explicit interpretive sentence in the **stress-test Results** stating that the behavior of ProbScore under evidence removal is inconsistent with a calibrated confidence signal.

  "*More broadly, this pattern is inconsistent with a calibrated confidence signal: under degraded evidentiary grounding, some scores remained stable or even increased despite declining diagnostic accuracy, indicating that high absolute score values alone did not reliably track reduced evidentiary support.*" (Lines 225-228)

- Clarified in the **selective-autonomy Results** that the ProbScoreDx comparison is presented as a contrast, not as evidence for an equally viable routing signal.

  "*In contrast, ProbScoreDx did not reach this accuracy level within the evaluated thresholds, illustrating that internal likelihood alone did not provide an equally reliable basis for selective-autonomy routing.*" (Lines 248-251)

Reviewer #2

Point 2.1 *- Overall assessment*

Comment: - The authors have adequately addressed my concerns.

Response:

Thank you very much! We appreciate your time and the constructive feedback you provided in the previous round, which we believed helped strengthen the manuscript substantially.

Reviewer #3

Point 3.1 *- Overall assessment of the revision*

Comment: - Thank you for your response. Most of my original issues with the paper was with the over claiming - the conclusions were not supported by the experiments/data. You have largely addressed this by appropriately changing the claims; however some of the broader framing in the introduction remains.

Response:

Thank you for this careful reassessment. We appreciate that you found the major overclaiming concerns substantially improved. We agree that a few broader framing elements in the Introduction still required tightening, and we have revised those passages further in response to your comments.

Point 3.2 *- Introduction framing still implies cases may bypass review*

Comment: - Specifically, the introduction talks about 'when an agent's output is likely reliable enough to act on and when it should be reviewed,' suggesting that in some cases there will be no

human review, despite the author response that states they no longer present confidence-based routing as eliminating the need for oversight. The last sentence in the introduction says "...by helping determine when an agent's output may be reasonable to act on and when it should be escalated to human review.'

Response:

Thank you for this important clarification. We agree that the previous wording could still be read as implying that high-confidence cases bypass continuing review, which was not intended. The introduction has been revised to align with the manuscript's current framing of selective autonomy as a risk-stratification and routing mechanism under continued institutional governance, rather than elimination of oversight.

Specifically, we replaced phrases such as "reliable enough to act on" with wording that distinguishes cases considered for autonomous handling within a governed workflow from cases deferred for clinician review. The revised Introduction now states that clinicians need to know "which outputs may be suitable for autonomous handling within a governed workflow and which should be deferred for clinician review," and that the framework helps determine "which cases may be considered for autonomous handling under continued institutional governance and which should be deferred for clinician review." These changes were made specifically to remove any suggestion that confidence-based routing eliminates the need for continued oversight.

Changes made:

- Revised the **Introduction** to replace "act on" language with wording focused on autonomous handling within a governed workflow versus deferral for clinician review.

  "...clinicians need to know which outputs may be suitable for autonomous handling within a governed workflow and which should be deferred for clinician review." (Lines 56-58)

  "They also support selective-autonomy routing by helping distinguish cases suitable for autonomous handling under continued governance from those requiring clinician review." (Lines 97-99)

Point 3.3 *- Fraction of cases requiring review to avoid "failures"*

Comment: - Since the proposed approach is framed as a risk stratification mechanism (with an AUC=0.86 for ConsistencyDX) it'd be helpful to see this further contextualized. What I want to know is what fraction of the cases need to be reviewed to guarantee no 'failures' i.e., cases where the model was confident but wrong. One can also sweep the review threshold, and as each point measure how many cases would have been confidently wrong (and not undergone review). I believe the ROC and PRC curves capture this additional context and would be good to include in a revision.

Response:

Thank you for this important suggestion. We agree that the selective-autonomy results should be further contextualized in terms of how residual autonomous-stream errors change as the review threshold is varied. In response, we performed a **threshold-sweep analysis** of ConsistencyDx and now report, for each threshold, the retained coverage, deferred fraction, retained accuracy, and the number of observed autonomous-stream errors remaining (**Supplementary Table S35**).

As expected, **within the evaluated threshold range, there was no zero-error operating point**. Eros in the autonomous stream dropped substantially as thresholds became more conservative, but some always remained. Specifically, one benchmark-defined autonomous error remained at ConsistencyDx ≥ 0.95 and also at ConsistencyDx = 1.00. We now state this explicitly in the **Results** and **Discussion**.

We interpret this not as a failure of the threshold-sweep analysis, but as an important property of the framework. Confidence-based routing can meaningfully **reduce** residual error burden, but it does not guarantee failure-free autonomy in the retrospective benchmark setting. Importantly, this residual high-consistency tail was also consistent with the confidence-state case review in **Fig. 4**, which showed that some very high-consistency discordant cases reflected **benchmark-label limitations or single-label under-crediting**, rather than uniformly unsafe reasoning. We therefore present the threshold-sweep analysis as an explicit operating-characteristic characterization of the framework, while clarifying that “zero failures” should not be interpreted as a realistic deployment guarantee.

Changes made:

- Added **Supplementary Table S35**, reporting retained cases, coverage, deferred fraction, retained accuracy, and residual autonomous-stream errors across ConsistencyDx thresholds.

  Table S35 | Review-fraction and residual-error operating points for ConsistencyDx on MIRA-v2.
  Threshold-sweep analysis showing retained cases, coverage, deferred cases, review fraction, residual autonomous-stream errors, deferred-stream errors, and retained accuracy across ConsistencyDx thresholds under baseline conditions on MIRA-v2. This analysis is descriptive and was used to characterize how residual benchmark-defined autonomous failures decline as review becomes more conservative; it was not used to define a deployment guarantee of zero-risk autonomy.

Threshold	Retained n	Coverage	Deferred n	Review fraction	Autonomous errors	Deferred errors	Retained accuracy
0.5	531	0.964	20	0.036	39	13	0.927
0.55	520	0.944	31	0.056	32	20	0.938
0.6	506	0.918	45	0.082	29	23	0.943
0.65	482	0.875	69	0.125	25	27	0.948
0.7	456	0.828	95	0.172	17	35	0.963
0.75	422	0.766	129	0.234	13	39	0.969

0.8	390	0.708	161	0.292	10	42	0.974
0.85	345	0.626	206	0.374	9	43	0.974
0.9	272	0.494	279	0.506	3	49	0.989
0.95	174	0.316	377	0.684	1	51	0.994
1	99	0.180	452	0.820	1	51	0.990

- Revised the **Results** to state explicitly that **no zero-error operating point was observed** within the evaluated threshold range, and that one benchmark-defined autonomous error remained even at ConsistencyDx ≥ 0.95 and 1.00.

  "*A supplementary threshold-sweep analysis showed that residual autonomous-stream errors decreased as review became more conservative, but did not fall to zero within the evaluated ConsistencyDx threshold range (Supplementary Table S35). This residual tail was consistent with the high-consistency discordant cases in Fig. 4, indicating that confidence-based routing can reduce but not eliminate benchmark-defined failures.*" (Lines 256-261)

- Revised the **Discussion** to clarify that this analysis characterizes **risk reduction in a retrospective benchmark setting**, not a guarantee of failure-free real-world autonomy.

  "*The threshold-sweep analysis reinforces this: more conservative review policies reduced residual autonomous-stream errors but did not eliminate them in the benchmark setting. Some of these residual high-consistency errors also reflected benchmark-label limitations rather than uniformly unsafe reasoning, suggesting that zero benchmark discordance is neither a realistic nor a sufficient deployment target.*" (Lines 392-397)

Further Changes

To comply with the journal's formatting requirements for Articles, we additionally condensed the revised manuscript for length while preserving content. These changes were editorial rather than substantive: no previously added analyses, clarifications, or discussion points were removed, and all revisions described in the point-by-point response remain reflected in the manuscript.

We also made a minor title revision, changing "Reliable Decision-Making" to "Reliable Clinical Decision-Making" to better reflect the clinical focus of the manuscript.

Finally, we updated the citation for the MIRA[1] framework manuscript from an in-press reference to the final published Nature article.

1. Ferber, D. *et al.* Towards autonomous medical artificial intelligence agents. *Nature* https://doi.org/10.1038/s41586-026-10675-5 (2026) doi:10.1038/s41586-026-10675-5.
